# Managing Contamination and Diverse Bacterial Loads in 16S rRNA Deep Sequencing of Clinical Samples: Implications of the Law of Small Numbers

**DOI:** 10.1128/mBio.00598-21

**Published:** 2021-06-08

**Authors:** Ruben Dyrhovden, Martin Rippin, Kjell Kåre Øvrebø, Randi M. Nygaard, Elling Ulvestad, Øyvind Kommedal

**Affiliations:** a Department of Microbiology, Haukeland University Hospital, Bergen, Norway; b Section for Bioinformatics, Haukeland University Hospital, Bergen, Norway; c Department of Surgery, Haukeland University Hospital, Bergen, Norway; d Department of Clinical Science, University of Bergen, Bergen, Norway; Department of Veterinary Medicine

**Keywords:** 16S rRNA, acute cholangitis, contamination, NGS, targeted amplicon sequencing, *rpoB*

## Abstract

In this article, we investigate patterns of microbial DNA contamination in targeted 16S rRNA amplicon sequencing (16S deep sequencing) and demonstrate how this can be used to filter background bacterial DNA in diagnostic microbiology. We also investigate the importance of sequencing depth. We first determined the patterns of contamination by performing repeat 16S deep sequencing of negative and positive extraction controls. This process identified a few bacterial species dominating across all replicates but also a high intersample variability among low abundance contaminant species in replicates split before PCR amplification. Replicates split after PCR amplification yielded almost identical sequencing results. On the basis of these observations, we suggest using the abundance of the most dominant contaminant species to define a threshold in each clinical sample from where identifications with lower abundances possibly represent contamination. We evaluated this approach by sequencing of a diluted, staggered mock community and of bile samples from 41 patients with acute cholangitis and noninfectious bile duct stenosis. All clinical samples were sequenced twice using different sequencing depths. We were able to demonstrate the following: (i) The high intersample variability between sequencing replicates is caused by events occurring before or during the PCR amplification step. (ii) Knowledge about the most dominant contaminant species can be used to establish sample-specific cutoffs for reliable identifications. (iii) Below the level of the most abundant contaminant, it rapidly becomes very demanding to reliably discriminate between background and true findings. (iv) Adequate sequencing depth can be claimed only when the analysis also picks up background contamination.

## INTRODUCTION

Microbial DNA contamination from extraction kits and other PCR and sequencing reagents (background bacterial DNA) is a major challenge in 16S rRNA amplicon sequencing (16S deep sequencing) of polymicrobial infections (1, 2). Several studies have demonstrated the risk for erroneously interpreting contaminating DNA as bacteria originating from the sample (1, 3–5). In clinical microbiology, reporting a contaminant species as a relevant pathogen may cause unnecessary antibiotic treatment or even falsely classify a noninfectious condition as a bacterial infection. Unfortunately, many of the studies on infectious disease materials do not address background contamination (6–8), and among those that do, the approaches vary. The most used method is to sequence extraction controls along with the samples and remove those bacteria from the sample reports which were also found in the controls (9–12). However, the sensitivity of this method is reduced if bacteria truly present in clinical samples are also present in the negative controls (2). Further, the specificity of this approach relies on the assumption that sequencing of the negative controls provides an exhaustive identification of contaminants.

Within microbiota research, a range of methods have been developed to diminish the problem of background contamination (1, 2, 4, 13, 14), but many of these approaches are not easily transferable to diagnostic laboratories. Despite the common aim of describing bacterial flora, clinical microbiologists and microbiota researchers have partly divergent challenges and goals. In microbiota research, typically large sets of the same sample type are analyzed in multiple batches over a limited period. Combined with extensive use of negative and positive controls, and even multiple sequencing techniques (14), this allows labs to use pattern recognition and statistical calculations to filter their data sets (4). Although they make considerable effort to ensure the overall quality of a data set, there is less focus on the individual sample, and identifications are usually limited to the genus level or above. In clinical microbiology, there is a broad spectrum of sample types with highly divergent bacterial concentrations and compositions, and background contamination will vary over time with different batches of reagents and consumables. The focus is always the individual patient and species level identification is normally required. Finally, time to results and cost are crucial matters, limiting the room for extensive assessments of background contamination.

Accurate filtering of background contamination is more critical in weakly positive samples, where it constitutes a larger portion of the total bacterial DNA (1, 2). Sequencing depth is another essential factor, in particular for strongly positive, polymicrobial samples where the use of too few reads may result in failure to detect low abundance species. In clinical microbiology, especially in samples from normally sterile body sites, the detection of a bacterium at any concentration is *a priori* considered potentially relevant. A sample from a polymicrobial infection must be considered a snapshot of a potentially dynamic process, and species present at low abundances in the sample can flourish at the site of infection at a later stage, especially if antibiotic treatment is directed only against the dominant flora. Also, the relative microbial abundances in a sample cannot be assumed to be representative of the entire site of infection. For example, the abundance of a given species in pus aspirated from the necrotic, anaerobic center of an abscess is not necessarily representative of the abundance of the same species in the more oxygenated periphery on the transition to intact tissue. Despite these issues, there has been little attention to the relationship between sequencing depth and sensitivity.

In this study, we aim to describe and evaluate simple and transparent approaches for dealing with contamination in 16S deep sequencing in clinical microbiology. We base our suggestions on the observation that the presence of a few dominant contaminant species is highly consistent across all controls, while in the same controls the presence of less dominant contaminant species seems to vary (15, 16). We use these most abundant contaminant species and their abundances in the corresponding clinical samples to set sample-specific cutoffs for the number of reads needed to reliably classify a species as a noncontaminant. We perform repeat sequencing of a set of extraction controls, both before and after the 16S rRNA PCR amplification step, to underpin our approach and to demonstrate sensitivity limitations that remain even in deep sequencing. We further test the approach on a diluted, standardized staggered mock community and on prospectively collected bile samples from patients with acute cholangitis or noninfectious bile duct stenosis. To demonstrate the importance of sequencing depth, all patient samples were sequenced twice with different sequencing depths in each replicate.

## RESULTS

### Experiment 1. Repeat sequencing of extraction controls.

**FIG 1:**
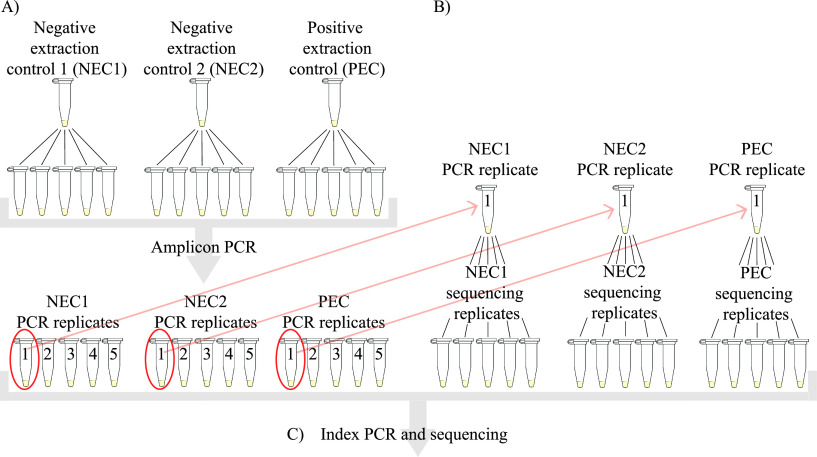
Illustration of workflow for PCR amplification and sequencing of the three extraction controls. (A) All three samples were split into five replicates before 16S rRNA amplicon PCR, resulting in five PCR replicates from each original sample after the PCR. (B) From each of the three groups of PCR replicates, one of the five replicates was then split into five new replicates. (C) Index PCR and sequencing were than performed for both PCR replicates and sequencing replicates on the same sequencing run. One PEC sequencing replicate was lost due to technicalities, leaving 15 PCR replicates and 14 sequencing replicates eligible for postsequencing analysis.

**(i) Experimental design.** We first sought to understand the mechanisms behind the observed phenomenon that the presence of a few dominant contaminant species is highly consistent across all controls, while the presence of less dominant contaminant species seems to vary (15, 16). To investigate this, we analyzed a set of extraction controls in a separate sequencing run (Fig. 1). Three different samples were analyzed, two negative extraction controls (NEC1 and NEC2), consisting of PCR-grade water and lysis buffer, and one weakly positive extraction control (PEC) containing Legionella pneumophila. To isolate the impact of the PCR amplification of the sample template (amplicon PCR) from the impact of the following index PCR and sequencing procedure, each of the three controls was split into five replicates before the amplicon PCR (hereafter named “PCR replicates”). One PCR replicate from each of the three controls was further split into five replicates before sequencing (hereafter named “sequencing replicates”). All PCR and sequencing replicates were then indexed and sequenced in the same run. We used the results from this part of the study to formulate criteria for filtration of sequencing data from clinical samples, which we further evaluated on a staggered mock community and a collection of bile samples.

**FIG 2:**
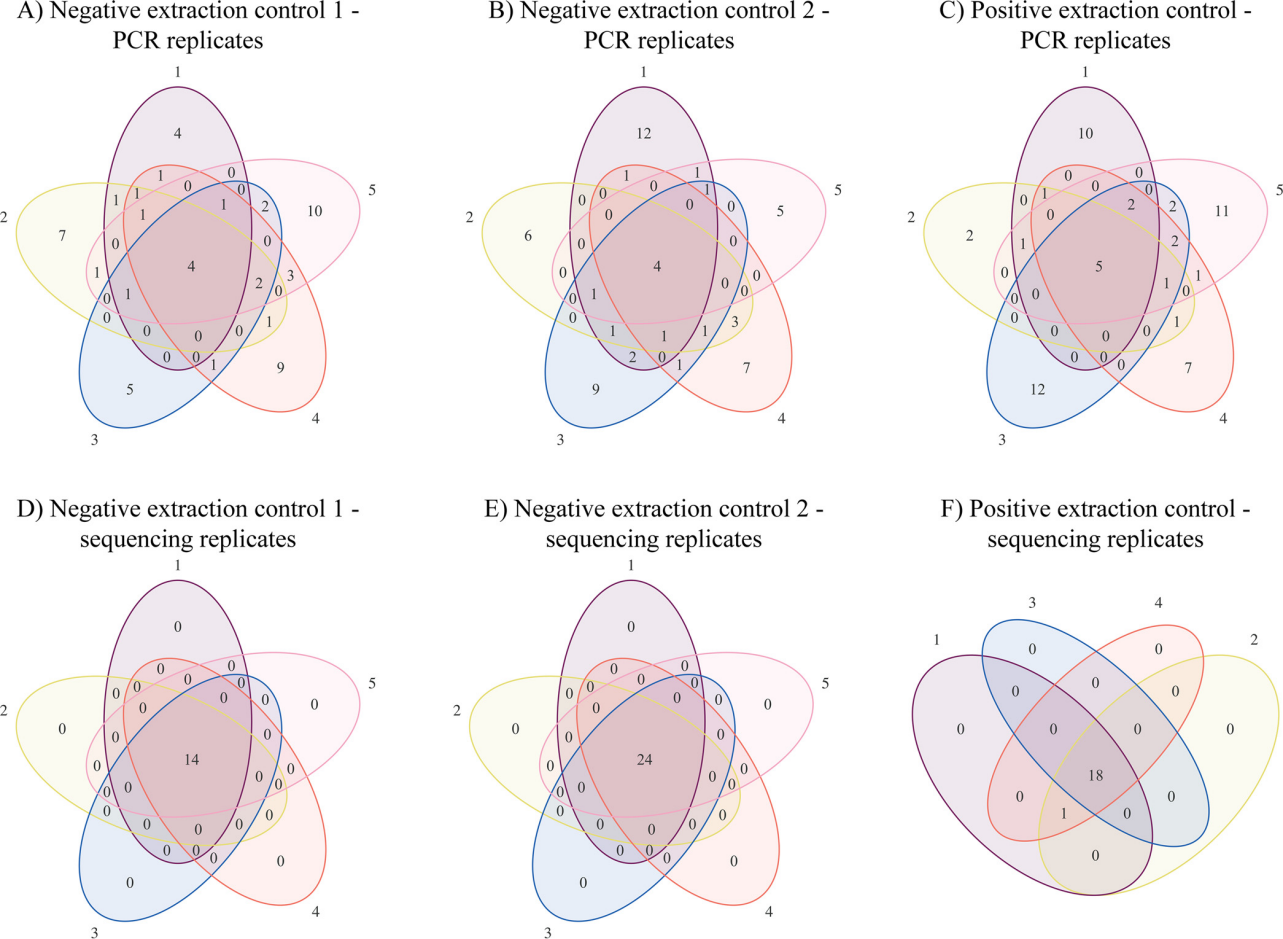
Venn diagram of bacterial identifications in PCR replicates (A to C) and sequencing replicates (D to F), showing a high diversity between the PCR replicates originating from the same sample, in contrast to the high similarity between the sequencing replicates originating from the same PCR. (A) Fifty-five different bacteria were found in all five NEC1 PCR replicates combined. Only 4 (7%) identifications were shared between all five replicates, while 35 (64%) were found in only 1 of the 5 replicates. (B) Fifty-six different bacteria were found in all NEC2 PCR replicates in total. Only 4 (7%) identifications were detected in all five replicates, and 39 (70%) identifications were found in only a single replicate. (C) Fifty-eight different bacteria were found in all PEC PCR replicates in total. Five (9%) bacterial identifications were shared across all five replicates, including Legionella pneumophila, which was the positive control. Forty-two (72%) identifications were detected in only a single replicate. (D and E) Bacterial identifications were identical in all sequencing replicates of both NEC1 and NEC2. (F) Bacterial identifications were identical in all sequencing replicates of PEC except for one *Phenylobacterium* species which was identified in only three out of the four replicates.

**(ii) Results.** The Venn diagrams in Fig. 2 illustrate the higher diversity between PCR replicates compared to the sequencing replicates. Among the five PCR replicates of one sample, most species were present in only a single replicate. Only four (NEC1 and NEC2 PCR replicates) or five (PEC PCR replicates) species were found in all five replicates. In contrast, there were no differences between sequencing replicates originating from the same PCR except for a single species missing in one replicate. This bacterium, a *Phenylobacterium* sp., was also present in the latter replicate but by less than 50 reads and consequently below our cutoff for a valid identification.

**FIG 3:**
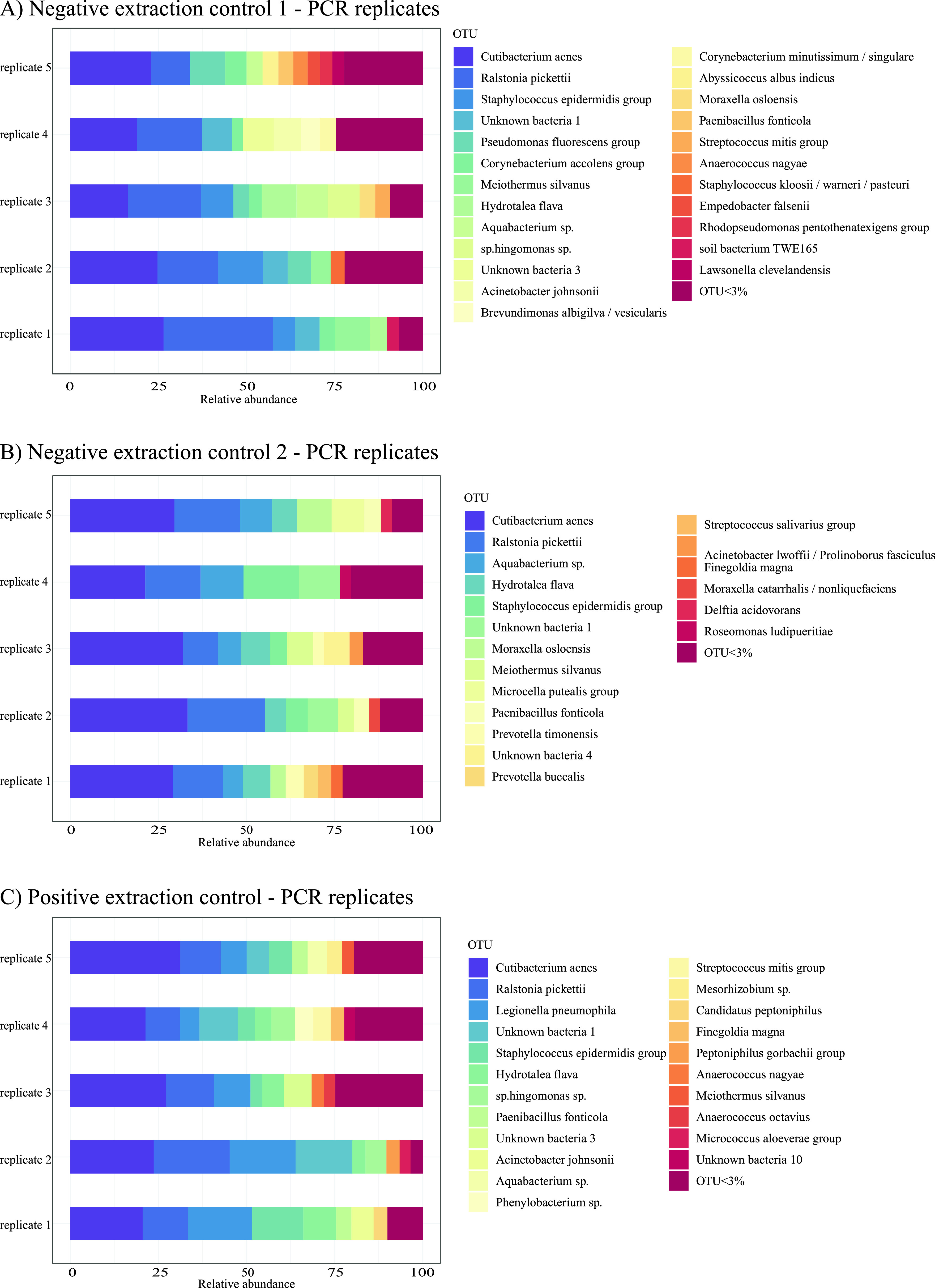

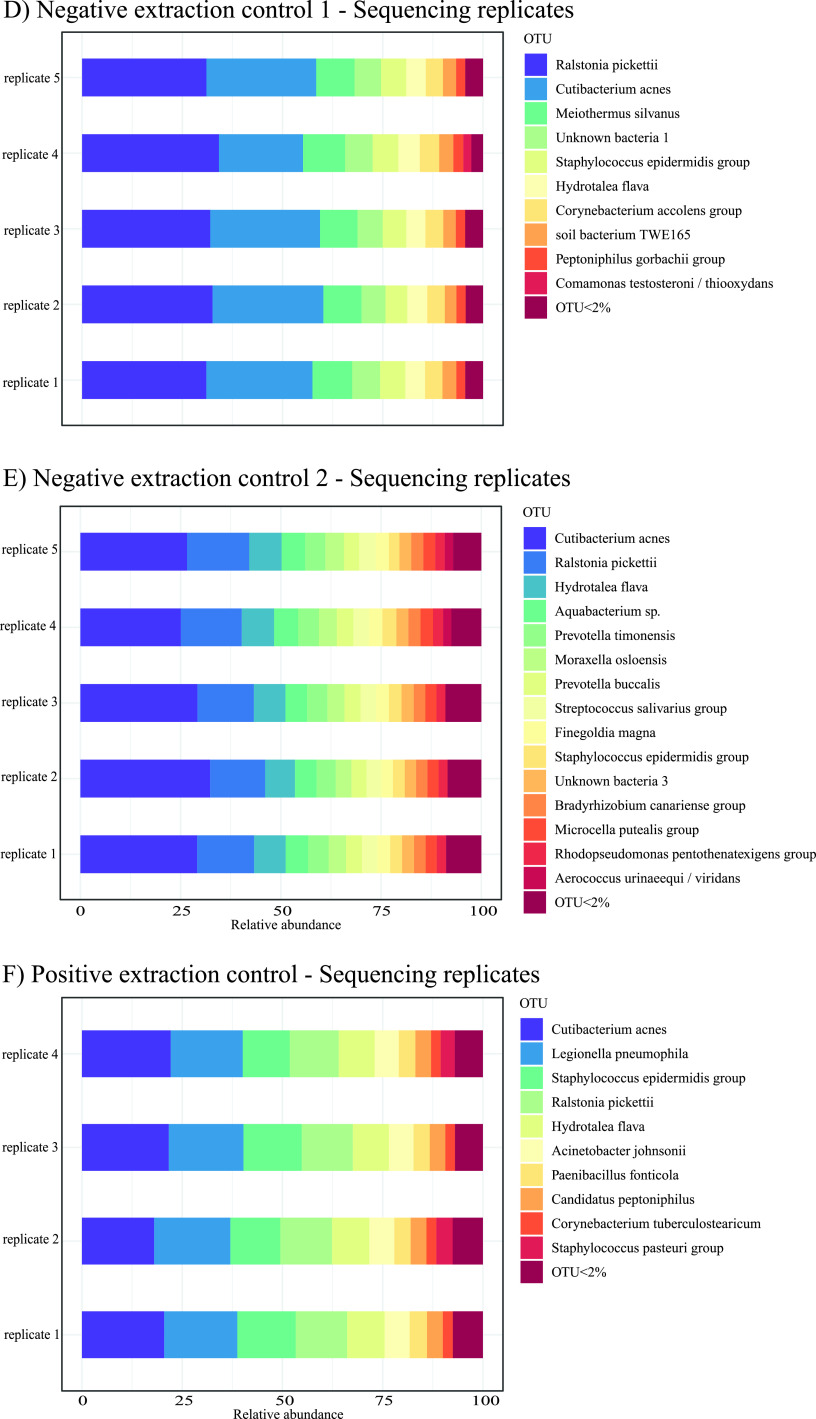
Bacterial composition in PCR (A to C) and sequencing (D to F) replicates of the extraction controls. A few species dominated in all replicates. (A to C) The replicates from each sample show a high degree of variability. Only two species were present in every PCR replicate from all three samples: Ralstonia picketti and Cutibacterium acnes. These two species were also the most abundant species in all PCR replicates. (D to F) Bacterial identifications were identical in all sequencing replicates of NEC1 and NEC2, while one *Phenylobacterium* species was identified in only three out of the four replicates of PEC. In those three replicates containing *Phenylobacterium*, it appeared with the lowest number of reads (105, 51, and 90) and relative abundance (0.03%, 0.02%, and 0.04% of total number of reads, respectively) of all bacterial identifications.

A few species dominated all replicates, whereas the majority of contaminants appeared at relatively low abundances. The most abundant bacterium in each replicate was represented by 19 to 33% of the total number of valid reads (Fig. 3). In all replicates, Ralstonia pickettii and Cutibacterium acnes were the two most dominant species. These were also the only bacteria present in all replicates from all groups.

**FIG 4:**
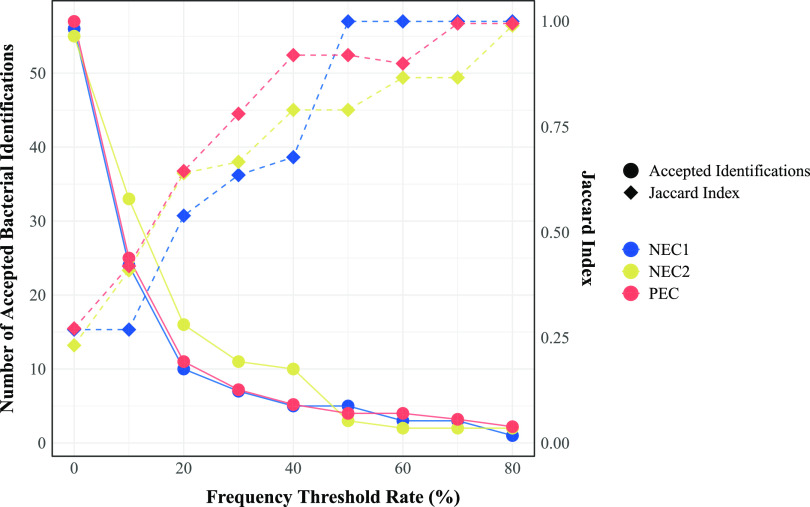
Graph showing the correlation between a chosen frequency threshold rate and the resulting number of accepted bacterial identifications and similarity between PCR replicates. The *x* axis shows the frequency threshold rate (FTR) calculated as a percentage of the most dominant contaminant bacteria in each replicate measured in the number of sequencing reads. The left *y* axis shows the total number of accepted bacterial species for all five PCR replicates for each control when only bacteria represented by more reads than the chosen FTR were accepted. The right *y* axis shows the mean sample to sample Jaccard index of the five PCR replicates when only bacteria represented by more reads than the chosen FTR cutoff were accepted.

In Fig. 4, we have defined a “frequency threshold rate” (FTR) as a percentage of the most dominant contaminant bacteria in each replicate measured in the number of sequencing reads. For example, if the most abundant contaminant bacterium is present in 10,000 reads, then the 20% FTR is 2,000 reads. Figure 4 shows a steep decrease in the number of bacterial identifications with an abundance above the FTR as the rate increases from 0% to 50%, from >55 bacteria to  ≤5 bacteria in each group of replicates. The plot also shows the correlation between the FTR and the similarity between the PCR replicates. The chance for the same bacterial species to be found in all PCR replicates increases as the relative abundance of that bacterium increases. For NEC1, bacteria with abundances above 50% of the dominant contaminant were present in all replicates. The corresponding thresholds for NEC2 and PEC were 80% and 70%, respectively.

On the basis of these findings, we suggest the following criteria for filtering bacterial contaminants in clinical samples. (i) Any bacterium appearing with a higher abundance than the top five abundant contaminants, as determined by the sequencing of negative and positive extraction controls, is accepted as a valid identification, even if it occurs as a low abundance species in the controls. (ii) Bacteria present in frequencies between 20% and 100% of the most abundant contaminant are accepted as likely valid identifications, but only if they are also absent from all the negative controls. (iii) Bacteria present in frequencies below 20% of the most abundant contaminant are always rejected as invalid. (iv) In samples where none of the top five abundant contaminants are detected, all identifications are accepted as valid.

Detailed data from these experiments, including technical sequencing results and sample diversity measures, is provided in Table S1 of the supplemental material. Operational taxonomy unit (OTU) lists for all extraction control replicates can be found in Table S2.

10.1128/mBio.00598-21.1 TABLE S1 (A) Mean number of accepted reads, number of bacterial identifications, and beta diversity measures for each group of extraction control replicates in experiment 1. (B) Number of accepted reads and alpha diversity measures for all extraction control replicates in experiment 1. (C) Sample-to-sample beta diversity measures for all extraction control replicates in experiment 1. Download Table S1, PDF file, 0.2 MB. Copyright © 2021 Dyrhovden et al. 2021 Dyrhovden et al. https://creativecommons.org/licenses/by/4.0/ This content is distributed under the terms of the Creative Commons Attribution 4.0 International license.

10.1128/mBio.00598-21.2 TABLE S2 OTU table of all extraction control replicates in experiment 1. Download Table S2, XLSX file, 0.02 MB. Copyright © 2021 Dyrhovden et al. 2021 Dyrhovden et al. https://creativecommons.org/licenses/by/4.0/ This content is distributed under the terms of the Creative Commons Attribution 4.0 International license.

### Experiment 2. Sequencing of a staggered mock community.

**(i) Experimental design.** Our next experiment included sequencing of a staggered mock community together with negative and positive extraction controls. The aims of this experiment were twofold: (i) to assess the actual abundance of the contaminants detected in our negative controls and to determine at what level the observed high variability in PCR replicates occur and (ii) to assess the sensitivity and specificity of our suggested criteria for filtering bacterial contaminants and to compare it to other common methods for contaminant filtering.

We performed deep sequencing of three different dilutions of the staggered mock community: a 1:10 dilution, representing a high bacterial load sample (16S PCR threshold cycle [*C_T_*] value of 11.2), and a 1:10^5^ and a 1:10^6^ dilution, representing low bacterial load samples (16S PCR *C_T_* values of 27.3 and 31.7, respectively). The theoretical composition of bacterial cells and the estimated 16S rRNA copy counts in each of these dilutions is presented in Table S3. The 1:10 dilution was split into two PCR replicates (1:10_1 and 1:10_2) and the 1:10^5^ dilution and 1:10^6^ were split into four PCR replicates each (1:10^5^_1 to 1:10^5^_4 and 1:10^6^_1 to 1:10^6^_4) before the PCR amplification step. A negative and a positive extraction control were split into five PCR replicates each before the PCR amplification step and sequenced together with the mock community samples.

10.1128/mBio.00598-21.3 TABLE S3 The theoretical composition of bacterial cells and the 16S rRNA copy counts in each of the mock community dilutions. Download Table S3, PDF file, 0.2 MB. Copyright © 2021 Dyrhovden et al. 2021 Dyrhovden et al. https://creativecommons.org/licenses/by/4.0/ This content is distributed under the terms of the Creative Commons Attribution 4.0 International license.

**FIG 5:**
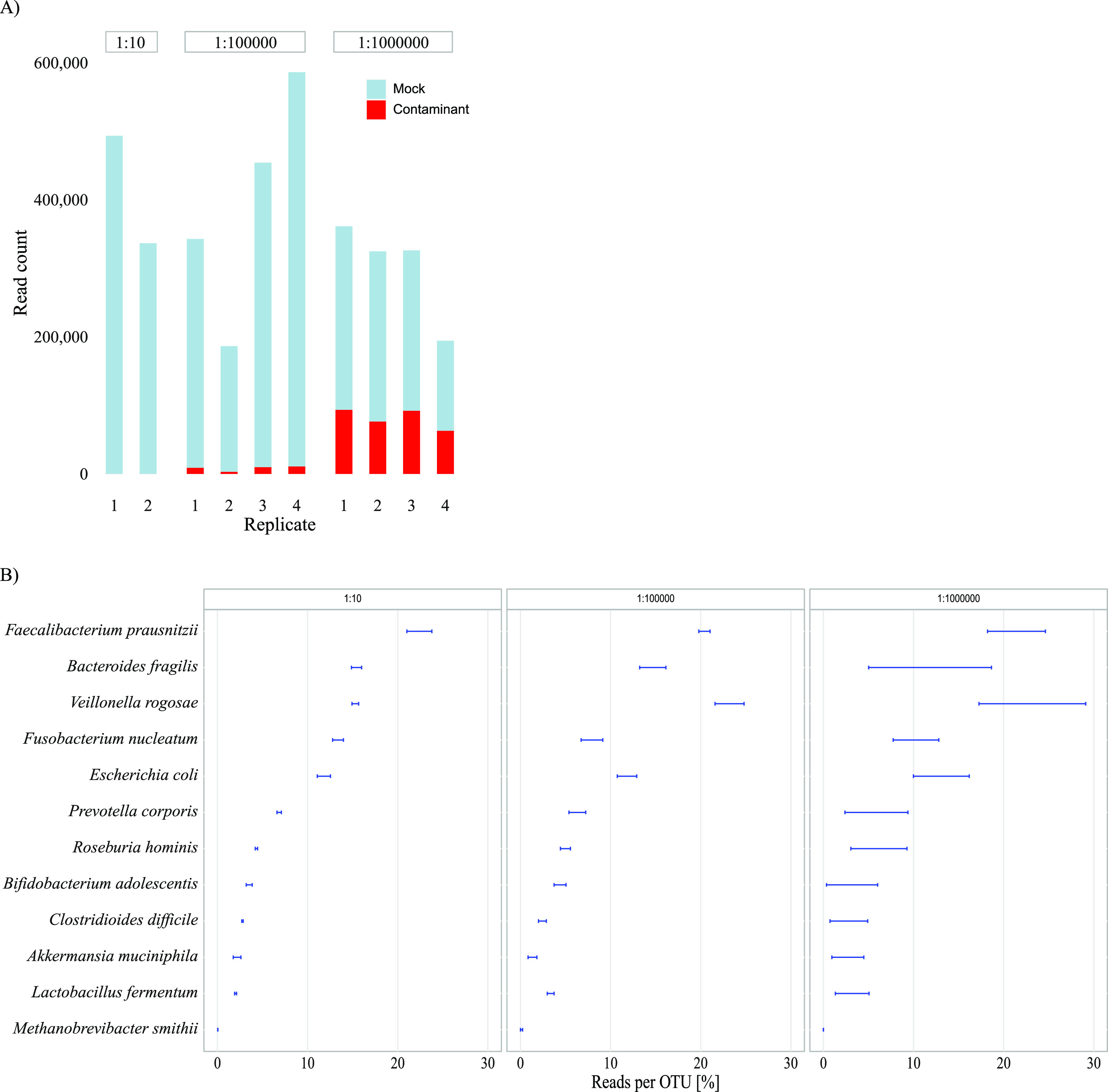
Analysis of mock community dilutions. (A) Number of reads per sample and distribution of reads from mock community and DNA contaminants. The absolute and relative amount of reads from DNA contaminants increases with the subsequent dilutions. (B) Identified mock microbes in each of the three dilutions investigated, and the variation (range) in relative abundance of each identified bacteria between the different PCR replicates within each dilution. The species identified in the most diluted sample showed a higher variation in relative abundance between PCR replicates.

**(ii) Results.** *(a) Mock community result variability.* The total number of accepted reads from the mock community samples after quality filtering was 3,606,622. Each sample had between 186,375 and 586,295 reads, and the relative proportion of DNA contaminants increased with subsequent dilutions (Fig. 5A). No contaminant microbes were identified in the high bacterial load sample (Fig. 5A). In the replicates from the 1:10^5^ dilution, contamination constituted 2 to 3% of the total number of reads, increasing to 24 to 32% in the 1:10^6^ dilution replicates. Figure 5B shows the variation in relative abundance between the PCR replicates for each identified mock community bacterium and how the variability increases in the higher dilutions. Twelve of the 15 bacterial species present in the mock community were identified in each of the 1:10 diluted replicates, representing 100% of the identified OTUs in both samples. The three mock community species that were not detected were those with the lowest abundances, ranging from 0.00009% to 0.0065% of the total microbial content of the mock community (Table S3). In the 1:10^5^ dilution, the same 12 bacterial species were identified in two out of four PCR replicates, while the least abundant of these species, Methanobrevibacter smithii, remained undetectable in two PCR replicates. In the 1:10^6^ dilutions, the 11 most abundant mock community species were identified in all four PCR replicates. An OTU table for all mock community PCR replicates and extraction controls is provided in Table S4.

10.1128/mBio.00598-21.4 TABLE S4 OTU table of all mock community replicates and extraction control replicates in experiment 2. Download Table S4, XLSX file, 0.02 MB. Copyright © 2021 Dyrhovden et al. 2021 Dyrhovden et al. https://creativecommons.org/licenses/by/4.0/ This content is distributed under the terms of the Creative Commons Attribution 4.0 International license.

**FIG 6:**
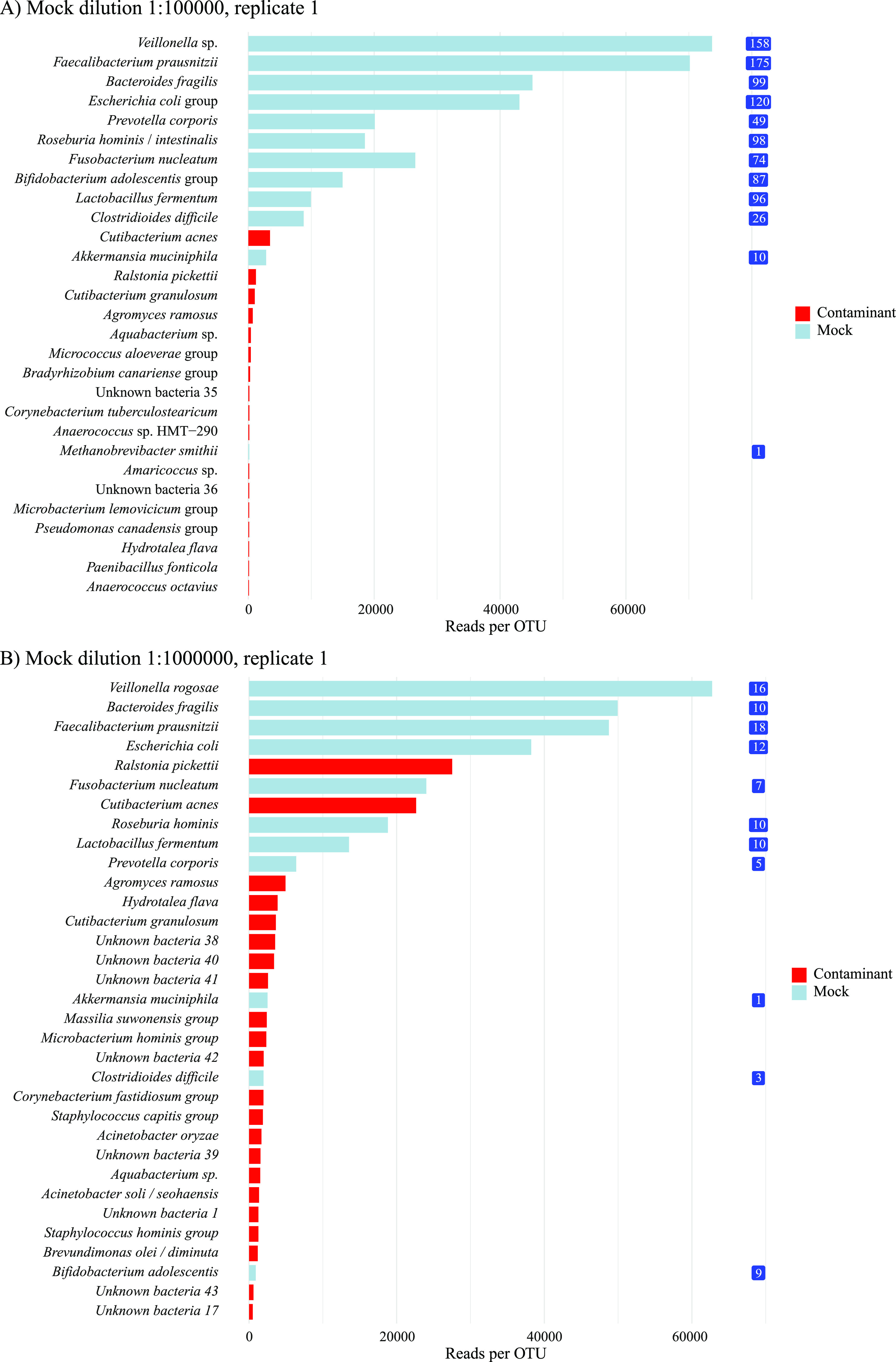
The abundance of all species found in the first replicate of the 1:10^5^ and 1:10^6^ dilutions. The theoretical numbers of 16S copies of each mock community species in 2 μl PCR template are shown as white numbers on blue rectangles. The most dominating contaminants were found in the same concentration as mock microbes with a theoretical concentration of approximately 10 16S copies per 2 μl PCR template. This corresponds to approximately 100 cells per ml in the original sample, or about 500 16S copies per 100 μl extracted DNA. The less abundant contaminants appear in the same concentration as mock microbes with a theoretical concentration close to or less than only a single 16S copy per 2 μl PCR template. This corresponds to an initial concentration of less than 100 bacterial cells per ml sample.

*(b) Assessment of the abundance of laboratory contamination.* The absolute abundances of all OTUs found in the first replicate of the 1:10^5^ and 1:10^6^ dilutions are presented in Fig. 6. Using the calculated concentration of mock community species as a reference, we see that the most dominating contaminants appeared at concentrations around 10 16S copies per 2 μl template, corresponding to about 500 cells per ml in the original sample (Table S3). The less abundant contaminants appeared in concentrations close to or less than a single 16S copy per 2 μl PCR template, approaching the lower limit of detection in the PCR. This corresponded to an initial concentration of up to 100 bacterial cells per ml sample (Table S3).

*(c) Composition of the five negative and five positive extraction control replicates.* The mean number of species identified in each of the extraction controls were 18 (range, 12 to 23) with C. acnes and R. picketti being the only species consistently detected in all negative and positive extraction control replicates. As in experiment 1, these two species were the most dominant contaminants in all replicates, and we observed the same high diversity between PCR replicates originating from the same extraction control. Eighty-three different species were found in the 10 replicates combined (Table S4). The mean Jaccard distance was 0.80 (range, 0.65 to 0.87) for the negative extraction control replicates and 0.76 (range, 0.65 to 0.81) for the positive extraction control replicates. Forty out of 58 species from the negative extraction controls were found in a single replicate only. The corresponding number for the positive extraction control replicates was 35 out of 52.

**FIG 7:**
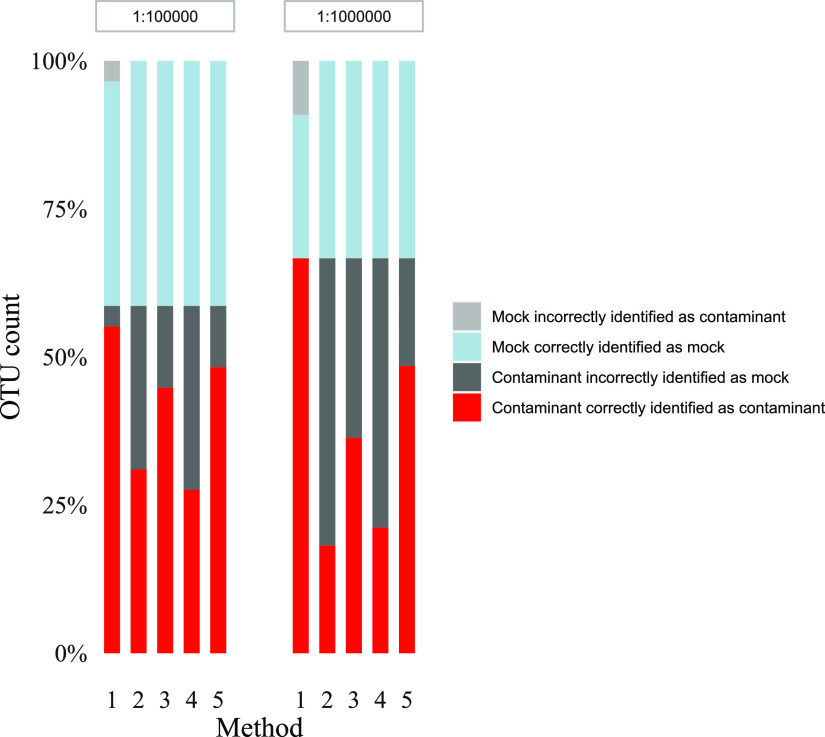
Comparison of five different methods for filtering DNA contaminants for a 1:10^5^ and 1:10^6^ dilution replicate of the mock community. Method 1 is our suggested method. Method 2 is filter all OTUs found in one NEC and PEC. Method 3 is filter OTUs found in all 10 extraction controls. Method 4 is Decontam prevalence based isContamintant function. Method 5 is Decontam prevalence-based *isNotContaminant* function.

*(d) Filtering contaminants based on our suggested criteria versus other common methods for contaminant filtering.* For the first replicate of the 1:10^5^ and 1:10^6^ mock community dilutions, five different methods for removing contaminants were evaluated: (i) our suggested criteria, (ii) removing all OTUs found in one preselected negative and one preselected positive extraction control replicate, (iii) removing all OTUs found in all five negative extraction control PCR replicates and all five positive extraction control PCR replicates, and (iv and v) use of Decontam prevalence-based contaminant identification, including both the *isContaminant* and the *isNotContaminant* function which are both recommended for low biomass samples (4). Results are presented in Fig. 7. Filtering using our suggested criteria had a sensitivity and specificity for the identification of mock community bacteria in the two dilutions combined of 83% and 97% with an overall test accuracy of 93%. One out of 39 contaminants were wrongly classified as a mock community microbe, and four mock community microbes were wrongly classified as contamination (M. smithii in the 1:10^5^ dilution, and Bifidobacterium adolescentis, Clostridioides difficile, and Akkermansia muciniphila in the 1:10^6^ dilution). Filtering using a single preselected negative and positive extraction control gave a sensitivity of 100%, a specificity of 39%, and a test accuracy of 61%. Filtering using all 10 extraction controls had a sensitivity of 100%, a specificity of 64%, and a test accuracy of 77%. Filtering using Decontam *isContaminant* function had a sensitivity of 100%, a specificity of 39%, and a test accuracy of 61%. Filtering using Decontam *isNotContaminant* function increased specificity to 77%, giving a test accuracy of 86%.

**Sequencing of bile samples from patients with acute cholangitis and bile duct stenosis.** Forty-one patients with either acute cholangitis (*n* = 15) or noninfectious bile duct stenosis caused by bile duct stones (*n* = 26) were analyzed. Patient characteristics together with culture and sequencing results are summarized in Table 1. Bacterial loads were categorized as high in 15 samples, moderate in 8, and low in 18 (Table 2). Each sample was split into two replicates before 16S rRNA sequencing and were sequenced using different sequencing depths (16S rRNA replicate 1 and 16S rRNA replicate 2). Table 3 gives an overview of technical sequencing results for the two sets of replicates. Five sequencing runs were performed to include all samples.

**TABLE 1:** General characteristics, culture, and sequencing results of all included patients

Characteristic, culture, or sequencing result	No. (%) of patients or indicated value for characteristic or result	
	Acute cholangitis	Noninfectious bile duct stenosis
No. of patients	15	26
		
General characteristics		
Male	9 (60)	7 (27)
Mean age, yrs	73	54
SD; median; range	10; 71; 58–95	17; 51; 20–83
Previous biliary interventions	5 (33)	10 (39)
ERCP with papillotomy	4 (27)	3 (12)
ERCP without papillotomy	0	1 (4)
Choledocus stent still in place	1 (7)	0
Choledocus stent removed	0	1 (4)
Cholecystectomy	0	7 (27)
Ongoing antibiotic therapy at time of sampling	14 (93)	2 (8)
Concomitant acute pancreatitis	1 (7)	0
Concomitant acute cholecystitis	3 (20)	0
		
Culture and sequencing results		
* C_T_* value^*a*^ for sample, mean	19.8	25,6
SD; median; range	5,1; 19.2; 12.5–27.9	7.5; 28.4; 12.2–33.4
* C_T_* value^*a*^ for NEC^*b*^, mean	32.7	33.4
SD; median; range	1.3; 32.9; 29.7–34.6	1.1; 33.7; 31.3–35.5
Growth in blood culture (of tested)	4 (7)	
Samples with detected bacteria by sequencing^*c*^	15 (100)	21 (81)
Samples with growth of bacteria in bile culture	14 (93)	17 (65)
Polybacterial samples by culture	8 (53)	9 (35
Polybacterial samples by sequencing^*c*^	14 (93)	15 (58%)
Mean species richness by sequencing^*c*^	5,7	8,1
SD; median; range	3.1; 6.0; 1–13	12.8; 3.0; 0–59
Mean species richness by culture	2.1	1.7
SD; median; range	1.4; 2,0; 1–6	1.8; 1,0; 0–5

a Cycle threshold of SYBR green real-time 16S rRNA PCR.

b Negative extraction control.

c All valid and likely valid identifications included, irrespective of whether they were identified in only one or in both of the two 16S rRNA replicates.

**TABLE 2:** Samples where contaminant bacteria were identified, categorized by the bacterial load of the sample

Bacterial load of sample	No. of samples where contaminant bacteria were identified	
	Replicate 1	Replicate 2
High (*n* = 15)	0	0
Moderate (*n* = 8)	4	6
Low (*n* = 18)	18	18
		
Total (*n* = 41)	22	24

**TABLE 3:** Overview of the two 16S rRNA sequencing replicates of the 41 bile samples

Characteristic(s)	16S rRNA replicate 1	16S rRNA replicate 2	*P* value^*c*^
Valid reads,^*a*^ mean (median)	67,608 (50,999)	229,904 (198,331)	<0.001
Range	16,179–221,713	52,068–583,052	
Accepted reads when identified contaminants excluded, mean (median)	53,289 (33,192)	185,522 (166,919)	<0.001
Range	0–221,713	0–583,052	
Total no. of bacterial identifications^*b*^	208	291	
Mean no. of bacterial identifications per sample^*b*^ (median)	5.1 (3.0)	7.2 (4.0)	<0.001
Range	0–26	0–59	

a Accepted reads per sample after quality filtering.

b After exclusion of identified contaminants.

c Student’s *t* test for continuous, normal distributed variables. Mann-Whitney U-test for continuous, skewed variables.

*(a) Identifying and filtering bacterial contaminants in the 16S rRNA replicates.* The combined number of extraction controls analyzed in all clinical sequencing runs were 18. An OTU table for all these is provided as Table S5. The top five abundant species in each extraction control in each of the sequencing runs were identified. If any of these were found in a clinical sample, the most abundant of them defined a level from where contamination could be expected to occur in that sample and were used to filter contaminants as described for experiment 1. Based on this, OTUs were categorized as either valid, likely valid, or contaminant. One or more of the most abundant contaminant species from the controls were identified in 22 and 24 of the 41 samples in the two 16S PCR replicate runs, respectively (Table 2). As shown in Table 2, detection of contaminant bacteria was inversely correlated with the bacterial load of the samples.

10.1128/mBio.00598-21.5 TABLE S5 OTU table of extraction controls in the five 16S rRNA sequencing runs of bile samples. Download Table S5, XLSX file, 0.02 MB. Copyright © 2021 Dyrhovden et al. 2021 Dyrhovden et al. https://creativecommons.org/licenses/by/4.0/ This content is distributed under the terms of the Creative Commons Attribution 4.0 International license.

*(b) Comparison of 16S rRNA PCR replicates from the clinical samples.* The total number of accepted identifications for all samples in 16S replicate 1 was 209 (173 valid and 36 likely valid). The corresponding number for replicate 2 was 295 (239 valid and 56 likely valid). The mean species richness was significantly higher in replicate 2 (Table 3). Figure S1 in the supplemental material shows a prevalence bar chart per sample for 16S rRNA replicate 2, categorized according to our filtering criteria. Verifications by other methods (culture, corresponding 16S rRNA replicate, or *rpoB* sequencing) are also indicated in the figure. An OTU table for all samples in both replicates is provided in Table S6.

10.1128/mBio.00598-21.6 TABLE S6 OTU table of both sequencing replicates of the 41 bile samples. Download Table S6, XLSX file, 0.1 MB. Copyright © 2021 Dyrhovden et al. 2021 Dyrhovden et al. https://creativecommons.org/licenses/by/4.0/ This content is distributed under the terms of the Creative Commons Attribution 4.0 International license.

10.1128/mBio.00598-21.10 FIG S1 Prevalence bar chart per sample for 16S rRNA replicate 2, categorized according to our contaminant filtering criteria. OTUs verified by other methods (culture, corresponding 16S rRNA replicate, or *rpoB* sequencing) are also marked in the figure. Download FIG S1, PDF file, 0.5 MB. Copyright © 2021 Dyrhovden et al. 2021 Dyrhovden et al. https://creativecommons.org/licenses/by/4.0/ This content is distributed under the terms of the Creative Commons Attribution 4.0 International license.

**FIG 8:**
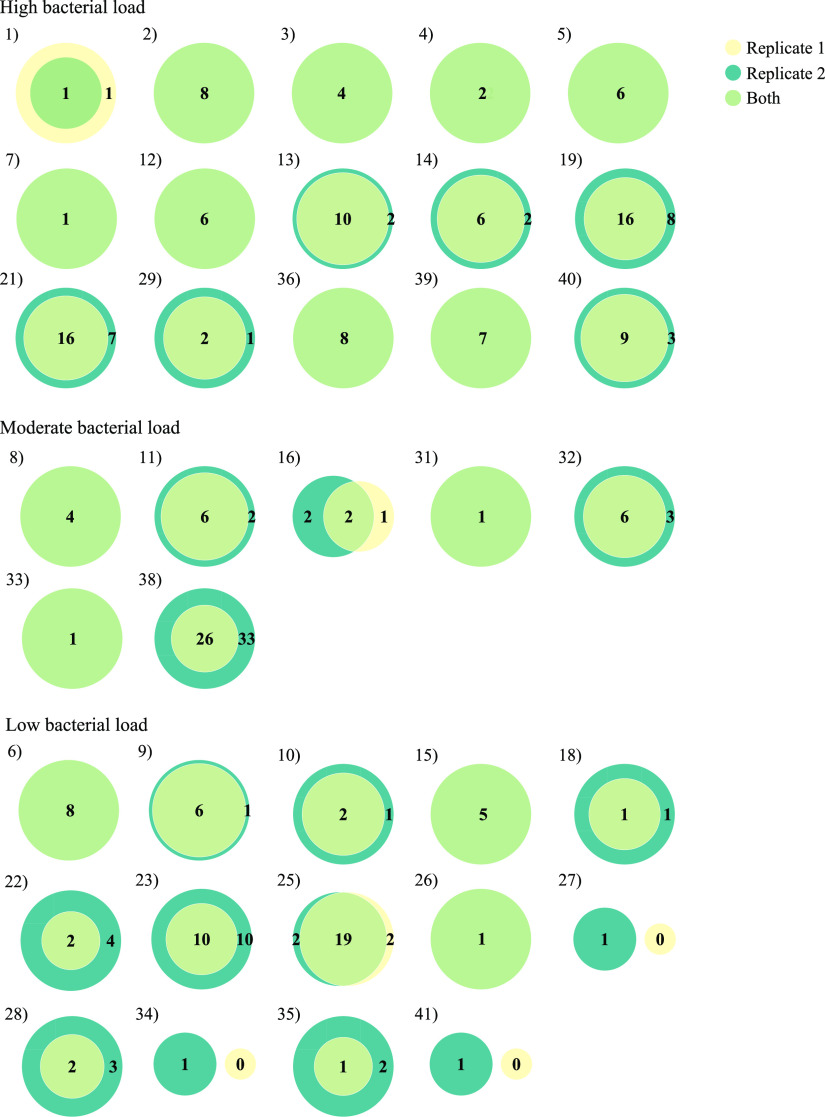
Venn diagram for each clinical sample, comparing the bacterial findings in the two sequencing replicates. There were discrepancies in bacterial findings between the two replicates for 22 (53.7%) of the 41 samples. Ninety-four bacterial identifications were made in only one of the two replicates. Out of these, 90 (96%) were found in the sample with the highest sequencing depth.

Discrepancies between the two replicates were observed for 22 (53.7%) of the 41 samples (Fig. 8). Ninety-four bacterial identifications, from now on called singletons, were made in only one replicate (Fig. 8 and Table S6). As expected, most singleton findings were found in the replicate with the highest sequencing depth (Fig. 8), and all singleton findings were among bacteria with either a low relative abundance or from samples with a low bacterial load (Table S6).

*(c) Microbial findings in bile samples.* When summarizing the microbial findings, we included all valid and likely valid identifications from both replicates. A summary of all bacterial findings, grouped at the genus level, is presented in Table 4.

**TABLE 4:** Identified bacteria by sequencing compared to conventional culture

Condition, parameter, and bacterial species^*a*^	Identifications by 16S rRNA sequencing		Growth by culture	
	Total no.	% of all microbial detections	No.	% of identified by 16S rRNA sequencing
Acute cholangitis (15 patients)				
Total no. of identifications	84		26	31
** Gram negative**	**27**	**32**	**9**	**33**
Klebsiella spp.	8	9.5	2	25
Escherichia coli	7	8.3	6	86
Campylobacter spp.	3	3.6		
Enterobacter spp.	2	2.4		
Haemophilus parainfluenzae	2	2.4		
* Aggregatibacter* spp.	2	2.4		
Hafnia alvei	1	1.2	1	100
Moraxella osloensis	1	1.2		
Serratia odorifera	1	1.2		
** Gram positive**	**42**	**50**	**15**	**35**
* Enterococcus* spp.	11	13.1	9	82
* *Streptococcus spp.	10	11.9	3	30
* Lactobacillus* spp.	6	7.1	1	17
* Actinomyces* spp.	4	4.8		
Granulicatella adiacens	2	2.4		
Rothia mucilaginosa	2	2.4		
Staphylococcus spp.	2	2.4	1	50
Abiotrophia defectiva	1	1.2		
Bacillus halodurans	1	1.2		
* Cellulosimicrobium* sp.	1	1.2	1	100
* Corynebacterium provencense*	1	1.2		
* Kocuria* sp.	1	1.2		
** Anaerobic**	**15**	**18**	**2**	**14**
* Fusobacterium* spp.	4	4.8		
* Veillonella* spp.	4	4.8		
Clostridium perfringens	4	3.6	2	67
Bifidobacterium dentium	1	1.2		
Cutibacterium avidum	1	1.2		
Finegoldia magna	1	1.2		
Intestinibacter bartlettii	1	1.2		

Noninfectious bile duct stenosis (26 patients)				
Total no. of identifications	215	40	19	
** Gram negative**	**44**	**21**	**16**	**36**
Escherichia spp.	7	3.3	6	86
Klebsiella spp.	7	3.3	5	71
Haemophilus spp.	6	2.8	1	17
Campylobacter spp.	3	1.4		
Enterobacter spp.	3	1.4		
* Neisseria* spp.	3	1.4		
* Citrobacter/Cronobacter*	2	0.9		
Pseudomonas aeruginosa	2	0.9		
* Aeromonas* sp.	1	0.5	2	200
* Bergeyella* sp. (HMT-322)	1	0.5		
Capnocytophaga gingivalis	1	0.5		
Citrobacter amalonaticus	1	0.5	1	100
Hafnia alvei	1	0.5		
* Hymenobacter* sp.	1	0.5		
Kluyvera ascorbata	1	0.5		
Pluralibacter gergoviae	1	0.5		
Proteus sp.	1	0.5		
* Pseudolabrys* sp.	1	0.5		
Serratia marcescens	1	0.5	1	100
** Gram positive**	**75**	**35**	**21**	**28**
Streptococcus spp.	35	16.3	8	23
* Actinomyces* spp.	13	6.0	2	15
* Enterococcus* spp.	7	3.3	6	86
Granulicatella adiacens	4	1.9		
Rothia mucilaginosa	4	1.9	2	50
* Saccharibacteria* (TM7) spp.	4	1.9		
Staphylococcus spp.	3	1.4	2	67
* Gemella* spp.	2	0.9		
* Corynebacterium* sp.	1	0.5		
Kocuria palustris	1	0.5		
Leuconostoc lactis	1	0.5	1	100
** Anaerobic**	**96**	**45**	**3**	**3**
* Veillonella* spp.	18	8.4		
* Prevotella* spp.	14	6.5	1	7
* Fusobacterium* spp.	7	3.3		
* Oribacterium* spp.	6	2.8		
* Leptotrichia* spp.	5	2.3		
* Clostridium* spp.	5	2.3	1	20
* Bifidobacterium* spp.	4	1.9		
Stomatobaculum longum	3	1.4		
* Peptostreptococcus* spp.	3	1.4		
Atopobium parvulum	2	0.9		
* Bacteroides* spp.	2	0.9	1	50
* Lachnoanaerobaculum* spp.	2	0.9		
Megasphaera micronuciformis	2	0.9		
Alloprevotella tannerae	1	0.5		
Alloscardovia omnicolens	1	0.5		
Anaerococcus vaginalis	1	0.5		
Bilophila wadsworthia	1	0.5		
Catabacter hongkongensis	1	0.5		
Catonella morbi	1	0.5		
* Colibacter massiliensis*	1	0.5		
Cryptobacterium curtum	1	0.5		
Dialister pneumosintes	1	0.5		
Eggerthella lenta	1	0.5		
Eubacterium sulci	1	0.5		
Finegoldia magna	1	0.5		
Fretibacterium fastidiosum	1	0.5		
* Lachnospiraceae* (G-2) sp.	1	0.5		
* Mogibacterium* sp.	1	0.5		
Parasutterella excrementihominis	1	0.5		
Parvimonas micra	1	0.5		
Porphyromonas pasteri	1	0.5		
* Selenomonas* sp.	1	0.5		
Slackia exigua	1	0.5		
Solobacterium moorei	1	0.5		
* Veillonellaceae* [G-1] sp.	1	0.5		

a Microbes that could be identified only to a species group or genus level are listed at the genus level. For microbes that could be identified to the species level, and where there were no other species identified within the same genus, the species name is listed.

All samples from patients with acute cholangitis contained bacteria as determined by both culture and sequencing (Table 1 and Fig. S1). For patients with noninfectious bile duct stenosis, 21 out of 26 (81%) samples contained bacteria as determined by sequencing. Among these, four samples were culture negative.

Compared to culture, sequencing found a much higher species richness in most samples (Tables 1 and 4). In the acute cholangitis group, 84 microbial identifications were made by sequencing whereof only 26 (30%) were cultured (Table 4). One identification, Granilucatella adiacens, was made solely by culture. In the group of noninfectious bile duct stone patients, 215 identifications were made by sequencing, whereof only 40 (19%) were cultured. Four unique identifications, one Staphylococcus epidermidis, one Staphylococcus warneri, one Corynebacterium pseudodiphtericum, and one Enterococcus faecalis were made by culture only.

## DISCUSSION

In this study, we investigate patterns of microbial contamination in targeted amplicon sequencing and their implications for postsequencing filtering of results. We demonstrate how the most dominant contaminant species can be used to establish sample-specific cutoffs for reliable identifications. We also show how sample bacterial load and sequencing depth affect sequencing results.

### Sequencing of negative controls does not reveal all contaminants.

Most current approaches for identifying and filtering contaminant bacteria rely on the assumption that sequencing of appropriate extraction controls will reveal the full spectrum of background contaminants that could possibly occur in the associated clinical samples (1–4). Our results contradict this assumption. We found that less than 10% of the contaminant species were detectable in all five replicates from the same negative control when split before the PCR amplification step (Fig. 2).

Recently, Erb-Downward et al. described the same PCR replicate variability (17). They suggested that the phenomenon occurred because of sequencing errors, possibly due to underloading of the flow cell and very low cluster densities. On the basis of data from both pre- and post-PCR replicates, we provide an alternative hypothesis, that the major contributor to the variation between pre-PCR replicates is the random inclusion of low abundance contaminant microbial DNA during pipetting of the PCR template. While a few contaminants, having a relatively higher concentration, will always be part of the PCR template, the majority of contaminants will be present at such low concentrations that they will only occasionally be included. They are under the law of small numbers (18), where a random sample is not likely to reflect the population from which it is drawn, and the similarity between different samples is low. This would explain why we robustly detect the most abundant contaminant taxa across all samples and extraction controls in a sequencing run, whereas the presence and identity of less abundant background contaminants vary from sample to sample (Fig. 3; see also Tables S1, S2, and S4 in the supplemental material). Further, the negative-control replicates that were split after the 16S PCR, i.e., after massive amplification of any low abundance target and therefore with expected low intersample variability, showed a very high homogeneity (Fig. 2 and Tables S1 and S2). The latter finding contradicts the hypothesis by Erb-Downward et al. (17). It is essential to acknowledge the difference between pre- and postamplification replicates, and only the latter is useful for addressing the reproducibility of the sequencing technology itself.

### Lower limit of detection.

When the DNA input of a given species in a sample is getting close to one copy per PCR, it will, like the low abundance contaminants, be under the law of small numbers. This thus constitutes a lower limit of detection for 16S deep sequencing as a method. Our sequencing of the mock community illustrates this point. From the 1:10^5^ dilution, we identified M. smithii in only 2 out of four PCR replicates. The theoretical abundance of M. smithii in the 1:10^5^ dilution was 67 cells per ml, corresponding to a little less than one copy in 2 μl PCR template (Table S3).

### Comparison of filtering methods.

The major strength of our method for filtering contaminants is its high specificity, found to be 97% when evaluated on mock community dilutions (Fig. 7). As expected, the use of a single negative and positive extraction control had a very low specificity (39%) (method 2; Fig. 7). The law of small numbers implies that increasing the number of extraction controls should provide a more complete description of the background contaminants, and including OTUs found in all 10 extraction controls (method 3; Fig. 7) did result in filtering of more true contaminants. However, many of the low abundance contaminants were still not flagged, and the specificity of this method remained low (64%).

Our findings might explain why promising postanalytic methods for removing contaminants, like the R-package Decontam, still display reduced specificity in low biomass/highly diluted samples (4, 5, 13). Decontam filtering of contaminant taxa had a specificity of 39% and 77%, respectively, in our mock community experiments (Fig. 7). The prevalence-based method in Decontam, which is recommended for low biomass samples (4), relies on the assumption that contaminating taxa are likely to have a higher prevalence in control samples than in true samples. Our results indicate that this assumption may be true only for the more abundant contaminant bacteria. Low abundance contaminant taxa that appear randomly in the negative extraction controls might not be recognized as contaminants.

The sensitivity of our suggested filtering method on the diluted mock communities was 83%. The mock community microbes wrongly classified as contaminants were those present in concentrations close to the absolute lower limit of detection, having a theoretical copy number ranging from <1 to 9 copies per 2 μl PCR template. Thus, this delineates the lower limit of detection for our filtering method. Many of the bile samples from the noninfectious patients also had low bacterial loads. They contained bacteria known to be part of the human oral microbiota, possibly reflecting contamination of the sampling catheter during the endoscopic retrograde cholangiopancreatography (ERCP) procedure. In some of these samples (e.g., samples 23 and 28 [see Fig. S1 in the supplemental material]), due to the low bacterial loads, many human oral bacteria were categorized as background contaminants by our filtering approach.

A major concern when subtracting all findings in the negative controls (1, 2, 13) is the situation where a species truly present in the sample is also found in the bacterial background. Our method allows for correct classification of these as relevant if they are represented by more reads than the most abundant contaminants. For the specific situation where the infection is caused by a species that is also among the dominant contaminants, one must look at alternative approaches. It is possible to calculate a sample-specific cutoff for differentiating between true and contaminant bacteria by using a combination of sequencing depth (number of reads) and the *C_T_* values of the sample and the corresponding negative control in the 16S rRNA PCR (Δ*C_T_*) (19). Although specific, this approach has a lower sensitivity.

Another suggested approach for contaminant filtering is to have an expert review of the samples and remove taxa that are considered biologically unexpected (1, 20). This method will however fall short if contaminant species are also biologically plausible, like many of the species identified in our extraction controls (e.g. *Anaerococcus* sp., *Actinomyces* sp., *Corynebacterium* sp., Cutibacterium acnes, Staphylococcus sp., Finegoldia magna, Haemophilus sp., Pseudomonas sp., *Prevotella* sp., Streptococcus sp., and *Moraxella* sp.) (Tables S2 and S4). However, combining our suggested filtering method with expert removal of biologically unexpected findings could possibly further increase the accuracy of results. In such a setting, clinically plausible findings below the cutoff of a valid identification could also be reported, but with more caution and as part of a broader clinical assessment.

Using the most abundant contaminant to establish a cutoff for likely valid identifications represents a dynamic approach taking into account both sequencing depth and the relative level of contamination in each individual sample. This is in contrast to some approaches based on a fixed cutoff, either a specified read count or a specified proportion of the total number of sequencing reads in each sample (21, 22). Such approaches will not be expedient for filtering samples with diverse bacterial loads or with dissimilar sequencing depths.

### Setting the lower cutoff for acceptable bacterial identifications.

We removed any species represented by less than 20% of the reads of the most abundant contaminant. This was a pragmatic cutoff, based on the observation that, with our reagents, inclusion of random background contaminants seemed to increase exponentially below this threshold (Fig. 4). However, as seen in Fig. 4, contaminants could occasionally occur at abundances up to 80% of the dominant background bacteria. We must therefore assume that some of the bacteria defined as “likely valid” in our clinical samples could represent contaminants. A likely example of this is the soil bacterium *Hymenobacter* sp., found in sample 41 with an abundance of 33% compared to the most abundant contaminant (Fig. S1).

The relative number of reads representing a given bacterium in a sample will fluctuate somewhat from sequencing run to sequencing run. Such variations will be more pronounced among low abundance bacteria since they, like the background contaminants, are more affected by random differences in the number of target DNAs pipetted for the amplification PCR. This is illustrated by the repeat sequencing of mock community dilutions, where the interreplicate variations in relative species abundances were higher in the most diluted samples (Fig. 5B). Low abundance species will therefore be vulnerable for accidentally falling below the cutoff in some runs, explaining why altogether 27 bacteria were validly detected in only one of the 16S rRNA replicates among the “low bacterial load” bile samples (Fig. 8).

### The relationship between bacterial load, sequencing depth, and diagnostic sensitivity.

Background contamination is described as mainly constituting a challenge in low biomass samples, and many studies report the inverse relationship between the bacterial load of a sample and the relative abundance of contaminating DNA (1–3, 5). We will argue that the absence of contaminant species in data from a high biomass sample is actually an indication of inadequate sequencing depth. If you are not seeing any contaminants, there may remain undiscovered species with lower abundances that you could have detected using a higher number of reads (as in our sequencing of the 1:10 dilution of the mock community). This is well exemplified by our repeated 16S rRNA sequencing of clinical samples, where all except 1 out of 62 singleton findings were made in the replicate with the highest sequencing depth (Fig. 8). All these extra identifications were also, as expected, among the low abundance species in their samples with relative abundances of <1% of the total number of accepted bacterial reads (Fig. S1). Sample 38 (moderate bacterial load, *C_T_* value of 22.5) represented the most extreme example (Fig. S1). For this sample, the number of accepted reads increased from 17,966 reads in the first replicate to 188,744 in the second. With this increase, we were able to identify 32 additional species and, as an indication of sufficient depth, small amounts of contamination (113 reads/0.001% with *Ralstonia picketti*). The high number of reads needed for robust description of polymicrobial clinical infections is emphasized by our data. For samples with moderate to high bacterial loads, even a sequencing depth of hundreds of thousands reads was frequently insufficient to start seeing contaminant bacteria (Table 2 and Fig. S1).

### Cross-contamination.

Another possible source of contamination in target amplicon sequencing is cross-contamination between samples (2). The level of cross-contamination is difficult to determine with certainty. To minimize the risk of sequencing noise and cross-contamination disturbing our results, we rejected all OTU clusters containing less than 50 sequences. This is a similar or even more strict criterion than other studies have used (9, 19, 22–24).

### Limitations.

We believe the general principles outlined in the study will be transferable to other clinical labs. However, background contamination will vary between labs, between extraction kits and PCR reagents, and even between batches of the same extraction kits and PCR reagents (2). Every lab should analyze and monitor the pattern of contamination in their own sequencing results if adopting our approach for filtering of contaminants and adjust their filtering cutoffs according to their findings. Adjustments could include, e.g., the number of “top contaminants” or the “frequency threshold rate.”

### Conclusion.

In this study, we demonstrate the limitations of simply using microbial identifications in negative controls as the basis for filtering background bacterial contamination. The main concern regarding this strategy until now has been that the negative controls may contain bacteria that are also truly present in the clinical samples or that the negative controls may be contaminated with DNA from the clinical samples during the sequencing process (1, 2, 13) and that true findings therefore will be discarded as contaminants. We demonstrate that due to the law of small numbers, the risk of accepting contaminants as true findings should be of equal concern using this strategy.

We suggest using the most abundant background contaminant species to define a level in each sample from where identifications might represent contamination. Below this level, again due to the law of small numbers, it rapidly becomes very demanding to discriminate between background and true findings. The most abundant contaminant DNA can also serve to evaluate sequencing depth. Adequate sequencing depth can be claimed only when the analysis also picks up background contamination.

## MATERIALS AND METHODS

### Inclusion of patients and collection of bile samples.

This was a prospective, single-center study performed at Haukeland University Hospital, Bergen, Norway. The study was approved by the regional ethical committee (2015/65). Written informed consent was obtained from all participants.

From July 2015 to April 2017, bile samples were collected from all patients undergoing endoscopic retrograde cholangiopancreatography (ERCP). Patients diagnosed with either acute calculous cholangitis, defined according to the Tokyo Guidelines 2013 (25) (TG13) criteria for a definite diagnosis or noninfectious bile duct stone were included for further analysis.

Bile samples were immediately placed in sterile sample glass and sent to the laboratory for analysis after sampling. Upon arrival to the laboratory, DNA was extracted directly from 400 μl of bile as described previously (15, 16, 26). The eluate was stored at −80°C for later deep sequencing analysis. All samples were also routinely cultured according to our previously described laboratory guidelines (16).

### Endoscopic retrograde cholangiography and pancreatography procedure.

The intestine was rinsed with a solution of water and Minifom before procedure. ERCP was performed with the patient in the supine position. The patient was sedated with midazolam and pethidine, and if needed supplemented with buscopan for bowel relaxation. A side-viewing, sterilized, endoscope (Evis Exera III Duodenovideoscope, Olympus TJF – Q190V, Olympus) was used. Wire guided selective bile duct cannulation was performed with use of a guidewire (Dreamwire 0.035 in., 260 cm; Boston Scientific, Costa Rica) passed through a sterile sphincterotome catheter (Jagtome RX 44; Boston Scientific, Costa Rica). The position in the bile duct was confirmed by X-ray to identify the position of the catheter and guide wire before aspiration of approximately 2 to 5 ml bile. If there was any concern about the location of the guidewire, the sphincterotome was gently advanced over the guidewire, and a small amount of contrast was injected to delineate the anatomy. If there were any difficulties with cannulation of the ampulla of Vater, normal saline was injected to dilate the bile duct. Normal saline injections was also used to flush the bile ducts if bile aspiration attempts yielded little or no fluid in return on the catheter.

### Mock community dilution.

A staggered mock community from ZymoBIOMIC were used (ZymoBIOMICS Gut Microbiome Standard, catalog no. D6331; Zymo Research Corp., Irvine, CA, USA). This mock community consists of 19 bacterial strains representing 15 bacterial species (Faecalibacterium prausnitzii, Veillonella rogosae, Roseburia hominis, Bacteroides fragilis, Prevotella corporis, Bifidobacterium adolescentis, Fusobacterium nucleatum, Lactobacillus fermentum, Clostridioides difficile, Akkermansia muciniphila, Methanobrevibacter smithii, Salmonella enterica, Enterococcus faecalis, Clostridium perfringens, and Escherichia coli strains JM109, B-3008, B-2207, B-766, and B-1109) and two fungal species (Saccharomyces cerevisiae and Candida albicans). The mock community was diluted with microbial DNA-free water (Qiagen) in seven rounds of a serial 10-fold dilution prior to DNA extraction. The dilutions were analyzed with a SYBR green real-time 16S rRNA PCR using a previously described protocol (15) to obtain a semiquantitative measure of the bacterial load of each dilution. A dilution with high bacterial load (1:10) and two different dilutions with low bacterial load (1:10^5^ and 1:10^6^) were selected for further analysis. Negative and positive extraction controls were included and followed all processing steps.

### Gene targets.

In all bile samples, mock community samples, and extraction control samples, the 16S rRNA gene V3-V4 region was sequenced (see Table S8 in the supplemental material). For selected bile samples, a part of the *rpoB* gene were also sequenced in a separate sequencing run to obtain a higher taxonomic resolution for *Enterobacteriaceae*, *Enterococcus*, Streptococcus, and Staphylococcus species identified by the 16S rRNA sequencing (16). Species identified at a higher taxonomic level with partial *rpoB* gene sequencing compared to partial 16S rRNA gene sequencing (V3-V4) are listed in Table S7. All primers used were the same as described previously (16), except for a modification of one of the two forward *RpoB*_ESS primers to obtain better coverage of Enterococcus raffinosus (Table S8). All primers are listed in Table S8.

10.1128/mBio.00598-21.7 TABLE S7 Species identified at a higher taxonomic level with use of the partial *rpoB* gene compared to partial 16S rRNA gene sequencing (V3-V4). Download Table S7, PDF file, 0.1 MB. Copyright © 2021 Dyrhovden et al. 2021 Dyrhovden et al. https://creativecommons.org/licenses/by/4.0/ This content is distributed under the terms of the Creative Commons Attribution 4.0 International license.

10.1128/mBio.00598-21.8 TABLE S8 (A) Primers with adapter sequences. (B) PCR mixture for the different gene targets and the temperature profile of the amplicon PCR. Download Table S8, PDF file, 0.2 MB. Copyright © 2021 Dyrhovden et al. 2021 Dyrhovden et al. https://creativecommons.org/licenses/by/4.0/ This content is distributed under the terms of the Creative Commons Attribution 4.0 International license.

### Sequencing procedure.

The Illumina Miseq system (Illumina, Redwood City, CA) was used for sequencing. The sequencing protocol was a modified version of the of the Illumina 16S Metagenomic Library Preparation protocol (27) as described previously (15, 16). Briefly, the sequencing workflow included the following stages. The target genes were amplified in an amplicon PCR using the same temperature profile for all targets. An overview of the PCR mixture for the different gene targets and the temperature profile of the amplicon PCR is provided in Table S8. After PCR cleanup of the amplicon PCR product with use of AMPure XP beads, the next step was attachment of dual indices and Illumina sequencing adapters in an index PCR. The index PCR product underwent a similar cleanup, followed by a fluorometric quantification of the DNA content of each sample using Qubit 3.0 fluorometer (Fisher Scientific) and the QubitR dsDNA (double-stranded DNA) HS (high-sensitivity) assay kit (0.2 to 100 ng). Samples were then diluted using 10 mM Tris (pH 8.5) to reach a final concentration of 4 nM, before they were pooled together into a final library pool that was denatured, diluted, and mixed with a Phix control before loaded on the Miseq system as described in the Illumina protocol (27).

For the 16S rRNA amplicon sequencing of bile samples, each sample was split into two replicates (16S rRNA replicate 1 and 16S rRNA replicate 2) after DNA extraction and then processed in different PCR amplification and sequencing runs. The second replicate from each sample was sequenced with fewer samples per sequencing run to obtain a higher sequencing depth.

### Assessing the bacterial load in the bile samples.

A semiquantitative measure of bacterial load in each sample was calculated using the *C_T_* value from the SYBR green real-time 16S rRNA PCR, following the same protocol as for the mock community experiment. According to their *C_T_* value, samples were categorized as having either high bacterial load (*C_T_* values ranging from lowest to 19), moderate bacterial load (*C_T_* values ranging from 20 to 24) or low bacterial load (*C_T_* values ranging from 25 to highest).

### Extraction controls.

Each sample was processed together with a parallel negative extraction control consisting of lysis buffer and PCR-grade water. For the bile samples, all negative extraction controls were mixed into two or three pools before sequencing, depending on the number of samples included in the sequencing run. In addition, a weakly positive extraction control consisting of Legionella pneumophila suspended in PCR-grade water was included.

### Postsequencing processing.

The Miseq Reporter software was used for removing primers, demultiplexing, and generating FASTQ files for each sample. AdapterRemoval 2.2.2 (28) was used for trimming adapter sequences and low-quality bases and to merge the forward and reverse FASTQ files of each sample, using the following command: AdapterRemoval –file1 <reads_1.fq> --file2 <reads_2.fq> --basename <mymergedfile> --threads 7 –trimns –trimqualities –minquality 20 –collapse – adapter-list <adapters>.txt –gzip.

Downstream analysis was then performed using the RipSeq next-generation sequencing (NGS) software (Pathogenomix, Santa Cruz, CA) (15, 16) (*de novo* clustering into operational taxonomic units [OTUs] using a 99% similarity threshold). A chimera check was performed with the RipSeq online tool.

### Taxonomic assignment.

OTUs were assigned using the RipSeq online BLAST search against the RipSeqs curated database “Pathogenomics Prime 16S” (16S), “*Pathogenomix rpoB_ESS*,” ”*Pathogenomix rpoB_Ent*,” and “*GenBank Bacteria 1 – All bacterial targets*, *Valid Species and Pubmed*” (*rpoB*). OTUs that did not match a reference sequence using these RipSeq curated databases were manually assigned by performing a BLAST search against the GenBank NCBI database and the Human Oral Microbiome Database (www.homd.org). OTUs mapping to the same reference species were merged.

Criteria used for taxonomy assignments for both 16S rRNA and *rpoB* gene were the same as described previously (16) (for 16S rRNA species-level identification, ≥99.3% homology with a high-quality reference, and minimum distance >0.7% to the next alternative species). OTUs obtaining species-level homology but with an insufficient distance to the next species were assigned to a species group or listed as a slashed result. OTUs that did not assign to any known species were indicated as “Unknown bacteria #.” A full list of all species groups and of the best BLAST search match in GenBank NCBI database for all OTUs termed as “Unknown bacteria #” is found in Table S9.

10.1128/mBio.00598-21.9 TABLE S9 Species included in species groups listed in tables and figures (A) and best BLAST search match for “Unknown bacteria” listed in tables and figures (B). Download Table S9, PDF file, 0.2 MB. Copyright © 2021 Dyrhovden et al. 2021 Dyrhovden et al. https://creativecommons.org/licenses/by/4.0/ This content is distributed under the terms of the Creative Commons Attribution 4.0 International license.

### Secondary filtration of sequencing results.

A lower cutoff for the number of representative sequences required to retain an OTU is recommended as a secondary filtration to diminish problems related to sequencing noise and cross-contamination of samples (9, 19, 22–24, 29). We rejected OTUs represented by fewer than 50 reads. Further filtering of bacterial background DNA from the sequencing results is outlined in Results.

### Statistical analysis.

Statistical analyses were performed using SPSS 25 (IBM Corp.) and the R programming language (30). Clinical and microbial characteristics of categorical and continuous data were analyzed with Pearson’s chi-squared test and Student’s *t* test, respectively. Mann-Whitney U-test was used for continuous, skewed variables. Figures illustrating microbial distribution were produced using the R-packages “VennDiagram” (31) version 1.6.0 and “ggplot2” (32) version 3.2.1. Diversity analyses were performed using the R-package “phyloseq” (33) version 1.30.0. Rarefaction of data used in diversity measures was performed using the phyloseq package in R with the following arguments: rarefy_even_depth(Otu_table, sample.size = min(sample_sums(Otu_table)), rngseed = TRUE, replace = TRUE, verbose = TRUE).

### Data availability.

The source data from experiment 1 and experiment 2 have been deposited in the European Nucleotide Archive (ENA) at EMBL-EBI under accession number PRJEB44556 (https://www.ebi.ac.uk/ena/browser/view/PRJEB44556).

Other source data of this study are available from the corresponding author upon request. Not all patient data are publicly available due to restrictions from the Regional Ethical Committee.
