# Supplementary material for: Managing Contamination and Diverse Bacterial Loads in 16S rRNA Deep Sequencing of Clinical Samples: Implications of the Law of Small Numbers
Source: mBio. 2021 Jun 8;12(3):e00598-21. doi: 10.1128/mBio.00598-21 (PMC8262989; doi:10.1128/mBio.00598-21)
Supplement: TABLE S1 [file mbio.00598-21-st001.pdf]

Supplementary Table S1

A) Characteristics and beta-diversity measures of the extraction control replicates in Experiment 1.

	Negative extraction control 1		
	PCR replicates	Sequencing replicates	p-value[a]
Number of samples	5	5	
Accepted reads per sample[b], mean	278,466	322,314	
min-max	227,539-319871	303,225-336,425	
Bacterial identifications per sample (ranged min to max)	14, 16, 19, 24, 25	14, 14, 14, 14, 14	
Jaccard distance[c], mean	0.73	0.00	<0,001
min-max	0.68-0.78		
Bray Curtis dissimilarity[c], mean	0.49	0.04	<0,001
min-max	0.37-0.57	0.01-0.07	

	Negative extraction control 2		
	PCR replicates	Sequencing replicates	p-value[a]
Number of samples	5	5	
Accepted reads per sample[b], mean	290,391	299,105	
min-max	206,617-352,522	227,323-357,574	
Bacterial identifications per sample (ranged min to max)	12, 17, 18, 21, 24	24, 24, 24, 24, 24	
Jaccard distance[c], mean	0.77	0.00	<0,001
min-max	0.65-0.85		
Bray Curtis dissimilarity[c], mean	0.44	0.04	<0,001
min-max	0.36-0.53	0.01-0.07	

	Positive extraction control		
	PCR replicates	Sequencing replicates	p-value[a]
Number of samples	5	4	
Accepted reads per sample[b], mean	313,545	309,860	
min-max	222,116-395,935	256,164-395,935	
Bacterial identifications per sample (ranged min to max)	11, 19, 20, 24, 25	18, 19, 19, 19	
Jaccard distance[c], mean	0.73	0.03	<0,001
min-max	0.65-0.81	0.00-0.05	
Bray Curtis dissimilarity[c], mean	0.44	0.04	<0,001
min-max	0.37-0.51	0.02-0.06	

[a] Students t-test for continuous, normal distributed variables. Mann-Whitney U-test for continuous, skewed variables.

[b] Number of reads per sample after removal of short reads (< 250 base pairs), small clusters (< 50 reads), human reads and chimeras.

[c] Data rarified as described in materials and method

B) Number of accepted reads and alpha-diversity measures for all extraction control replicates in Experiment 1.

	Total number of reads[a]	Accepted reads[b] (n)	Bacterial identifications (n)	Shannon Index[a]	InvSimpson index[a]	Fisher's alpha index[c]
**Negative extraction control 1**						
PCR replicates						
1	583348	319871	14	1,98	5,23	1,15
2	547193	271946	19	2,42	7,93	1,60
3	470255	227539	16	2,41	8,87	1,33
4	547230	262352	24	2,64	9,87	2,07
5	637553	310622	25	2,73	10,60	2,16
Sequencing replicates						
1	583348	319871	14	1,98	5,23	1,12
2	573792	324402	14	1,93	4,88	1,12
3	538117	303646	14	1,95	5,01	1,12
4	552688	303225	14	2,01	5,30	1,12
5	651891	360425	14	1,96	5,11	1,12
**Negative extraction control 2**						
PCR replicates						
1	686972	338240	24	2,55	7,81	2,09
2	633095	331274	17	2,12	5,52	1,43
3	432850	206617	21	2,40	7,00	1,80
4	444629	223301	18	2,31	7,77	1,52
5	655334	352522	12	2,11	6,31	0,98
Sequencing replicates						
1	686972	338240	24	2,55	7,83	2,07
2	601795	305697	24	2,48	6,91	2,07
3	553463	266693	24	2,55	7,80	2,07
4	722975	357574	24	2,62	9,04	2,07
5	474732	227323	24	2,58	8,41	2,07

**Positive extraction control**							
PCR replicates							
	1	797249	395935	19	2,28	7,67	1,61
	2	573511	285593	11	1,94	5,83	0,88
	3	737625	352059	24	2,57	8,23	2,07
	4	648329	312020	20	2,69	10,96	1,70
	5	461088	222116	25	2,52	7,42	2,17
Sequencing replicates							
	1	797249	395935	19	2,28	7,66	1,58
	2	530500	256164	19	2,36	8,35	1,58
	3	555651	275015	18	2,25	7,40	1,49
	4	629303	312325	19	2,33	7,85	1,58

[a] Total number of reads: Number of reads per sample before post-sequencing data processing

[b] Accepted reads: Number of reads per sample for after removal of short reads (< 250 base pairs), small clusters (< 50 reads), human reads and chimeras.

[c] Data rarified as described in materials and methods.

C) Sample-to-sample beta diversity measures for all extraction control replicates in Experiment 1.

Negative extraction control 1 - PCR replicates

Jaccard_distance	replicate 1	replicate 2	replicate 3	replicate 4
replicate 2	0,68			
replicate 3	0,75	0,75		
replicate 4	0,73	0,74	0,75	
replicate 5	0,78	0,74	0,68	0,71

Bray_curtis_dissimilarity	replicate 1	replicate 2	replicate 3	replicate 4
replicate 2	0,37			
replicate 3	0,48	0,48		
replicate 4	0,49	0,47	0,54	
replicate 5	0,57	0,48	0,51	0,52

Negative extraction control 1 - Sequencing replicates

Jaccard_distance	replicate 1	replicate 2	replicate 3	replicate 4
replicate 2	0,00			
replicate 3	0,00	0,00		
replicate 4	0,00	0,00	0,00	
replicate 5	0,00	0,00	0,00	0,00

Bray_curtis_distance	replicate 1	replicate 2	replicate 3	replicate 4
replicate 2	0,03			
replicate 3	0,02	0,01		
replicate 4	0,06	0,07	0,06	
replicate 5	0,01	0,02	0,01	0,07

Negative extraction control 2 - PCR replicates

Jaccard_distance	replicate 1	replicate 2	replicate 3	replicate 4
replicate 2	0,79			
replicate 3	0,71	0,73		
replicate 4	0,83	0,65	0,78	
replicate 5	0,76	0,79	0,78	0,85

Bray_curtis_distance	replicate 1	replicate 2	replicate 3	replicate 4
replicate 2	0,45			
replicate 3	0,38	0,38		
replicate 4	0,53	0,41	0,52	
replicate 5	0,36	0,40	0,42	0,51

Negative extraction control 2 - Sequencing replicates

Jaccard_distance				
	replicate 1	replicate 2	replicate 3	replicate 4
replicate 2	0,00			
replicate 3	0,00	0,00		
replicate 4	0,00	0,00	0,00	
replicate 5	0,00	0,00	0,00	0,00

Bray_curtis_distance				
	replicate 1	replicate 2	replicate 3	replicate 4
replicate 2	0,04			
replicate 3	0,01	0,03		
replicate 4	0,04	0,07	0,04	
replicate 5	0,03	0,06	0,04	0,03

Positive extraction control - PCR replicates

Jaccard_distance				
	replicate 1	replicate 2	replicate 3	replicate 4
replicate 2	0,70			
replicate 3	0,81	0,79		
replicate 4	0,74	0,65	0,71	
replicate 5	0,78	0,76	0,68	0,68

Bray_curtis_distance				
	replicate 1	replicate 2	replicate 3	replicate 4
replicate 2	0,43			
replicate 3	0,45	0,45		
replicate 4	0,51	0,40	0,46	
replicate 5	0,46	0,47	0,37	0,40

Positive extraction control - Sequencing replicates

Jaccard_distance			
	replicate 1	replicate 2	replicate 3
replicate 2	0,00		
replicate 3	0,05	0,05	
replicate 4	0,00	0,00	0,05

Bray_curtis_distance			
	replicate 1	replicate 2	replicate 3
replicate 2	0,05		
replicate 3	0,02	0,06	
replicate 4	0,05	0,04	0,04

[a] Data rarified as described in materials and methods.
