# Supplementary material for: Managing Contamination and Diverse Bacterial Loads in 16S rRNA Deep Sequencing of Clinical Samples: Implications of the Law of Small Numbers
Source: mBio. 2021 Jun 8;12(3):e00598-21. doi: 10.1128/mBio.00598-21 (PMC8262989; doi:10.1128/mBio.00598-21)
Supplement: TABLE S3 [file mbio.00598-21-st003.pdf]

Supplementary Table S3: Theoretical microbial composition and theoretical abundance of each bacteria for different mock community dilutions used in Experiment 2

A) Undiluted mock community. Data from producer of the staggered mock community.

	Theoretical composition (%)			Theoretical abundance of each microbe
**Species**	**Genomic DNA**	**16S Only**	**Cell Number**	**Cell (n) per ml**
Faecalibacterium prausnitzii	14	17,63	14,82	583908000
Veillonella rogosae	14	15,87	20,01	788394000
Roseburia hominis	14	9,89	12,47	491318000
Bacteroides fragilis	14	9,94	8,36	329384000
Prevotella corporis	6	4,98	6,28	247432000
Bifidobacterium adolescentis	6	8,78	8,86	349084000
Fusobacterium nucleatum	6	7,49	7,56	297864000
Lactobacillus fermentum	6	9,63	9,71	382574000
Clostridioides difficile	1,5	2,62	1,10	43340000
Akkermansia muciniphila	1,5	0,97	1,62	63828000
Methanobrevibacter smithii	0,1	0,066	0,17	6698000
Salmonella enterica	0,01	0,009	0,01	256100
Enterococcus faecalis	0,001	0,0009	0,00	43340
Clostridium perfringens	0,0001	0,0002	0,00	3546
Escherichia coli	14	12,12	8,73	343962000
Saccharomyces cerevisiae	1,4	N/A	0,16	6304000
Candida albicans	1,5	N/A	0,16	6304000
Sum	100		100	3940696986

B) 1:10 dilution of mock community

	Theoretical abundance of each bacteria			
**Species**	**Cell (n) per ml**	**Cell (n) input in DNA extraction:**	**16S copies in 100 µl extraction eluate**	**16S copies in 2 µl extraction eluate**
Faecalibacterium prausnitzii	58390800	14597700	87586200	1751724
Veillonella rogosae	78839400	19709850	78839400	1576788
Roseburia hominis	49131800	12282950	49131800	982636
Bacteroides fragilis	32938400	8234600	49407600	988152
Prevotella corporis	24743200	6185800	24743200	494864
Bifidobacterium adolescentis	34908400	8727100	43635500	872710
Fusobacterium nucleatum	29786400	7446600	37233000	744660
Lactobacillus fermentum	38257400	9564350	47821750	956435
Clostridioides difficile	4334000	1083500	13002000	260040
Akkermansia muciniphila	6382800	1595700	4787100	95742
Methanobrevibacter smithii	669800	167450	334900	6698
Salmonella enterica	25610	6403	44818	896
Enterococcus faecalis	4334	1084	4334	87
Clostridium perfringens	355	89	887	18

Escherichia coli	34396200	8599050	60193350	1203867
Sum	392808899	98202225	496765838	9935317

C) 1:10^5 dilution of mock community

	**Theoretical abundance of each bacteria**			
**Species**	**Cell (n) per ml**	**Cell input in DNA extration:**	**16S copies in 100 µl extraction eulate**	**16S copies in 2 µl extraction eluate**
Faecalibacterium prausnitzii	5839	1460	8759	175
Veillonella rogosae	7884	1971	7884	158
Roseburia hominis	4913	1228	4913	98
Bacteroides fragilis	3294	823	4941	99
Prevotella corporis	2474	619	2474	49
Bifidobacterium adolescentis	3491	873	4364	87
Fusobacterium nucleatum	2979	745	3723	74
Lactobacillus fermentum	3826	956	4782	96
Clostridioides difficile	433	108	1300	26
Akkermansia muciniphila	638	160	479	10
Methanobrevibacter smithii	67	17	33	0,7
Salmonella enterica	2,6	0,6	4,5	0,1
Enterococcus faecalis	0	0	0	0,0
Clostridium perfringens	0	0	0	0,0
Escherichia coli	3440	860	6019	120
Sum	39281	9820	49677	994

D) 1:10^6 dilution of mock community

	**Theoretical abundance of each bacteria**			
**Species**	**Cell (n) per ml**	**Cell input in DNA extration:**	**16S copies in 100 µl extraction eulate**	**16S copies in 2 µl extraction eluate**
Faecalibacterium prausnitzii	584	146	876	17,5
Veillonella rogosae	788	197	788	15,8
Roseburia hominis	491	123	491	9,8
Bacteroides fragilis	329	82	494	9,9
Prevotella corporis	247	62	247	4,9
Bifidobacterium adolescentis	349	87	436	8,7
Fusobacterium nucleatum	298	74	372	7,4
Lactobacillus fermentum	383	96	478	9,6
Clostridioides difficile	43	11	130	2,6
Akkermansia muciniphila	64	16	48	1,0
Methanobrevibacter smithii	7	2	3	0,1
Salmonella enterica	0	0	0	0,0
Enterococcus faecalis	0	0	0	0,0
Clostridium perfringens	0	0	0	0,0

Escherichia coli	344	86	602	12,0
Sum	3928	982	4968	99
