# Supplementary material for: Managing Contamination and Diverse Bacterial Loads in 16S rRNA Deep Sequencing of Clinical Samples: Implications of the Law of Small Numbers
Source: mBio. 2021 Jun 8;12(3):e00598-21. doi: 10.1128/mBio.00598-21 (PMC8262989; doi:10.1128/mBio.00598-21)

Supplementary Figure S1:

Barchart illustrating the sequencing result of each bile sample in 16S rRNA replicate 2. Number of reads per OTU are given on a log-transformed scale. Bars for species with an abundance above the top abundant contaminant is categorized as "Valid" and colored dark blue. Bars for species with an abundance between 20 to 100% of the top abundant contaminant is categorized as "Likely valid" and colored light blue. Bars with an abundance below 20% of the top abundant contaminant is categorized as "Contaminant" and colored red.

- S Also identified in corresponding 16SrRNA sequencing replicate
- R Also identified by rpoB sequencing
- C Also identified by culture
- N Also identified in extraction control

Sample 01-15: Bile samples from patients with acute cholangitis.

Sample 01

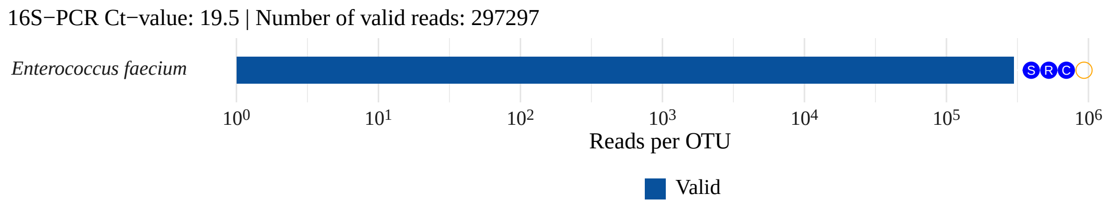

Sample 02

16S−PCR Ct−value: 12.5 | Number of valid reads: 63567

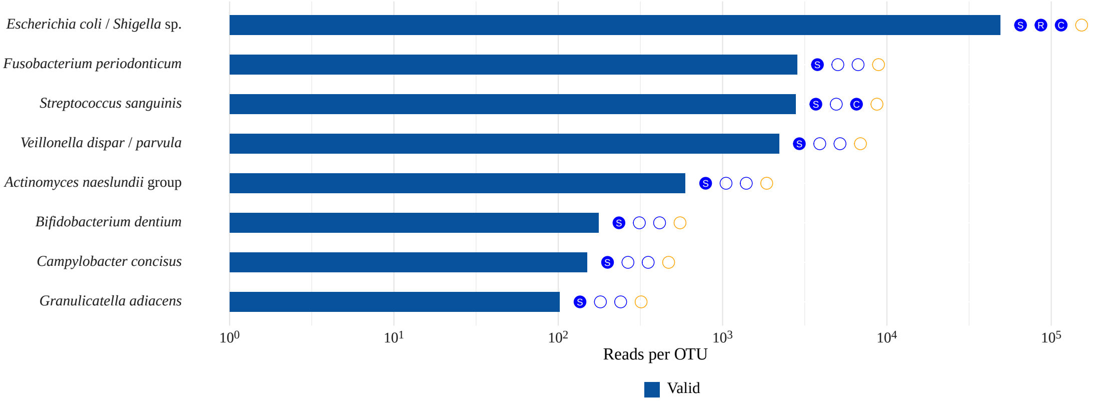

Sample 03

16S−PCR Ct−value: 16.3 | Number of valid reads: 182375

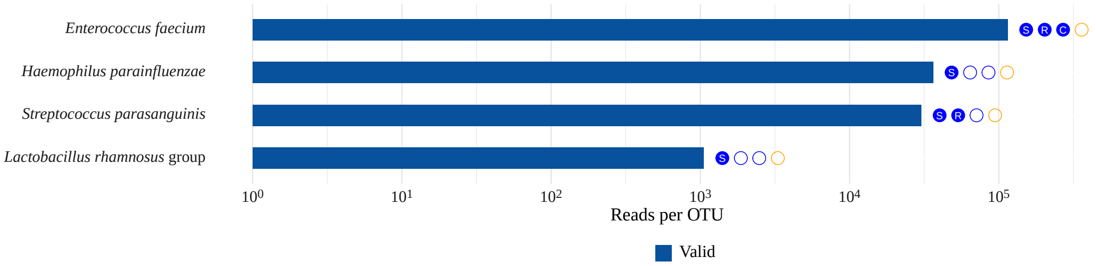

Sample 04

16S−PCR Ct−value: 12.9 | Number of valid reads: 235807

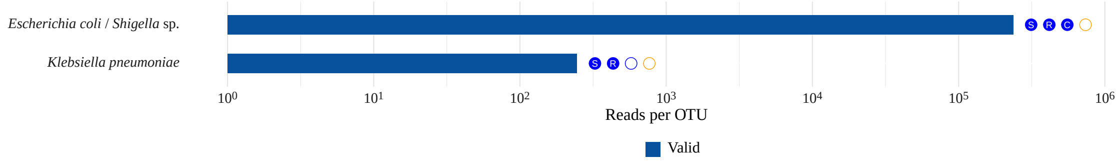

Sample 05

16S−PCR Ct−value: 17.6 | Number of valid reads: 161737

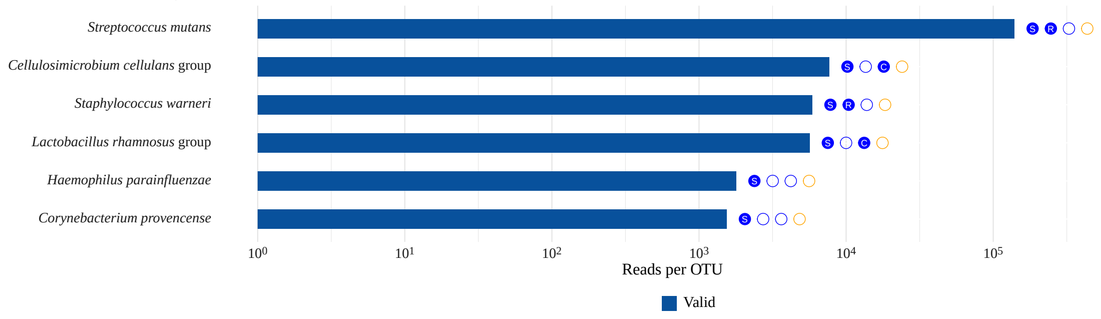

Sample 06

16S−PCR Ct−value: 27.9 | Number of valid reads: 60399

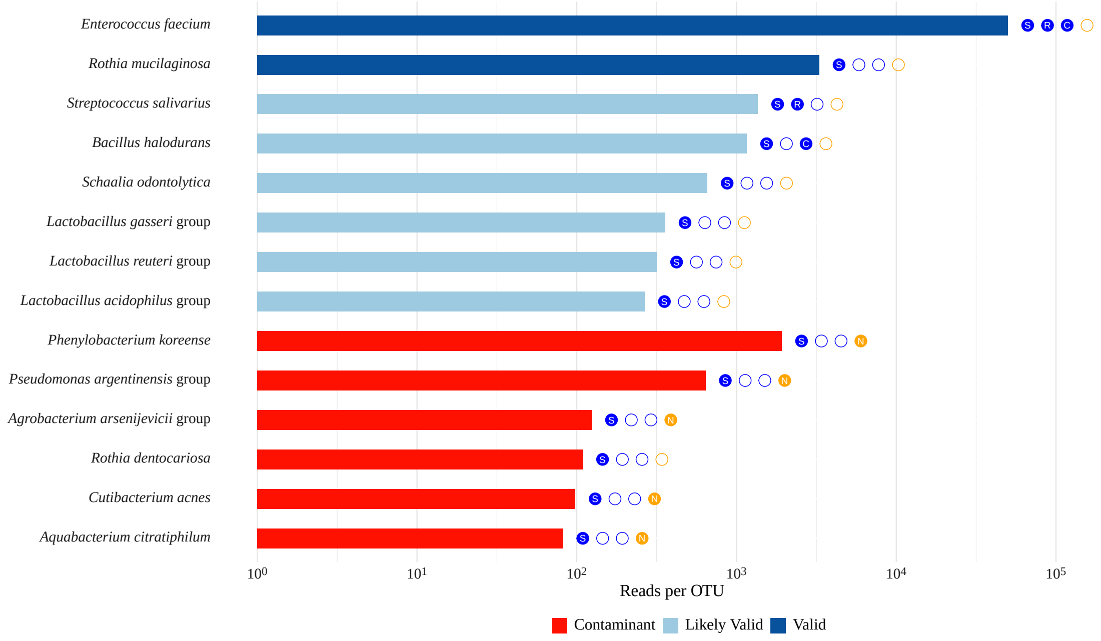

Sample 07

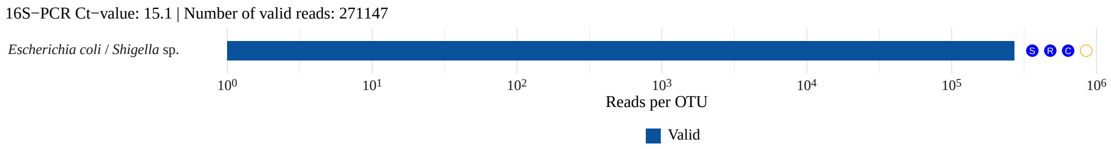
16S−PCR Ct−value: 15.1 | Number of valid reads: 271147
Escherichia coli / Shigella sp.
S
R
C
10^0
10^1
10^2
10^3
10^4
10^5
10^6
Reads per OTU
Valid

Sample 08

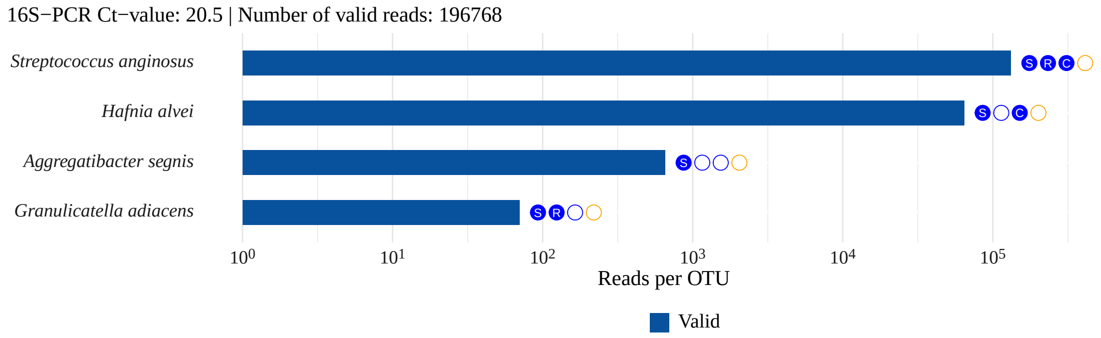

16S−PCR Ct−value: 20.5 | Number of valid reads: 196768
Streptococcus anginosus
Hafnia alvei
Aggregatibacter segnis
Granulicatella adiacens
S R C
S C
S
S R
10^0
10^1
10^2
10^3
10^4
10^5
Reads per OTU
Valid

Sample 09

16S−PCR Ct−value: 26.7 | Number of valid reads: 259551

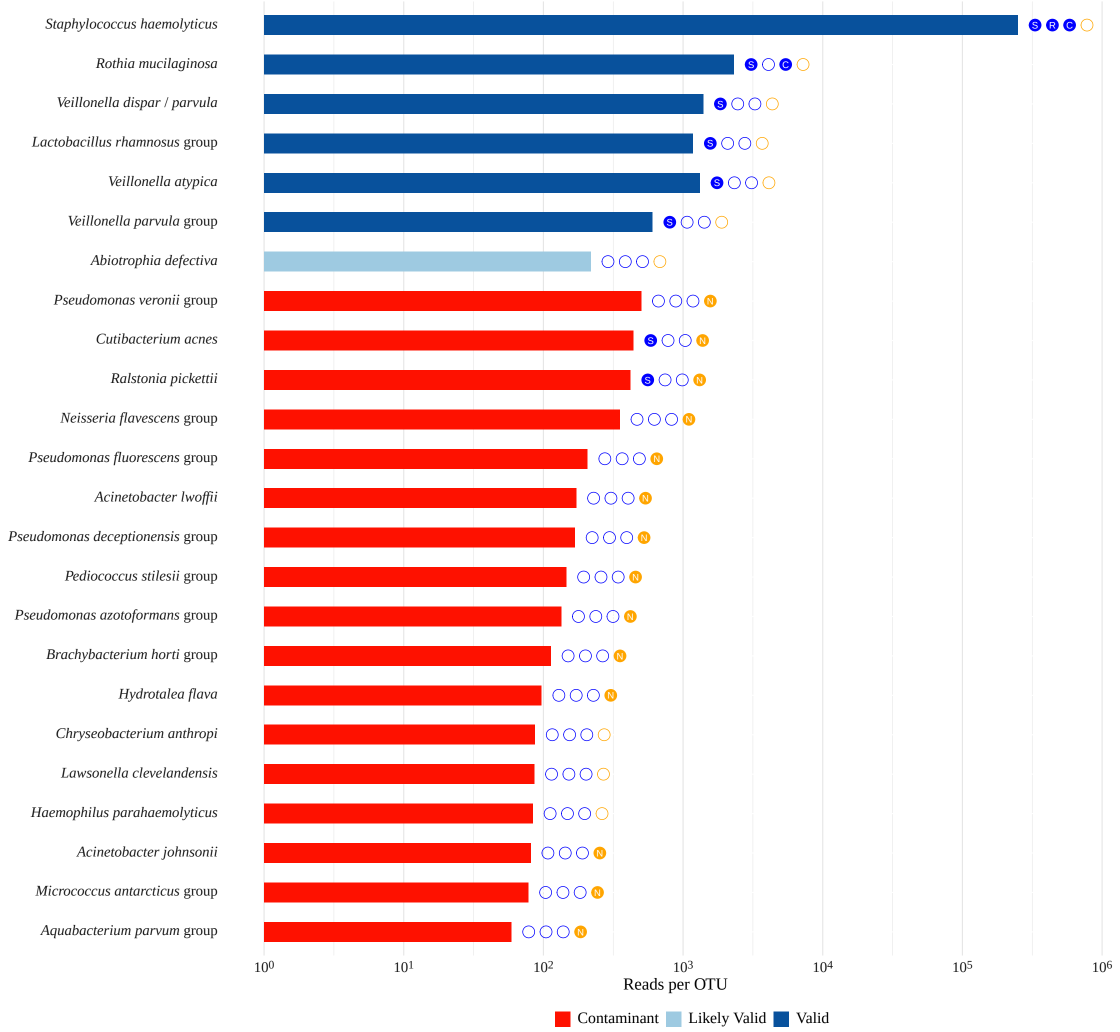

Sample 10

16S−PCR Ct−value: 22.6 | Number of valid reads: 166919

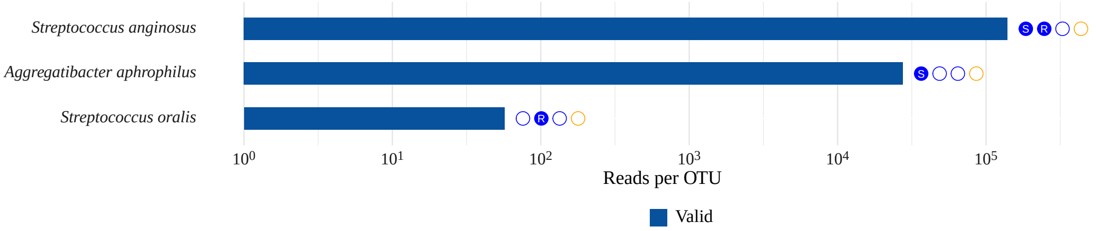

Sample 11

16S−PCR Ct−value: 24.4 | Number of valid reads: 429752

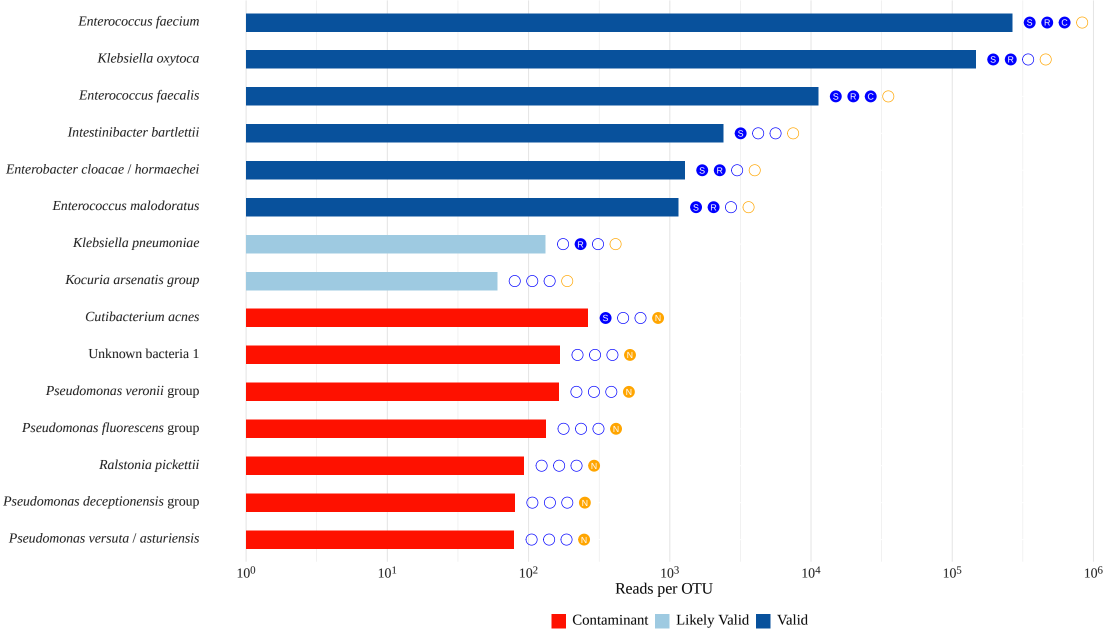

Sample 12

16S−PCR Ct−value: 18.5 | Number of valid reads: 332011

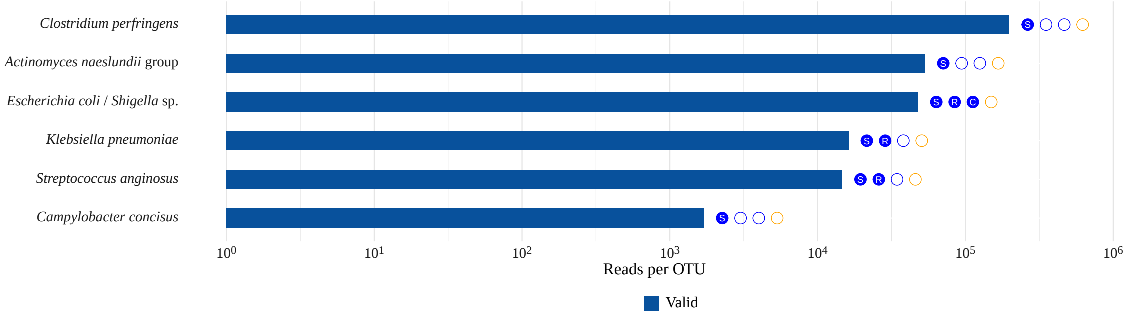

Sample 13

16S−PCR Ct−value: 19.2 | Number of valid reads: 376639

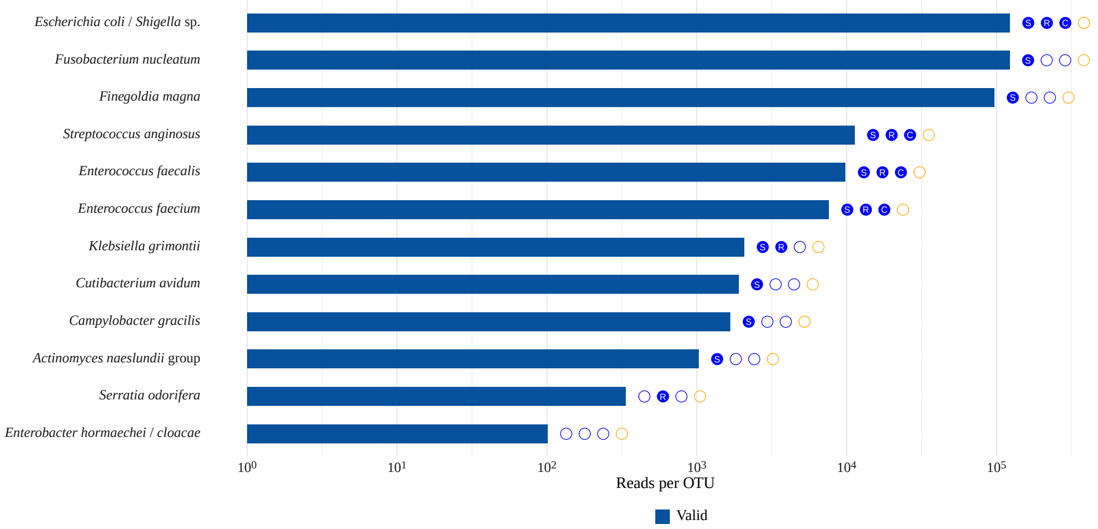

Sample 14

16S−PCR Ct−value: 15.2 | Number of valid reads: 317604

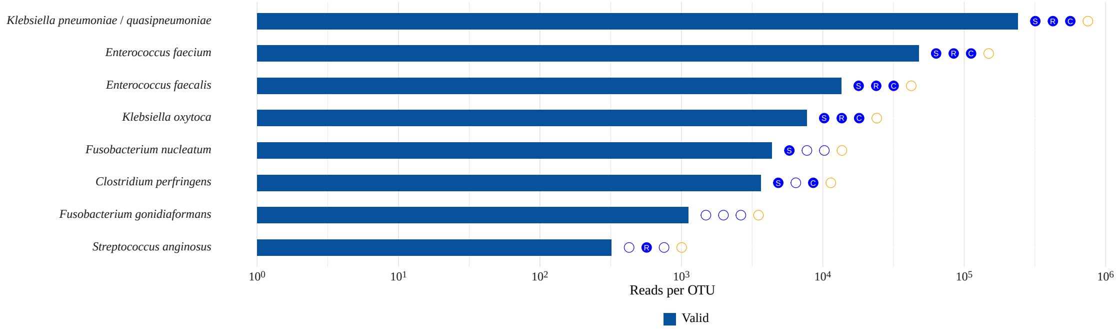

Sample 15

16S−PCR Ct−value: 27.6 | Number of valid reads: 320315

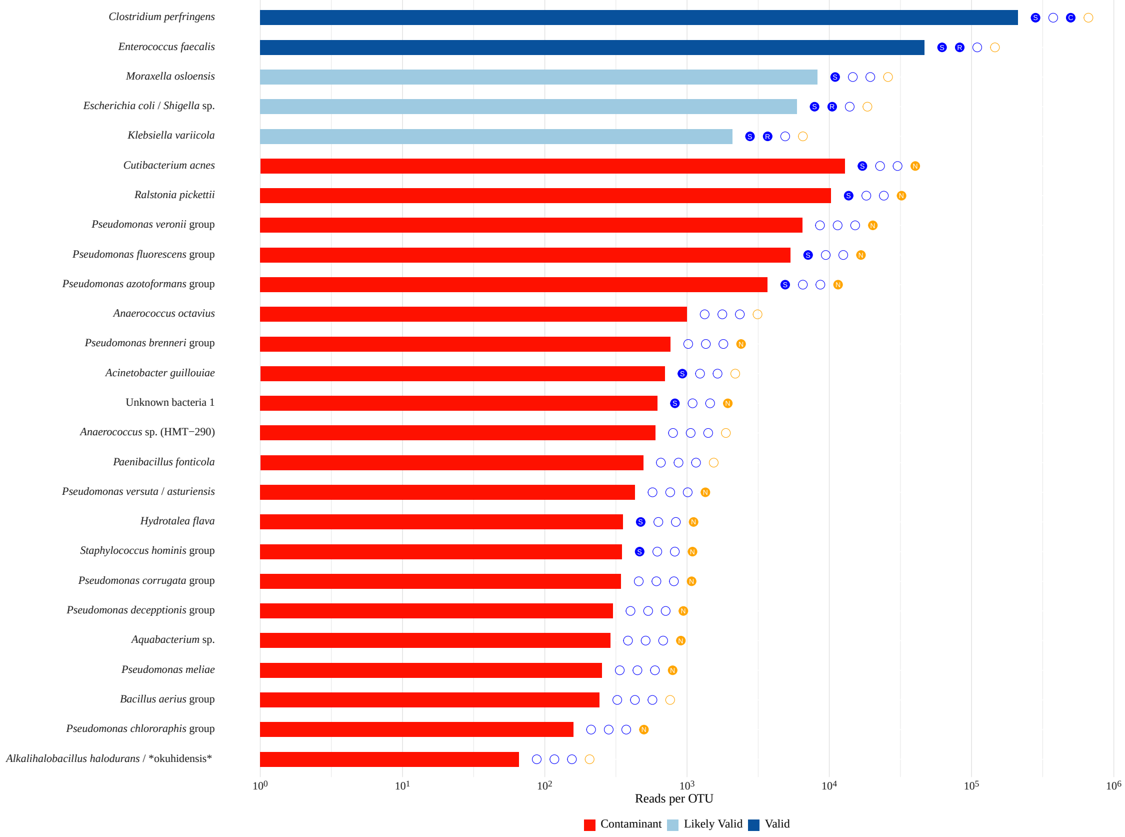

Sample 16-41: Biles samples from patiens with non-infectious bile duct stenosis caused by bile duct stone.

Sample 16

16S–PCR Ct–value:22.2 | Number of valid reads: 108617

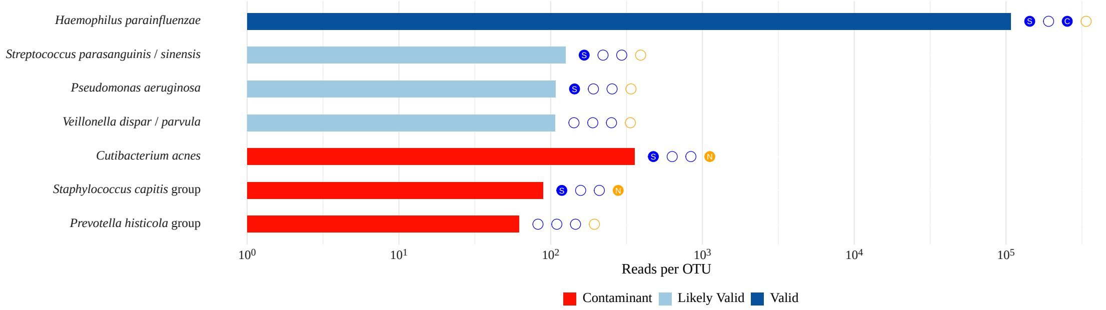

Sample 17

16S−PCR Ct−value: 33.4 | Number of valid reads: 123825

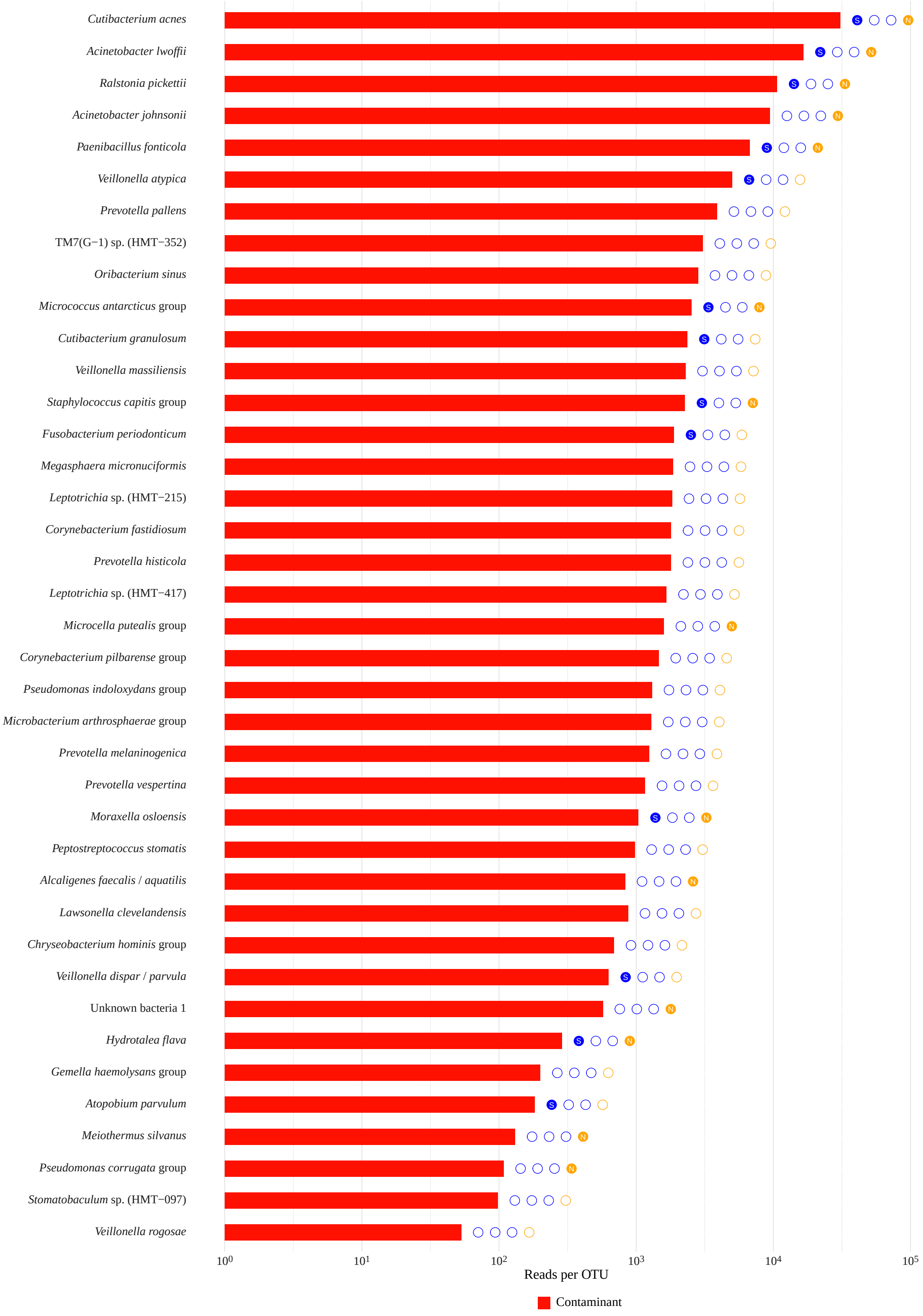

Sample 18

16S−PCR Ct−value: 30.6 | Number of valid reads: 164345

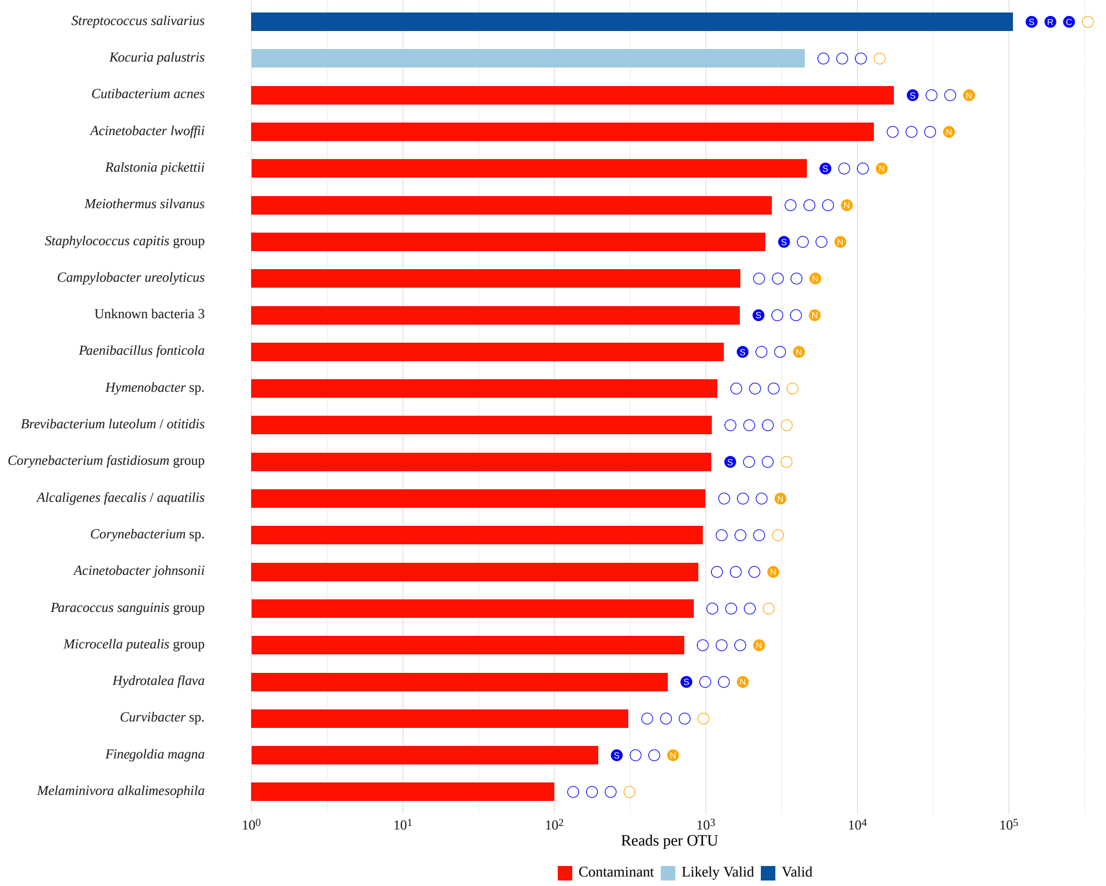

Sample 19

16S−PCR Ct−value: 12.2 | Number of valid reads: 158615

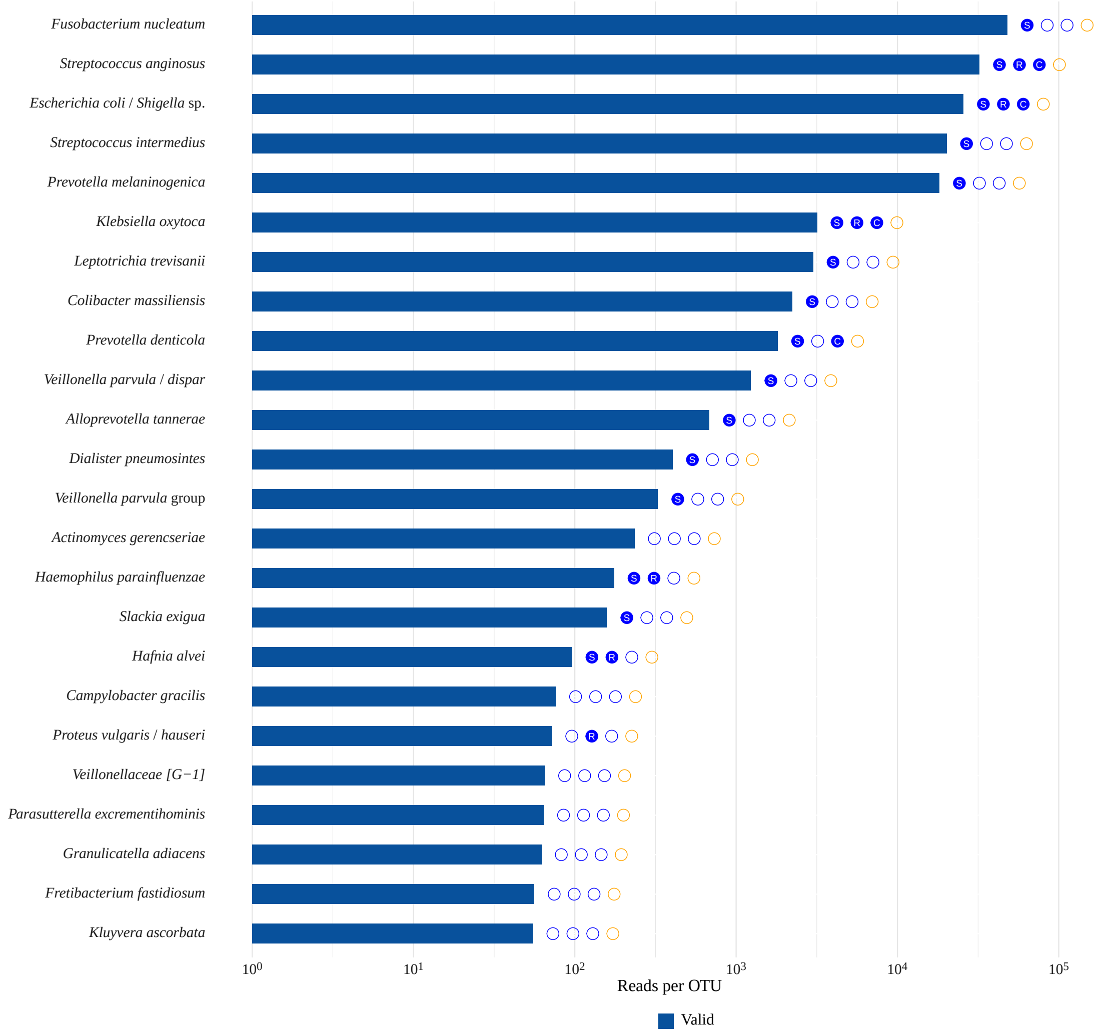

Sample 20

16S−PCR Ct−value: 33.3 | Number of valid reads: 85122

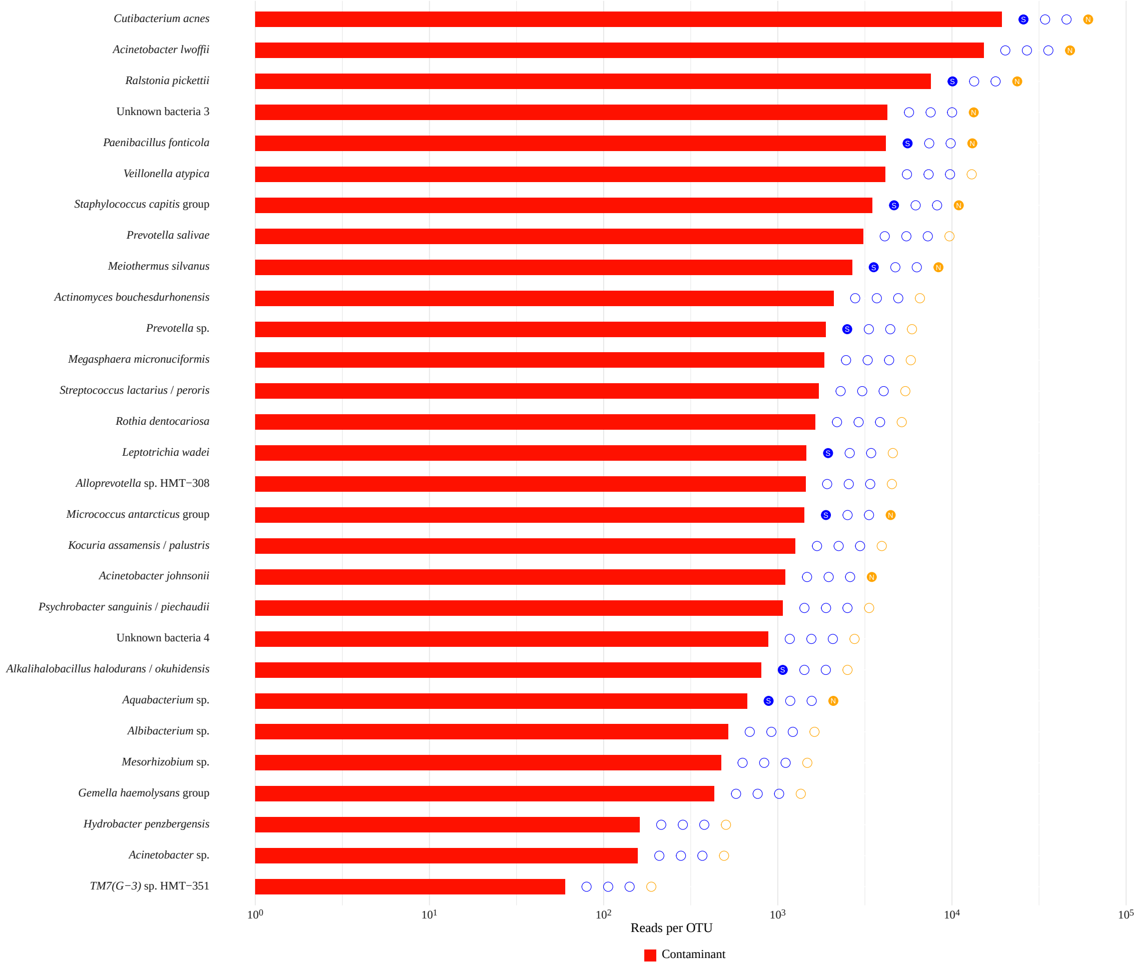

Sample 21

16S−PCR Ct−value: 13.7 | Number of valid reads: 216637

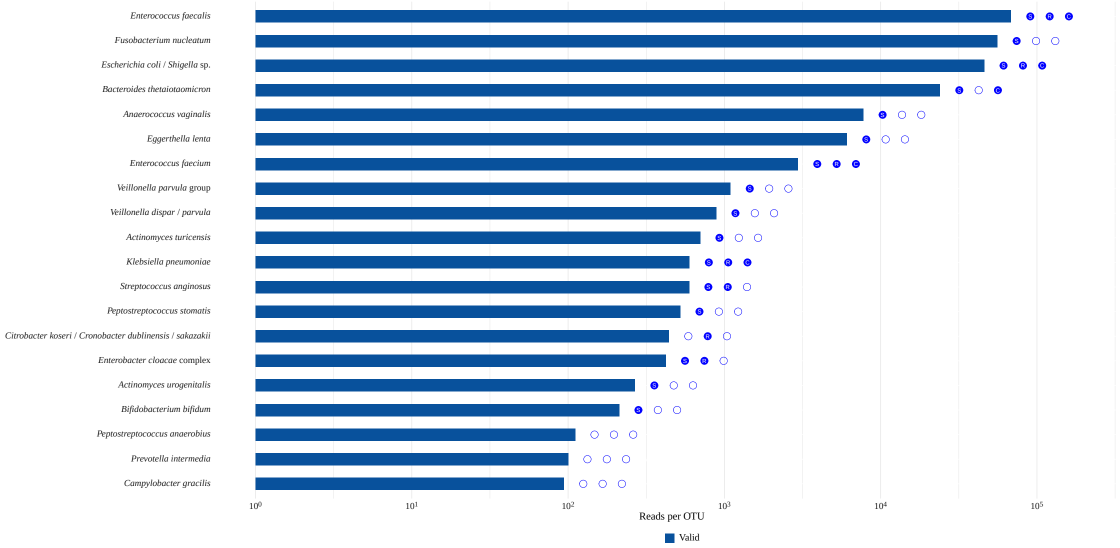

Sample 22

16S−PCR Ct−value: 30.9 | Number of valid reads: 76201

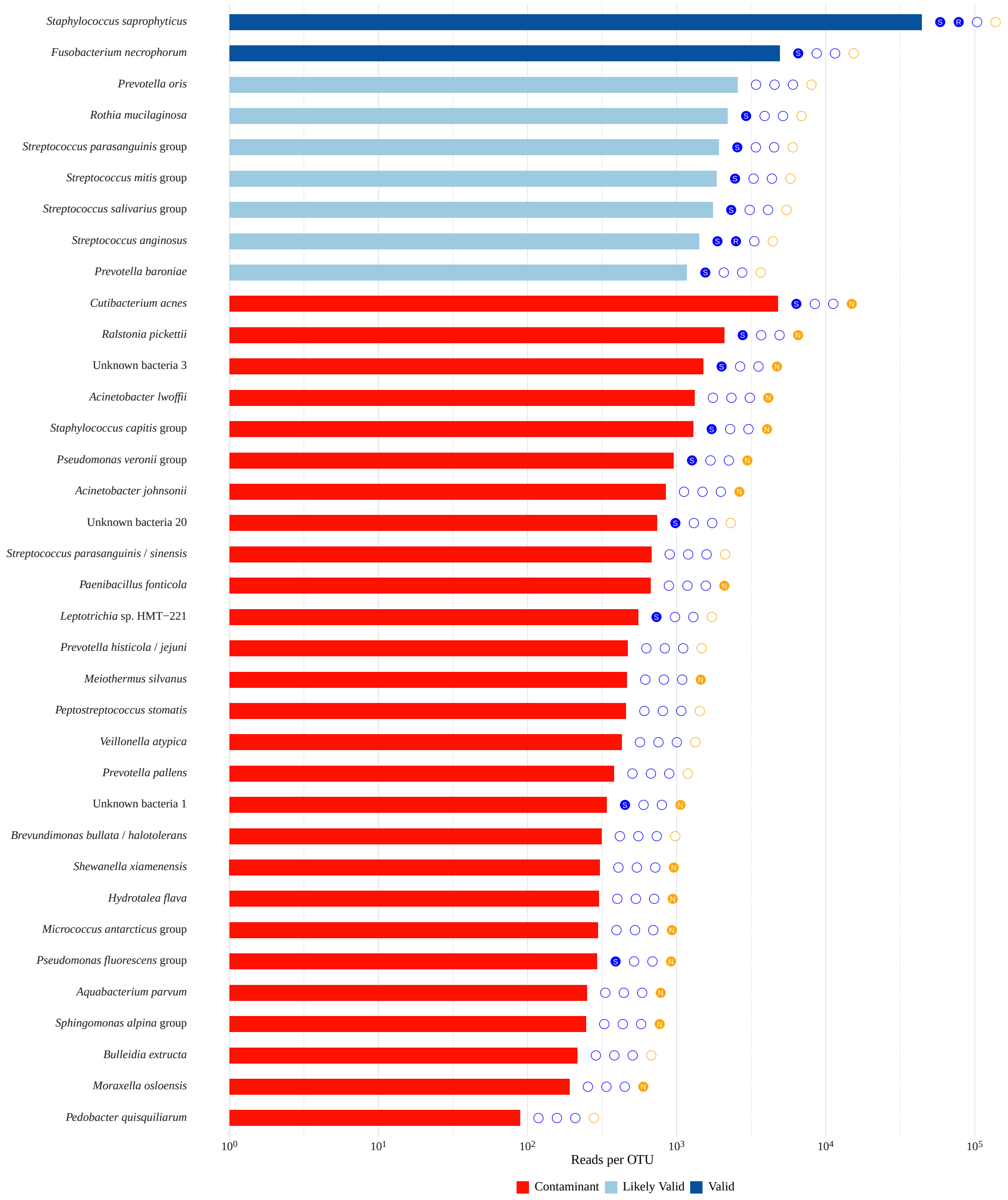

Sample 23

16S−PCR Ct−value: 29.9 | Number of valid reads: 97083

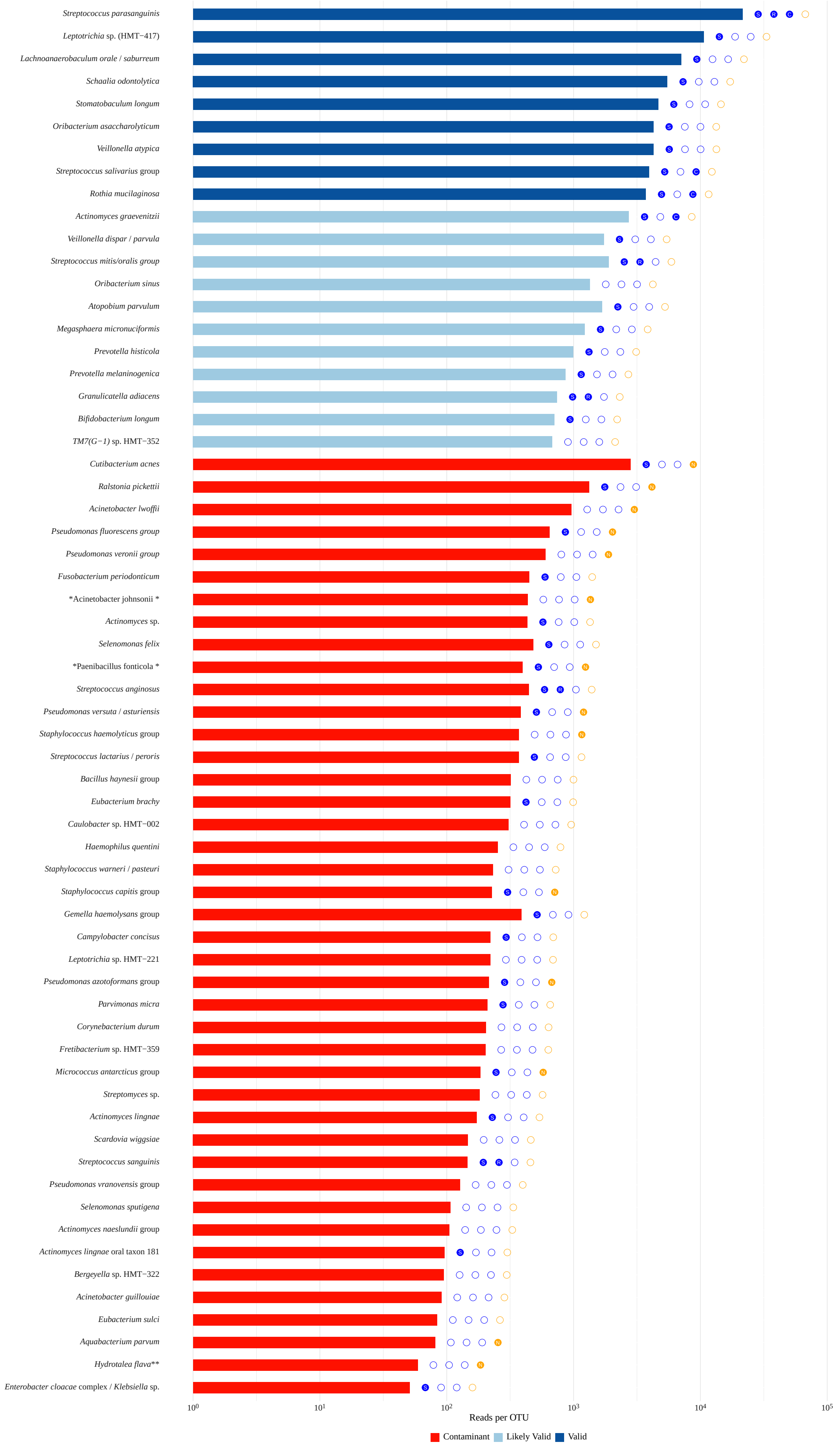

Sample 24

16S−PCR Ct−value: 33.3 | Number of valid reads: 84713

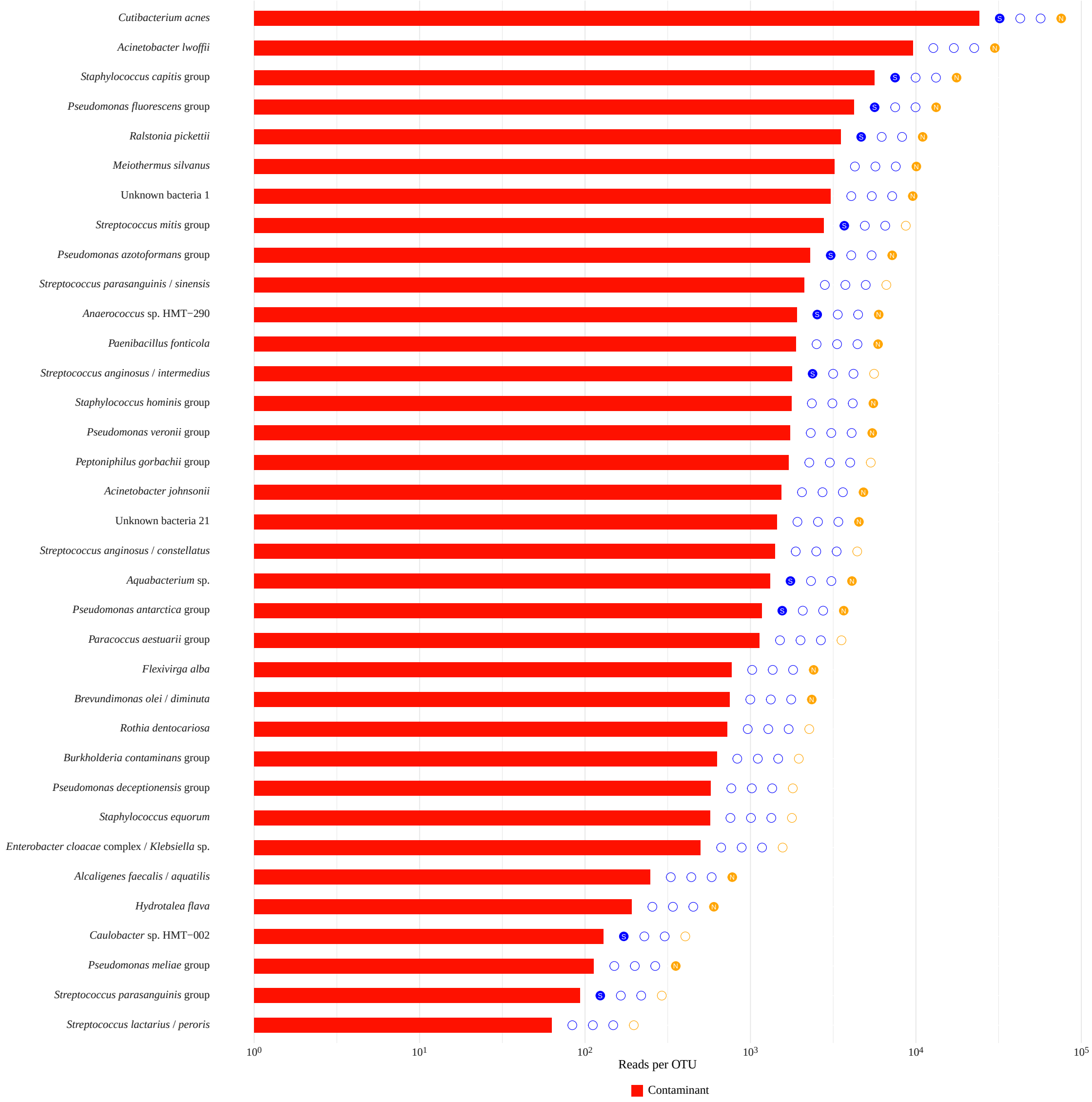

Sample 25

16S−PCR Ct−value: 26.8 | Number of valid reads: 94801

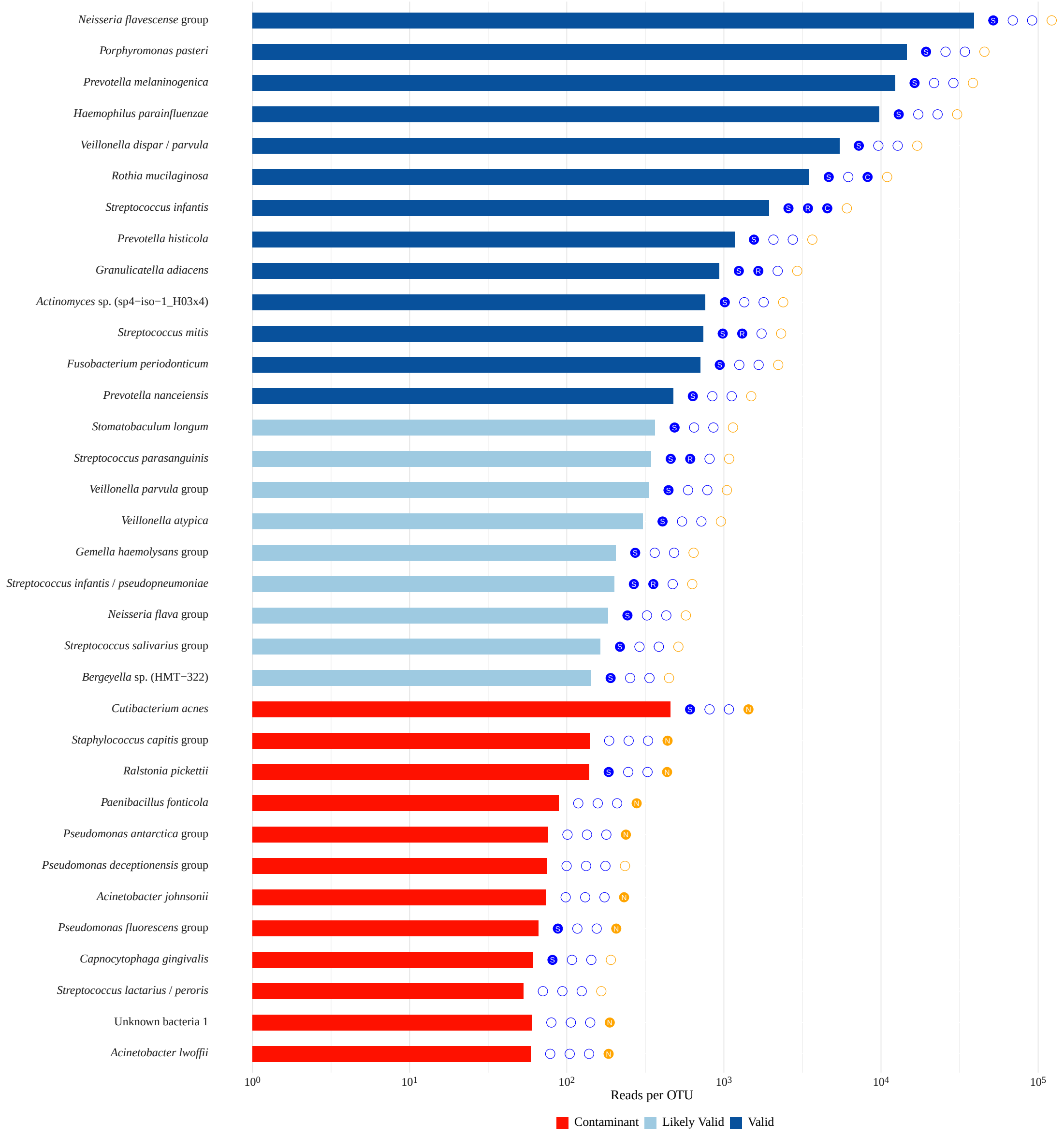

Sample 26

16S−PCR Ct−value: 25.8 | Number of valid reads: 198331

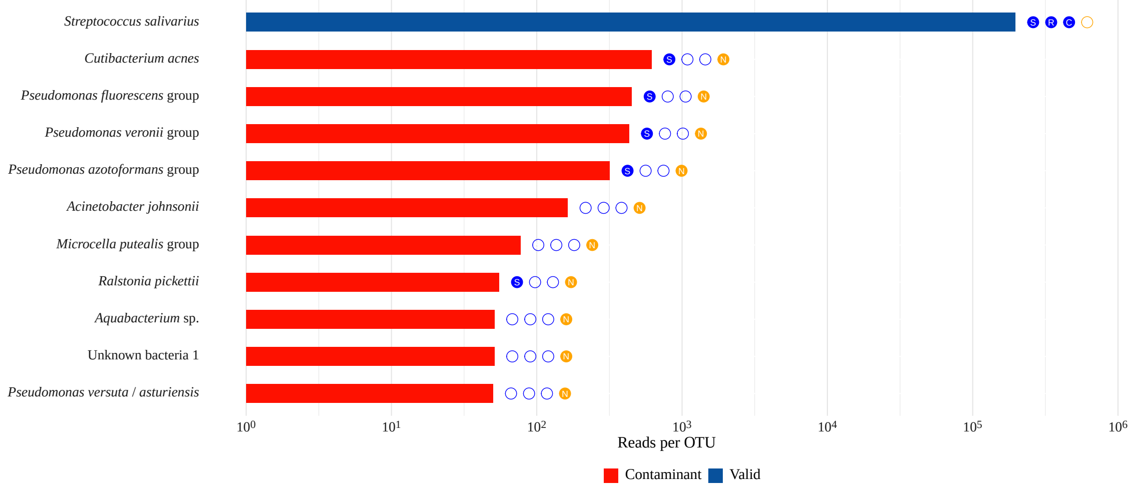

Sample 27

16S−PCR Ct−value: 33.3 | Number of valid reads: 122790

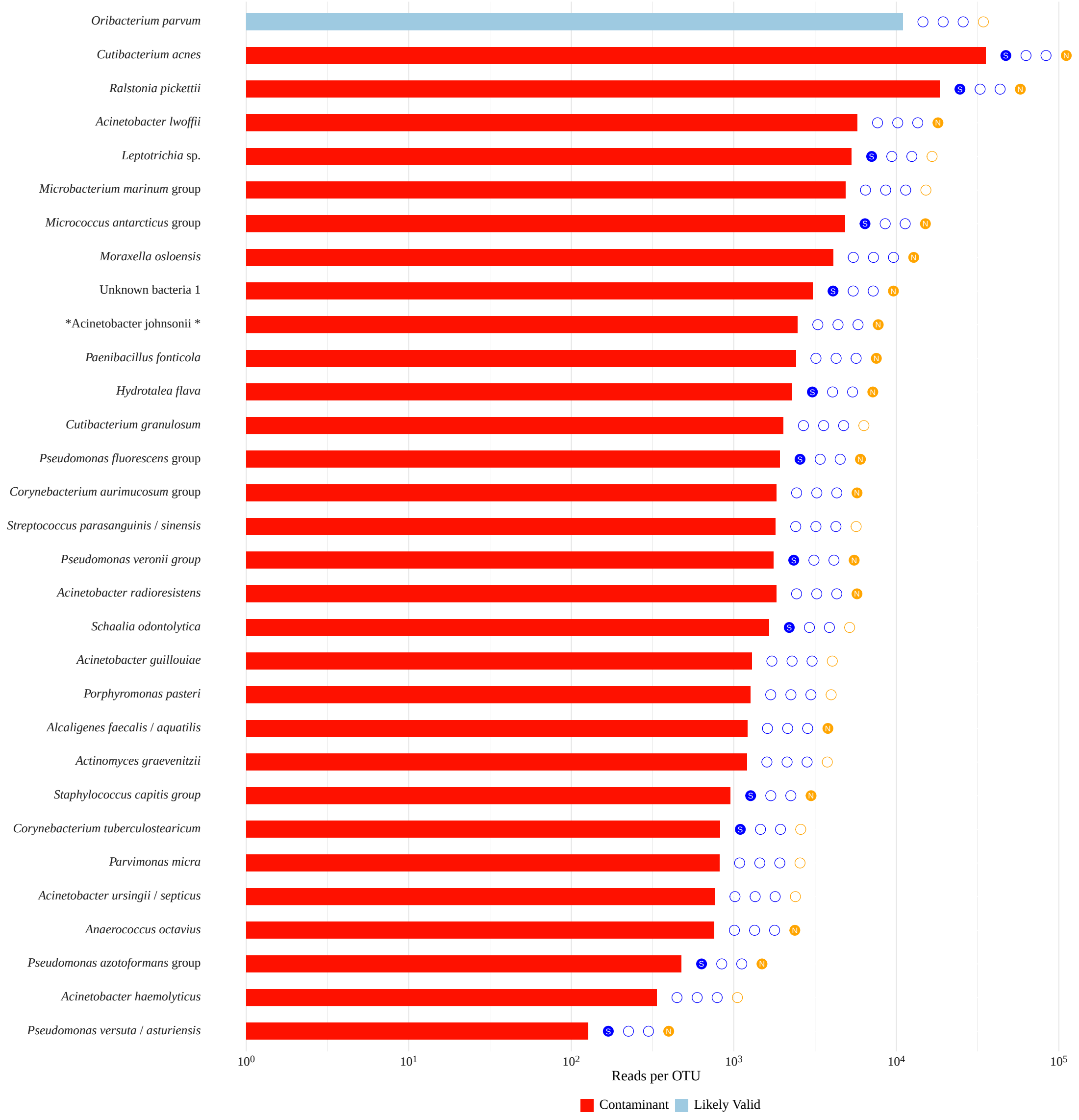

Sample 28

16S−PCR Ct−value: 30.6 | Number of valid reads: 101113

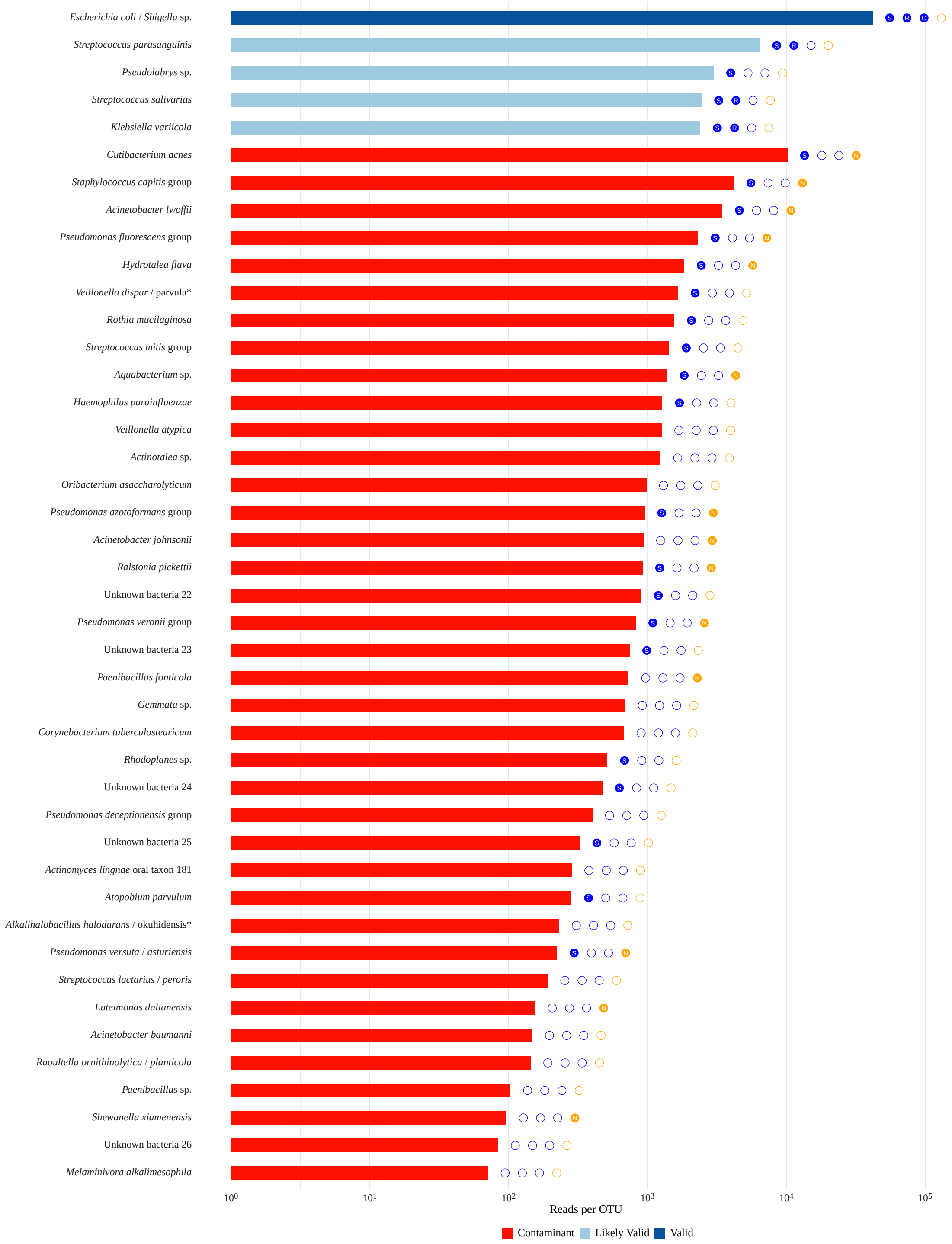

Sample 29

16S−PCR Ct−value: 16.3 | Number of valid reads: 583052

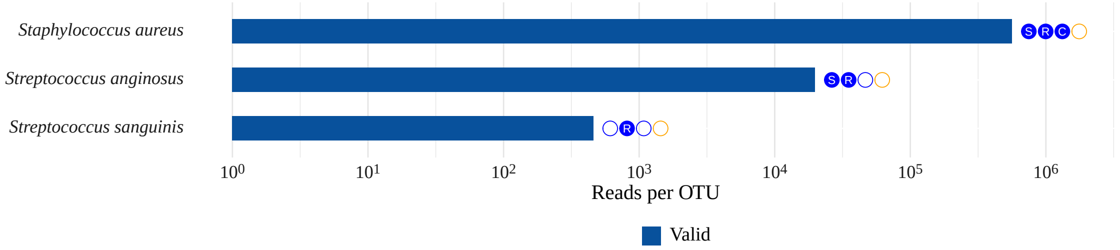

Sample 30

16S−PCR Ct−value: 32.8 | Number of valid reads: 241529

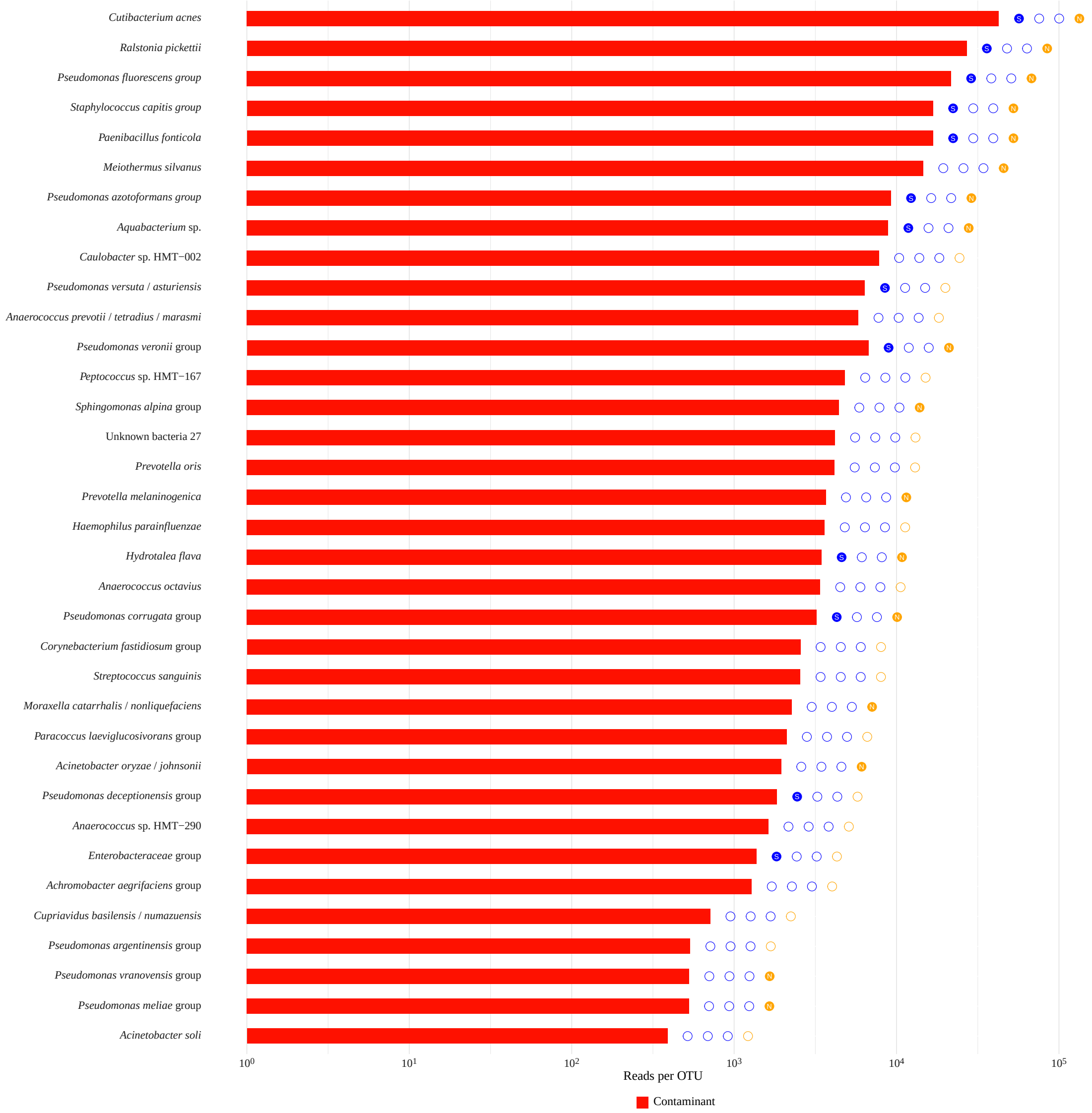

Sample 31

16S−PCR Ct−value: 21.1 | Number of valid reads: 534013

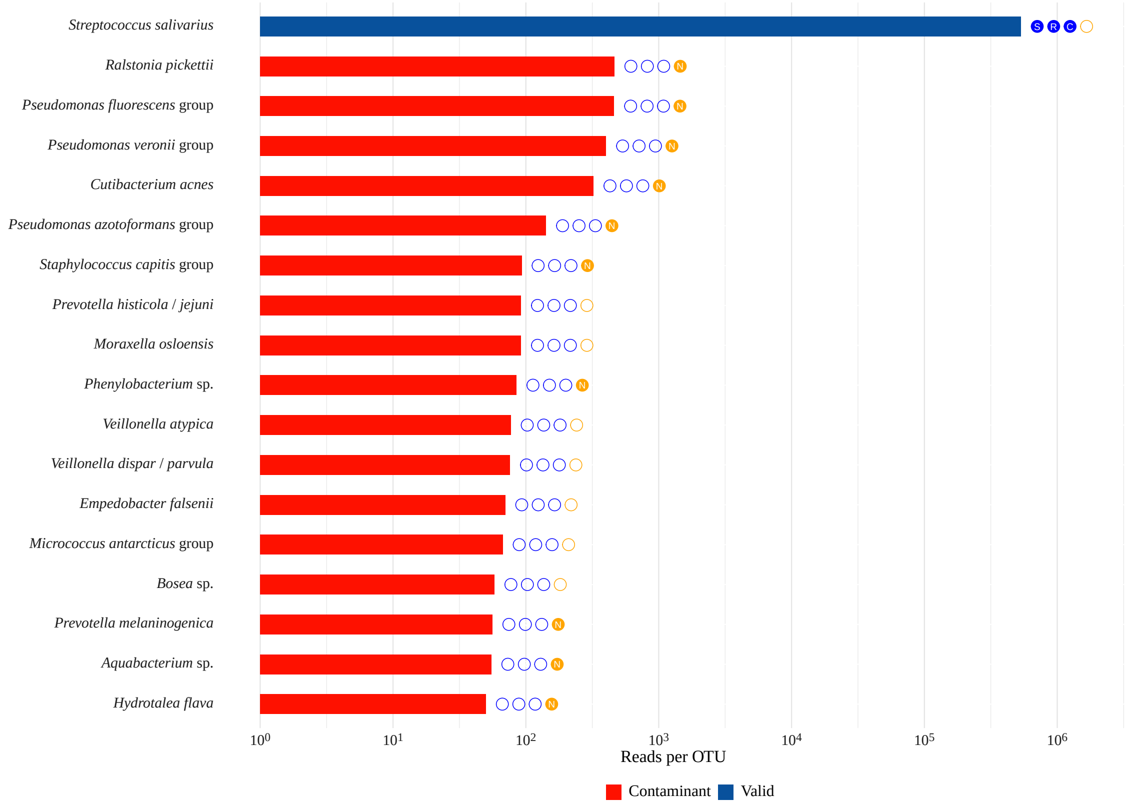

Sample 32

16S−PCR Ct−value: 21.3 | Number of valid reads: 423836

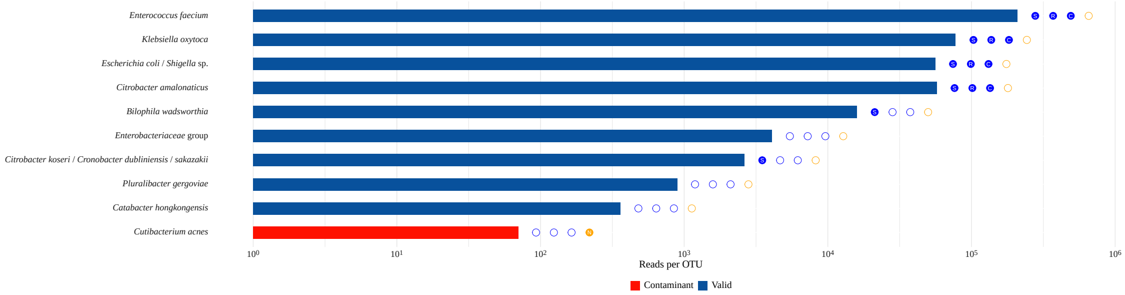

Sample 33

16S−PCR Ct−value: 24.9 | Number of valid reads: 362616

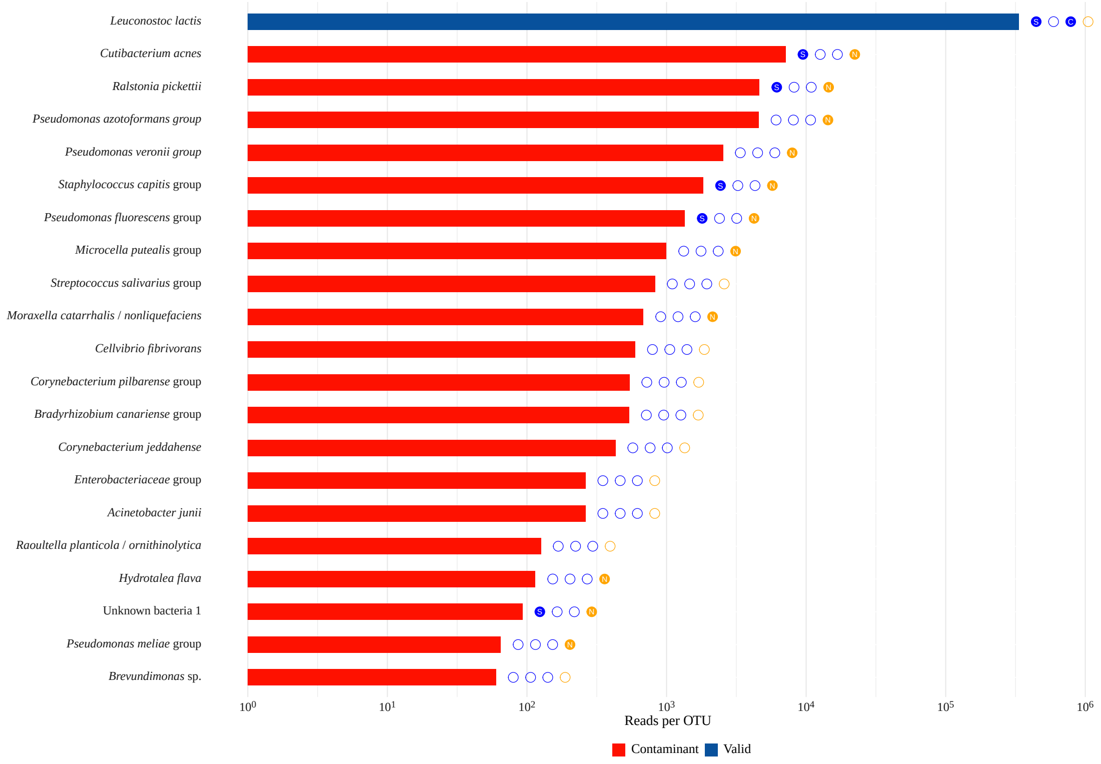

Sample 34

16S−PCR Ct−value: 31.7 | Number of valid reads: 284675

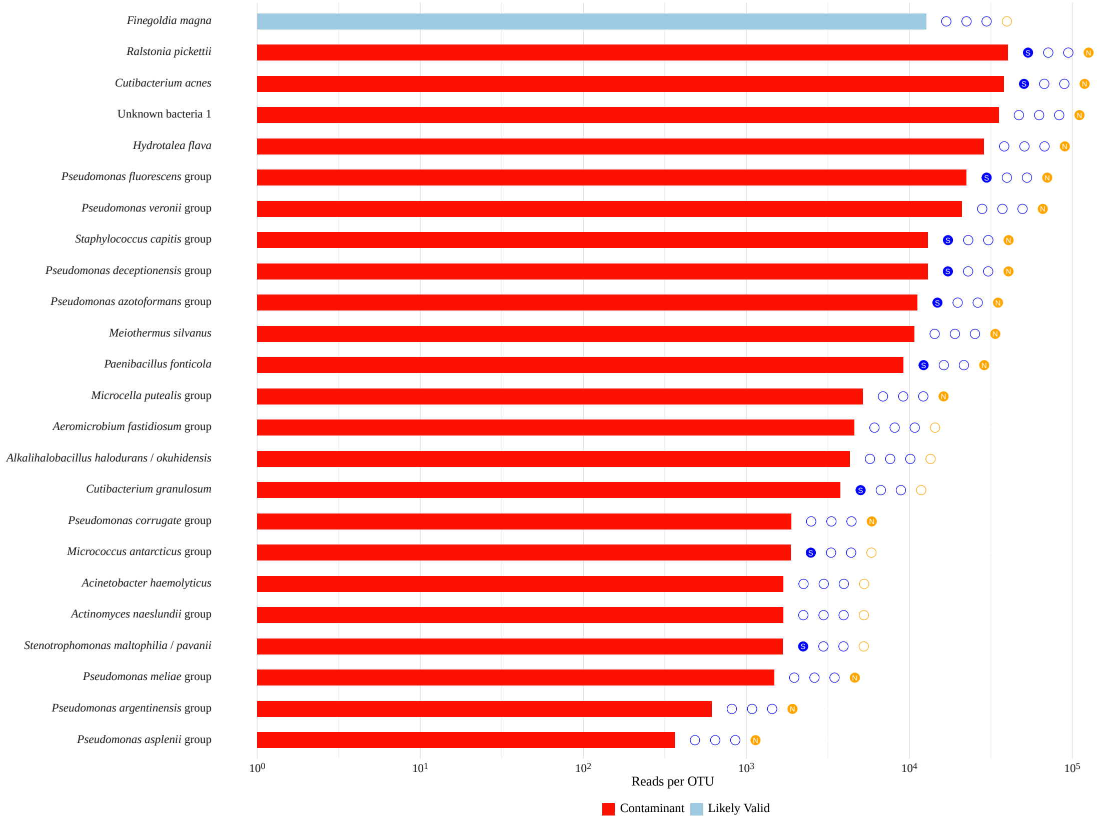

Sample 35

16S−PCR Ct−value: 33 | Number of valid reads: 311709

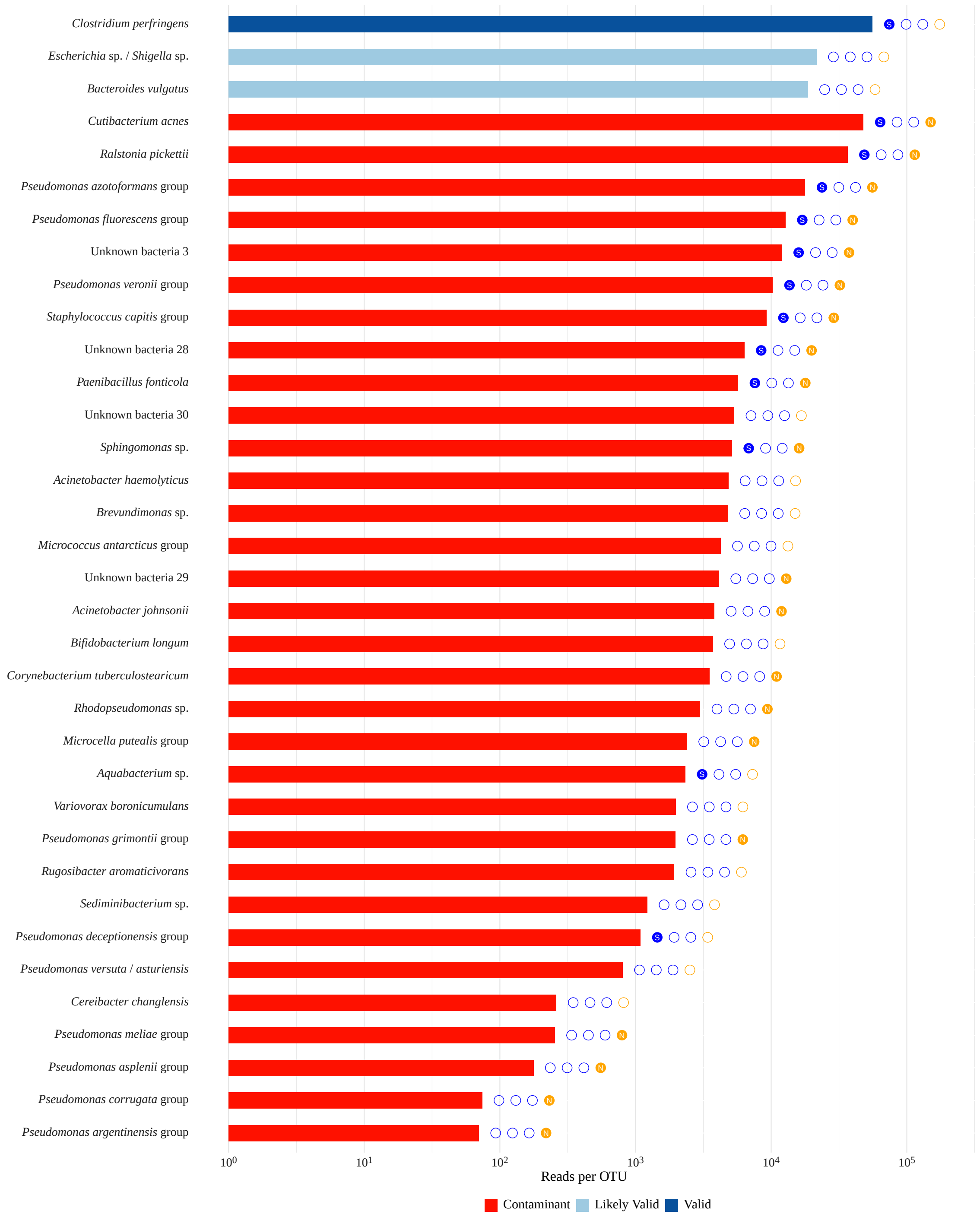

Sample 36

16S−PCR Ct−value: 12.9 | Number of valid reads: 249750

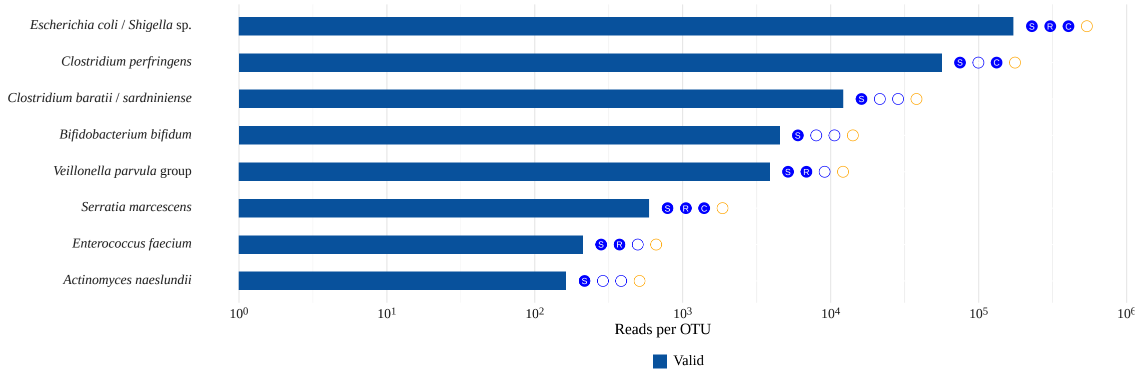

Sample 37

16S−PCR Ct−value: 33.4 | Number of valid reads: 289331

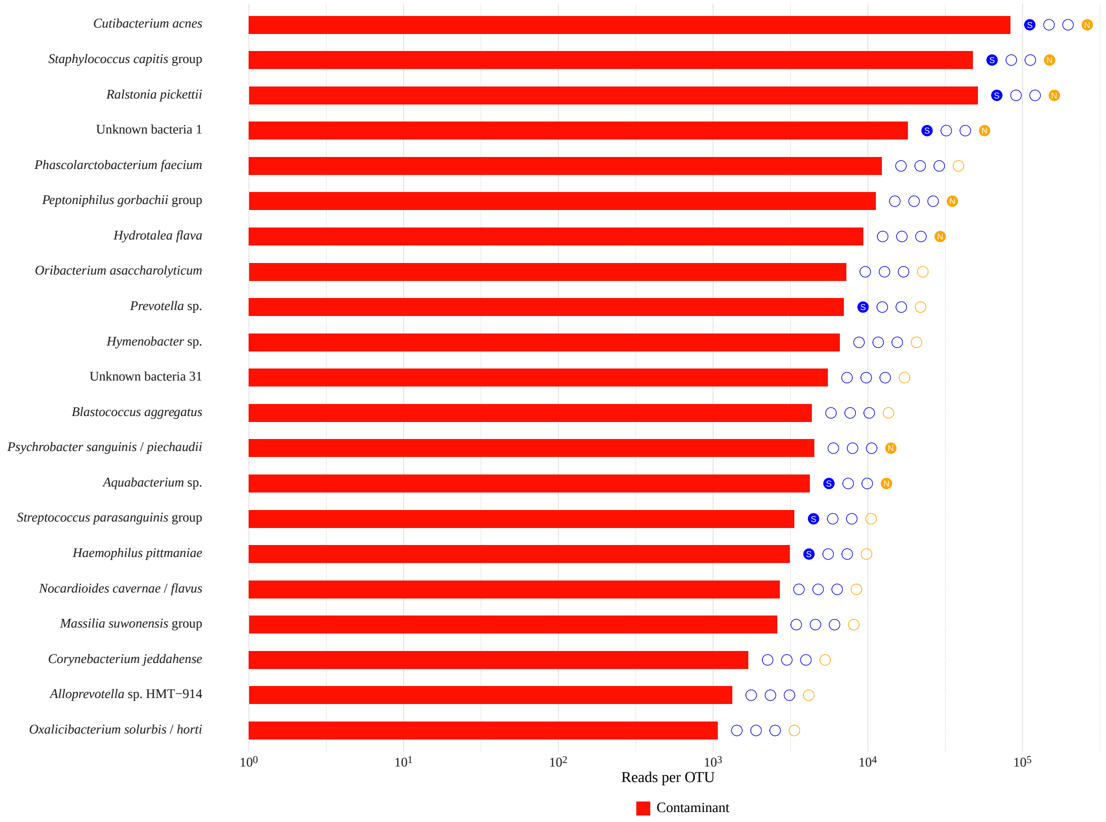

Sample 38

16S−PCR Ct−value: 22.4 | Number of valid reads: 188962

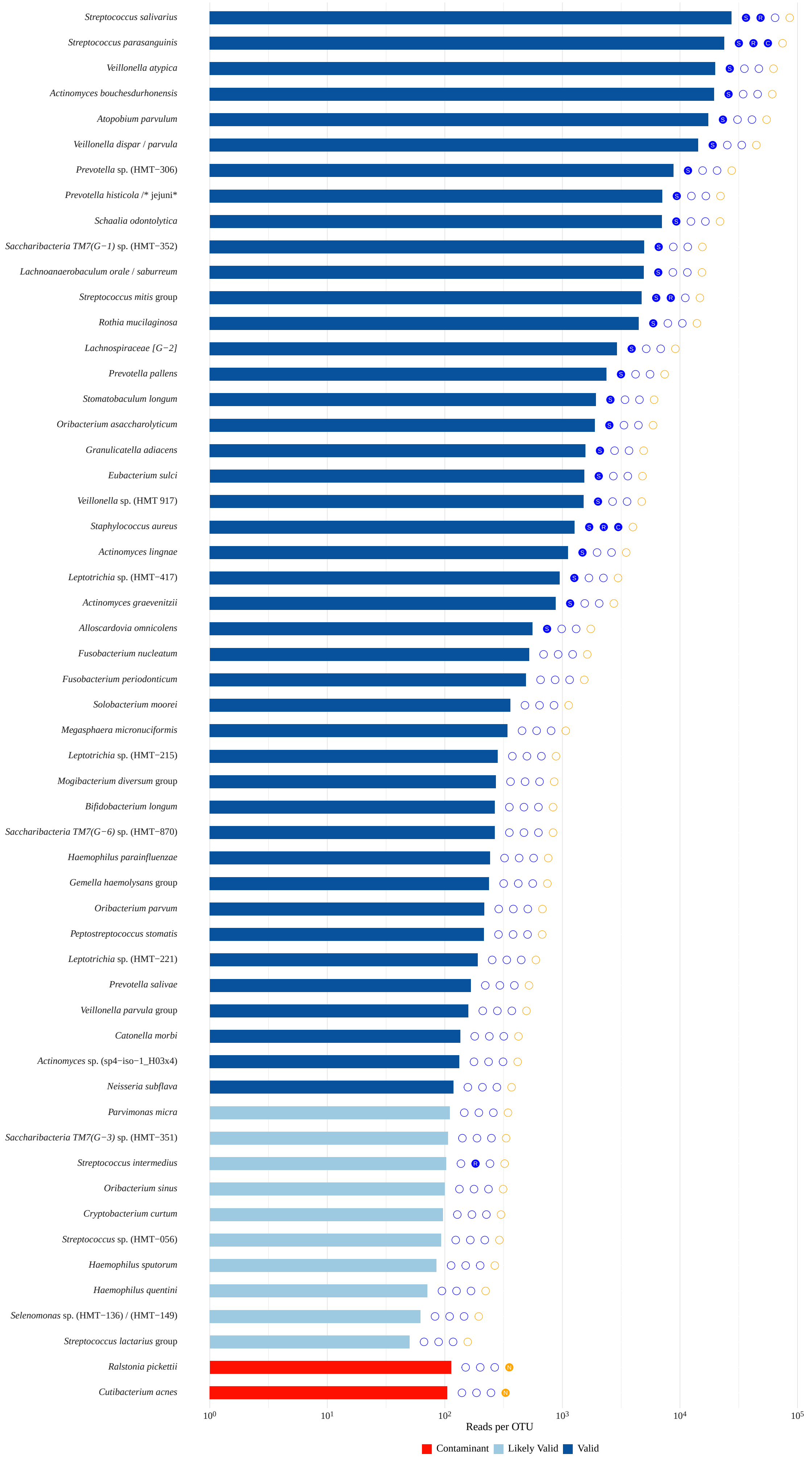

Sample 39

16S−PCR Ct−value: 17 | Number of valid reads: 145773

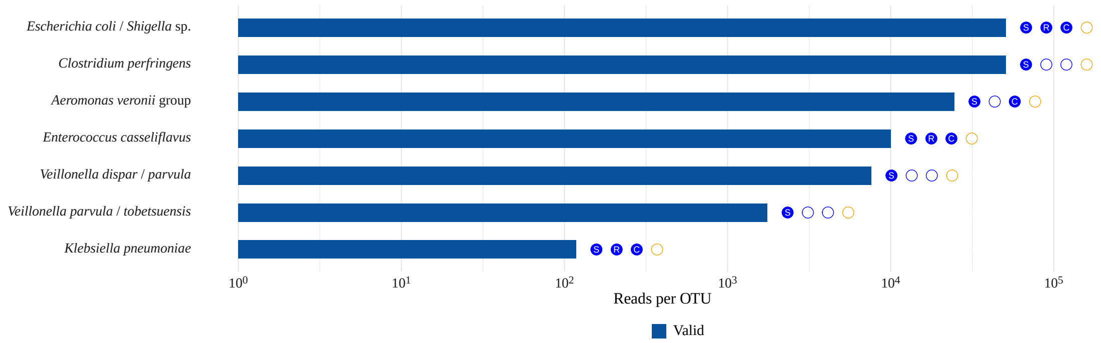

Sample 40

16S−PCR Ct−value: 14.4 | Number of valid reads: 342012

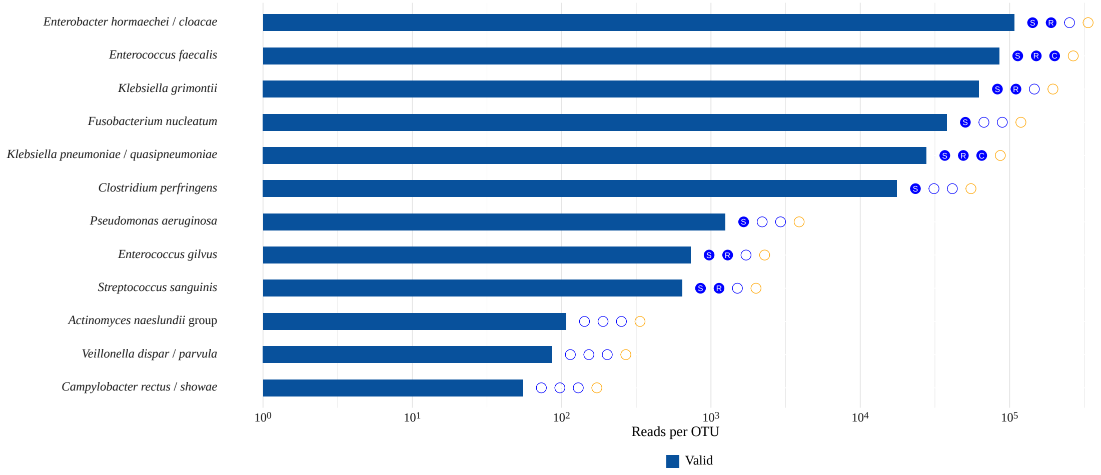

Sample 41

16S−PCR Ct−value: 32.3 | Number of valid reads: 173034

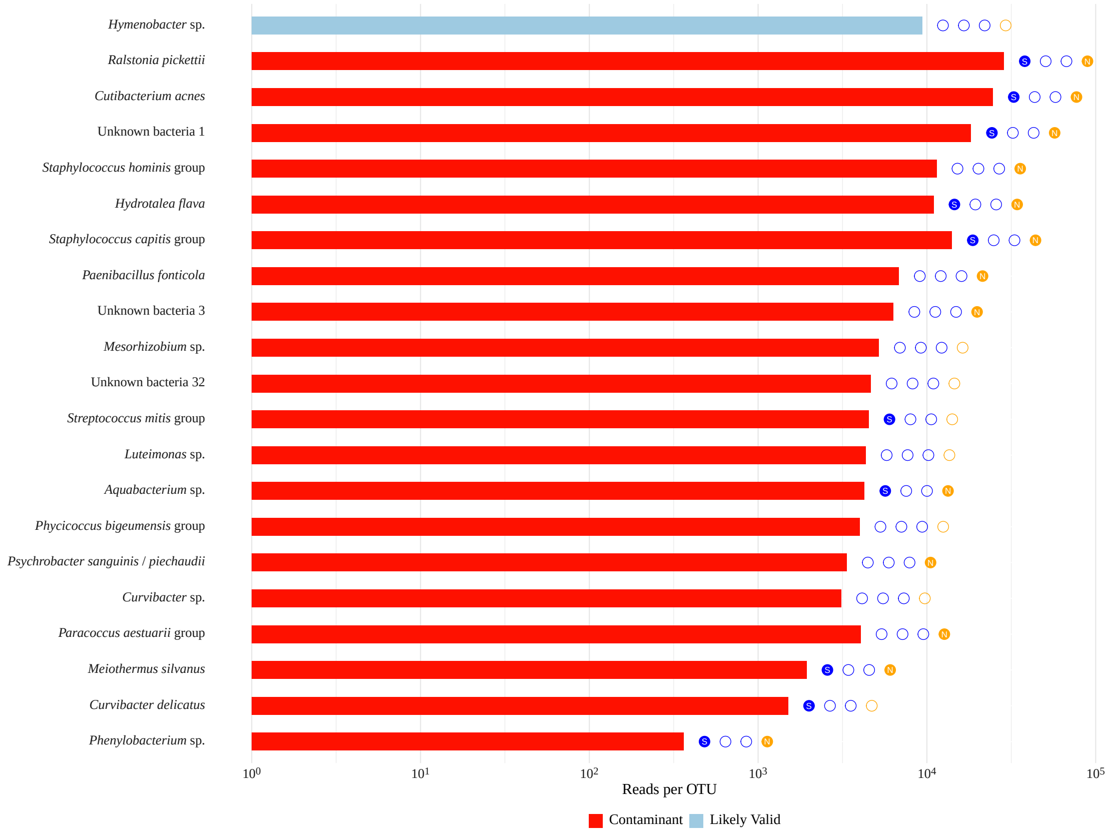

Supplement: FIG S1 [file mbio.00598-21-sf001.pdf]
