# Supplementary material for: Managing Contamination and Diverse Bacterial Loads in 16S rRNA Deep Sequencing of Clinical Samples: Implications of the Law of Small Numbers
Source: mBio. 2021 Jun 8;12(3):e00598-21. doi: 10.1128/mBio.00598-21 (PMC8262989; doi:10.1128/mBio.00598-21)
Supplement: TABLE S7 [file mbio.00598-21-st007.pdf]

Supplementary Table S7: Species identified at a higher taxonomic level with use of partial rpoB -gene compared to partial 16S rRNA gene sequencing (V3–V4).

	16S rRNA gene sequencing results	rpoB- gene sequencing results
1	*Citrobacter amalonaticus / Citrobacter farmeri*	*Citrobacter amalonaticus*
2	*Enterobacter hormaechei / Enterobacter cloacae / Klebsiella grimontii / Klebsiella michiganensis / Klebsiella oxytoca*	*Enterobacter cloacae / Enterobacter hormaechei*
3	*Enterobacter kobei / Enterobacter ludwigii / Enterobacter cloacae / Salmonella enterica*	*Enterobacter cloacae* complex
4	*Enterococcus avium / Enterococcus devriesei / Enterococcus gilvus / Enterococcus pseudoavium / Enterococcus viikkiensis / Enterococcus canintestini / Enterococcus gilvus / Enterococcus malodoratus / Enterococcus raffinosus / Enterococcus xianfangensis / Enterococcus casseliflavus / Enterococcus dispar / Enterococcus hermanniensis*	*Enterococcus gilvus*
5	*Enterococcus casseliflavus / Enterococcus canintestini / Enterococcus gallinarum / Enterococcus saigonensis / Enterococcus devriesei / Enterococcus dispar / Enterococcus gilvus / Enterococcus viikkiensis*	*Enterococcus casseliflavus*
6	*Enterococcus durans / Enterococcus faecium / Enterococcus hirae / Enterococcus sanguinicola / Enterococcus casseliflavus / Enterococcus mundtii / Enterococcus ratti / Enterococcus villorum*	*Enterococcus faecium*
7	*Escherichia albertii / Escherichia coli / Escherichia fergusonii / Escherichia marmotae / Shigella* sp.	*Escherichia coli / Shigella* sp.
8	*Klebsiella grimontii / Klebsiella oxytoca / Salmonella enterica*	*Klebsiella grimontii*
9	*Klebsiella michiganensis / Klebsiella oxytoca / Enterobacter asburiae / Enterobacter hormaechei / Enterobacter cloacae*	*Klebsiella oxytoca*
10	*Klebsiella oxytoca / Salmonella enterica*	*Klebsiella oxytoca*
11	*Klebsiella pneumoniae / Klebsiella quasipneumoniae / Enterobacter asburiae / Enterobacter bugandensis / Enterobacter cancerogenus / Enterobacter cloacae / Enterobacter hormaechei*	*Klebsiella pneumoniae*
12	*Klebsiella pneumoniae / Klebsiella variicola*	*Klebsiella pneumoniae*
13	*Klebsiella pneumoniae / Klebsiella variicola*	*Klebsiella variicola*
14	*Proteus cibarius / Proteus hauseri / Proteus terrae / Proteus vulgaris*	*Proteus vulgaris / Proteus hauseri*

15	*Serratia odorifera / Yersinia enterocolitica / Yersinia rohdei*	*Serratia odorifera*
16	*Staphylococcus aureus / Staphylococcus croceolyticus / Staphylococcus petrasii*	*Staphylococcus aureus*
17	*Staphylococcus haemolyticus / Staphylococcus croceolyticus / Staphylococcus petrasii / Staphylococcus epidermidis / Staphylococcus capitis / Staphylococcus caprae / Staphylococcus hominis*	*Staphylococcus haemolyticus*
18	*Staphylococcus saprophyticus / Staphylococcus xylosus / Staphylococcus gallinarum*	*Staphylococcus saprophyticus*
19	*Staphylococcus warneri / Staphylococcus pasteuri*	*Staphylococcus warneri*
20	*Streptococcus anginosus / Streptococcus intermedius*	*Streptococcus intermedius*
21	*Streptococcus anginosus / Streptococcus intermedius*	*Streptococcus anginosus*
22	*Streptococcus mitis/oralis group*	*Streptococcus infantis*
23	*Streptococcus mitis/oralis group*	*Streptococcus mitis*
24	*Streptococcus mitis/oralis group*	*Streptococcus oralis*
