# Supplementary material for: Managing Contamination and Diverse Bacterial Loads in 16S rRNA Deep Sequencing of Clinical Samples: Implications of the Law of Small Numbers
Source: mBio. 2021 Jun 8;12(3):e00598-21. doi: 10.1128/mBio.00598-21 (PMC8262989; doi:10.1128/mBio.00598-21)
Supplement: TABLE S8 [file mbio.00598-21-st008.pdf]

Supplementary Table S8:

A) Primers with adapter sequences (1). Sequences of the target specific portions in capital letters.

Name	Sequence	Position[a]
16S-F[b]	tcgtcggcagcgtcagatgtgtataagagacagCCTACGGGNGGCWGCAG	340-356
16S-R[b]	gtctcgtgggctcggagatgtgtataagagacagGACTACCAGGGTATCTAAKCC	784-803
rpoB_Ent-F	tcgtcggcagcgtcagatgtgtataagagacagGAAGGTCCRAAYATCGGTCT	1693-1712
rpoB_Ent-R	gtctcgtgggctcggagatgtgtataagagacagTGCATGTTCGCACCCAT	2041-2057
rpoB_ESS-F1	tcgtcggcagcgtcagatgtgtataagagacagGCRACAGCRTGTATYCCRTTC	1861-1881
rpoB_ESS-F2[c]	tcgtcggcagcgtcagatgtgtataagagacagGCDACMGCWTGTATYCCWTTC	1861-1881
rpoB_ESS-R	gtctcgtgggctcggagatgtgtataagagacagGTTRTAMCCNTCCCAWGTCAT	2287-2307

[a] Positions for 16S based on *Escherichia coli* (GenBank accession J01859). Positions for RpoB_ESS based on *Staphylococcus aureus* [*rpoB* coding sequence (CDS); GenBank accession X64172]. Positions for RpoB_Ent based on *Escherichia coli* [*rpoB* coding sequence (CDS); GenBank accession V00340].

[b] Abbreviations: F = forward primer. R = reverse primer.

[c] The modifications in rpoB_ESS, which were made for better coverage of *Enterococcus raffinosus,* are marked with red.

1. Dyrhovden R, Ovrebo KK, Nordahl MV, Nygaard RM, Ulvestad E, Kommedal O. 2019. Bacteria and fungi in acute cholecystitis. A prospective study comparing next generation sequencing to culture. J Infect doi:10.1016/j.jinf.2019.09.015.

B) PCR mixture for the different gene targets and the temperature profile of the amplicon PCR

Target gene	Primer name	Concentration and volume - primer	Volume - enzyme (µl)	Volume - H2O (µl)	Volume - template (µl)	Temperature profile (all targets)
16S rRNA, V3-V4	16S-F<br>16S-R	0,4 µM/ 1,0 µl<br>0,4 µM/ 1,0 µl	12,5	8,5	2,0	95 °C for 3 min (activation)<br>45 cycles of:
*rpoB*_Ent *(*targeting Enterobacteriaceae)	*rpoB*_Ent-F<br>*rpoB*_Ent-R	0,4 µM/ 1,0 µl<br>0,4 µM/ 1,0 µl	12,5	8,5	2,0	- 95 °C for 60 s (melting)<br>- 60 °C for 30 s (annealing)
*rpoB*_ESS (targeting *Stapyholococcus, Streptococcus* and *Enterococcus)*	*rpoB*_ESS-F1<br>*rpoB*_ESS-F2<br>*rpoB*_ESS-R	0,4 µM/ 1,0 µl<br>0,4 µM/ 1,0 µl<br>0,6 µM/ 1,5 µl	12,5	8,0	2,0	- 72 °C for 30 s (annealing)<br>Melting curve analysis:<br>- 95 °C for 60 s<br>- 40 °C for 2 min<br>- 95 °C continous<br>40 °C for 30 s (cooling)
