# Supplementary material for: Managing Contamination and Diverse Bacterial Loads in 16S rRNA Deep Sequencing of Clinical Samples: Implications of the Law of Small Numbers
Source: mBio. 2021 Jun 8;12(3):e00598-21. doi: 10.1128/mBio.00598-21 (PMC8262989; doi:10.1128/mBio.00598-21)
Supplement: TABLE S9 [file mbio.00598-21-st009.pdf]

Supplementary Table S9: Species included in species groups (S9A), and best BLAST search match for bacteria termed as "Unknown bacteria #" (S9B).

A)

Name of species group	Species included
Achromobacter aegrifaciens group	Achromobacter aegrifaciens / Achromobacter insuavis / Achromobacter marplatensis / Achromobacter deleyi / Achromobacter spanius / Achromobacter kerstersii / Achromobacter pestifer / Achromobacter piechaudii / Achromobacter xylosoxidans
Acidovorax temperans group	Acidovorax temperans / Acidovorax delafieldii / Acidovorax facilis / Acidovorax radicis
Actinomyces naeslundii group	Actinomyces naeslundii / Actinomyces johnsonii / Actinomyces oralis / Actinomyces oris
Actinomyces oris group	Actinomyces oris / Actinomyces viscosus / Actinomyces sp. / Actinomyces johnsonii / Actinomyces oralis / Actinomyces naeslundii / Actinomyces bowdenii
Aeromicrobium fastidiosum group	Aeromicrobium fastidiosum / Aeromicrobium ginsengisoli / Aeromicrobium erythreum / Aeromicrobium panaciterrae / Aeromicrobium choanae / Aeromicrobium tamlense
Aeromonas veronii group	Aeromonas veronii / Aeromonas allosaccharophila / Aeromonas australiensis / Aeromonas lacus / Aeromonas rivipollensis
Agrococcus citreus group	Agrococcus citreus / Agrococcus jenensis / Agrococcus baldri / Agrococcus carbonis
Agrobacterium arsenijevicii group	Agrobacterium arsenijevicii / Agrobacterium deltaense / Agrobacterium tumefaciens / Beijerinckia fluminensis / Rhizobium nepotum / Agrobacterium rubi / Neorhizobium alkalisoli / Neorhizobium huautlense / Rhizobium loessense / Rhizobium skierniewicense
Aquabacterium parvum group	Aquabacterium parvum / Imtechium assamiensis / Aquabacterium commune
Bacillus aerius group	Bacillus aerius / Bacillus aerophilus / Bacillus altitudinis / Bacillus stratosphericus / Bacillus xiamenensis / Bacillus australimaris / Bacillus pumilus / Bacillus safensis / Bacillus zhangzhouensis / Peribacillus acanthi
Bacillus haynesii group	Bacillus haynesii / Bacillus licheniformis / Bacillus paralicheniformis / Bacillus piscis / Bacillus sonorensis / Bacillus subtilis / Bacillus glycinifermentans / Bacillus oryzaecorticis / Bacillus swezeyi
Blastococcus litoris group	Blastococcus litoris / Blastococcus colisei / Blastococcus deserti / Blastococcus jejuensis
Brachybacterium horti group	Brachybacterium horti / Brachybacterium nesterenkovii / Brachybacterium rhamnosum / Brachybacterium endophyticum / Brachybacterium zhongshanense / Brachybacterium sacelli / Brachybacterium timonense
Bradyrhizobium canariense group	Bradyrhizobium sp. / Afipia sp. / Pseudomonas carboxydohydrogena / Nitrobacter sp. / Oligotropha carboxidovorans

Burkholderia contaminans group	Burkholderia contaminans / Burkholderia lata / Burkholderia paludis / Burkholderia cenocepacia / Burkholderia cepacia / Burkholderia latens / Burkholderia territorii / Burkholderia ubonensis / Burkholderia vietnamiensis / Burkholderia arboris / Burkholderia metallica / Burkholderia puraquae / Burkholderia seminalis / Burkholderia stabilis
Cellulosimicrobium cellulans group	Cellulosimicrobium cellulans / Cellulosimicrobium funkei / Cellulosimicrobium terreum / Cellulosimicrobium aquatile / Luteimicrobium subarcticum
Chryseobacterium hominis group	Chryseobacterium hominis / Chryseobacterium arachidiradicis / Chryseobacterium bovis
Corynebacterium accolens group	Corynebacterium accolens / Corynebacterium fastidiosum / Corynebacterium macginleyi / Corynebacterium segmentosum / Corynebacterium tuberculostearicum
Corynebacterium amycolatum group	Corynebacterium amycolatum / Corynebacterium lactis / Corynebacterium freneyi / Corynebacterium xerosis
Corynebacterium lipophiloflavum group	Corynebacterium lipophiloflavum / Corynebacterium sanguinis / Corynebacterium senegalense / Corynebacterium mycetoides
Corynebacterium aurimucosum group	Corynebacterium aurimucosum / Corynebacterium minutissimum / Corynebacterium singulare / Corynebacterium phoceense
Corynebacterium fastidiosum group	Corynebacterium fastidiosum / Corynebacterium accolens / Corynebacterium macginleyi / Corynebacterium segmentosum
Corynebacterium gottingense group	Corynebacterium gottingense / Corynebacterium hadale / Corynebacterium imitans / Corynebacterium godavarianum
Corynebacterium mucifaciens group	Corynebacterium mucifaciens / Corynebacterium fournierii / Corynebacterium ihumii / Corynebacterium ureicelerivorans / Corynebacterium pilbarense
Conyzicola nivalis group	Conyzicola nivalis / Cryobacterium tepidiphilum / Cryobacterium mesophilum / Cryobacterium arcticum / Cryobacterium zongtaii / Klugiella xanthotipulae / Leifsonia kafniensis / Leifsonia psychrotolerans / Pseudolysinimonas kribbensis / Chryseoglobus frigidaquae / Glaciihabitans arcticus / Homoserinibacter gongjuensis / Leifsonia poae / Protaetiibacter intestinalis
Corynebacterium pilbarense group	Corynebacterium pilbarense / Corynebacterium mucifaciens / Corynebacterium coyleae / Corynebacterium afermentans / Corynebacterium uhumii
Corynebacterium vitaeruminis group	Corynebacterium vitaeruminis / Corynebacterium ulcerans / Corynebacterium pseudotuberculosis
Deinococcus grandis group:	Deinococcus grandis / Deinococcus soli / Deinococcus daejeonensis / Deinococcus radiotolerans
Dermacoccus nishinomiyaensis group	Dermacoccus nishinomiyaensis / Dermacoccus abyssi / Dermacoccus barathri / Dermacoccus profundi
Dietzia kunjamensis group	Dietzia kunjamensis / Dietzia maris / Dietzia schimae / Dietzia alimentaria
Enterobacteriaceae group	Enterobacter cloacae complex / Salmonella enterica / Leclercia adecarboxylata / Morganella morganii / Pantoea agglomerans

Enterococcus casseliflavus group	Enterococcus casseliflavus / Enterococcus canintestini / Enterococcus gallinarum / Enterococcus saigonensis / Enterococcus casseliflavus / Enterococcus devriesei / Enterococcus dispar / Enterococcus gilvus / Enterococcus pseudoavium / Enterococcus viikiensis
Escherichia albertii group	Escherichia albertii / Escherichia coli / Pseudescherichia vulneris / Escherichia fergusonii / Escherichia marmotae / Shigella sp.
Friedmanniella okinawensis group	Friedmanniella okinawensis / Friedmanniella sagamiharensis / Friedmanniella spumicola
Gemella haemolysans group	Gemella haemolysans / Gemella taiwanensis / Gemella parahaemolysans / Gemella sanguinis
Gordonia namibiensis group	Gordonia namibiensis / Gordonia paraffinivorans / Gordonia rubripertincta / Gordonia westfalica / Gordonia hankookensis / Gordonia hongkongensis / Gordonia hydrophobica / Gordonia lacunae / Gordonia terrae / Gordonia alkanivorans / Gordonia amicalis / Gordonia insulae / Gordonia neofelifaecis / Gordonia spumicola
Kluyvera ascorbata group	Citrobacter europaeus / Enterobacter soli / Kluyvera ascorbata / Kluyvera cryocrescens / Raoultella terrigena / Klebsiella aerogenes
Kocuria flava group	Kocuria flava / Kocuria turfanensis / Kocuria oceani / Kocuria sediminis
Kocuria arsenatis group	Kocuria arsenatis / Kocuria rhizophila / Kocuria tytonicola / Kocuria atrinae / Kocuria carniphila / Kocuria gwangalliensis / Kocuria salsicia / Kocuria varians
Klenkia terrae group	Klenkia terrae / Klenkia brasiliensis / Klenkia taihuensis / Klenkia marina / Klenkia soli
Kribbella endophytica group	Kribbella endophytica / Kribbella flavida / Kribbella italica / Kribbella amoyensis / Kribbella alba / Kribbella karoonensis / Kribbella pittospori / Kribbella swartbergensis
Kribbella hippodromi group	Kribbella hippodromi / Kribbella jejuensis / Kribbella karoonensis / Kribbella podocarpi / Kribbella shirazensis / Kribbella solani / Kribbella soli / Kribbella swartbergensis / Kribbella aluminosa / Kribbella pittospori / Kribbella sindirgiensis
Lactobacillus acidophilus group	Lactobacillus acidophilus / Lactobacillus crispatus / Lactobacillus gallinarum / Lactobacillus helveticus / Lactobacillus kitasatonis
Lactobacillus gasseri group	Lactobacillus gasseri / Lactobacillus paragasseri / Lactobacillus johnsonii / Lactobacillus taiwanensis
Lactobacillus reuteri group	Lactobacillus reuteri / Lactobacillus vaginalis / Lactobacillus antri / Lactobacillus frumenti / Lactobacillus oris
Lactobacillus rhamnosus group	Lactobacillus rhamnosus / Lactobacillus casei / Lactobacillus paracasei / Lactobacillus zeae
Leifsonia aquatica group	Leifsonia aquatica / Leifsonia naganoensis / Leifsonia shinshuensis / Leifsonia xyli / Leifsonia soli / Leifsonia lichenia
Massilia aurea group	Massilia violaceinigra / Massilia aurea / Massilia brevitalea
Massilia suwonensis group	Massilia suwonensis / Massilia niabensis / Massilia haematophila
Massilia mucilaginosa group	Massilia mucilaginosa / Massilia eurypsychrophila / Massilia frigida / Massilia niabensis
Massilia frigida group	Massilia frigida / Massilia mucilaginosa / Massilia rubra / Massilia violaceinigra / Massilia aquatica

Microbacterium arthrosphaerae group	Microbacterium arthrosphaerae / Microbacterium murale / Microbacterium shaanxiense / Microbacterium invictum / Microbacterium lacus / Microbacterium profundi
Microbacterium marinum group	Microbacterium marinum / Microbacterium maritypicum / Microbacterium liquefaciens / Microbacterium oxydans / Microbacterium foliorum / Microbacterium hydrocarbonoxydans / Microbacterium luteolum / Microbacterium phyllosphaerae / Microbacterium saperdae
Microbacterium lemovicicum group	Microbacterium lemovicicum / Microbacterium binotii / Microbacterium diaminobutyricum / Microbacterium endophyticum / Microbacterium neimengense / Microbacterium sediminicola
Microbacterium hominis group	Microbacterium hominis / Microbacterium laevaniformans / Microbacterium pyrexiae / Microbacterium flavum / Microbacterium paraoxydans / Microbacterium proteolyticum / Microbacterium aerolatum / Microbacterium assamensis / Microbacterium flavescens / Microbacterium foliorum / Microbacterium ginsengiterrae / Microbacterium marinum / Microbacterium oleivorans / Microbacterium radiodurans / Microbacterium resistens / Microbacterium testaceum
Microbacterium sediminis group	Microbacterium sediminis / Microbacterium petrolearium / Microbacterium halimionae / Microbacterium hatanonis / Microbacterium sediminicola / Microbacterium binotii / Microbacterium telephonicum
Microcella putealis group	Microcella putealis / Microcella alkaliphila / Labedella endophytica / Labedella gwakjiensis
Micrococcus antarcticus group	Micrococcus antarcticus / Micrococcus endophyticus / Micrococcus luteus / Micrococcus yunnanensis / Micrococcus aloeverae / Micrococcus cohnii / Micrococcus flavus
Modestobacter marinus group	Modestobacter marinus / Modestobacter muralis / Modestobacter versicolor / Modestobacter caceresii
Mogibacterium diversum group	Mogibacterium diversum / Mogibacterium neglectum / Mogibacterium pumilum / Mogibacterium vescum
Neisseria flava group	Neisseria flava / Neisseria macacae / Neisseria mucosa / Neisseria sicca
Neisseria flavescens group	Neisseria flavescens / Neisseria perflava / Neisseria subflava
Nocardia coeliaca group	Nocardia coeliaca / Rhodococcus degradans / Rhodococcus erythropolis / Rhodococcus qingshengii
Paracoccus aestuarii group	Paracoccus aestuarii / Paracoccus beibuensis / Paracoccus hibisci / Paracoccus marinus / Paracoccus pueri / Paracoccus aeridis / Paracoccus rhizosphaerae / Paracoccus tibetensis / Paracoccus zhejiangensis / Paracoccus alimentarius / Paracoccus isoporae / Paracoccus siganidrum
Paracoccus aestuarii group	Paracoccus aestuarii / Paracoccus beibuensis / Paracoccus hibisci / Paracoccus marinus / Paracoccus pueri / Paracoccus aeridis / Paracoccus rhizosphaerae / Paracoccus tibetensis / Paracoccus zhejiangensis / Paracoccus alimentarius / Paracoccus isoporae / Paracoccus siganidrum
Paracoccus aestuarii group	Paracoccus aestuarii / Paracoccus hibisci / Paracoccus marinus / Paracoccus rhizosphaerae / Paracoccus tibetensis / Paracoccus alimentarius / Paracoccus isoporae / Paracoccus beibuensis / / Paracoccus zhejiangensis / Paracoccus siganidrum

Paracoccus simplex group	Paracoccus simplex / Paracoccus aminovorans / Paracoccus caeni / Paracoccus chinensis / Paracoccus fontiphilus / Paracoccus huijuniae / Paracoccus subflavus / Paracoccus niistensis / Paracoccus aerius / Paracoccus angustae / Paracoccus communis / Paracoccus contaminans / Paracoccus denitrificans / Paracoccus halophilus / Paracoccus sanguinis / Paracoccus speluncae / Paracoccus tibetensis
Paracoccus laeviglucosivorans group	Paracoccus laeviglucosivorans / Paracoccus yeei / Paracoccus carotinifaciens / Paracoccus hibiscisoli / Paracoccus marcusii
Paracoccus sanguinis group	Paracoccus sanguinis / Paracoccus panacisoli / Paracoccus aminovorans / Paracoccus caeni / Paracoccus chinensis / Paracoccus fontiphilus / Paracoccus huijuniae / Paracoccus subflavus / Paracoccus angustae / Paracoccus communis / Paracoccus contaminans / Paracoccus halophilus / Paracoccus simplex / Paracoccus sphaerophysae
Pediococcus stilesii group	Pediococcus acidilactici / Pediococcus stilesii / Pediococcus claussenii
Peptoniphilus gorbachii group	Peptoniphilus gorbachii / Peptoniphilus lacydonensis / Peptoniphilus grossensis / Peptoniphilus harei / Peptoniphilus timonensis / Peptoniphilus phoceensis
Peptoniphilus grossensis group	Peptoniphilus grossensis / Peptoniphilus gorbachii / Peptoniphilus lacydonensis / Peptoniphilus harei / Peptoniphilus timonensis
Phycicoccus bigeumensis group	Phycicoccus bigeumensis / Phycicoccus ginsenosidimutans / Phycicoccus aerophilus / Phycicoccus soli / Phycicoccus dokdonensis
Prevotella histicola group	Prevotella histicola / Prevotella veroralis / Prevotella jejuni
Pseudomonas aylmerense group	Pseudomonas aylmerense / Pseudomonas palleroniana / Pseudomonas tolaasii / Pseudomonas costantinii / Pseudomonas lurida
Pseudomonas aeruginosa group	Pseudomonas aeruginosa / Pseudomonas guezennei / Pseudomonas guangdongensis / Pseudomonas otitidis / Pseudomonas resinovorans / Pseudomonas indica
Pseudomonas antarctica group	Pseudomonas antarctica / Pseudomonas extremorientalis / Pseudomonas fluorescens / Pseudomonas kairouanensis / Pseudomonas kitaguniensis / Pseudomonas meridiana / Pseudomonas poae / Pseudomonas simiae / Pseudomonas trivialis / Pseudomonas extremaustralis / Pseudomonas marginalis / Pseudomonas cerasi / Pseudomonas nabeulensis / Pseudomonas veronii
Pseudomonas argentinensis group	Pseudomonas argentinensis / Pseudomonas cremoricolorata / Pseudomonas fulva / Pseudomonas parafulva / Pseudomonas punonensis / Pseudomonas straminea / Pseudomonas koreensis
Pseudomonas asplenii group	Pseudomonas asplenii / Pseudomonas brassicacearum / Pseudomonas fuscovaginae / Pseudomonas asturiensis / Pseudomonas fluorescens / Pseudomonas synxantha / Pseudomonas versuta / Pseudomonas agarici / Pseudomonas caspiana / Pseudomonas deceptionensis / Pseudomonas fragi / Pseudomonas frederiksbergensis / Pseudomonas putida / Pseudomonas thivervalensis / Pseudomonas vranovensis
Pseudomonas azotoformans group	Pseudomonas azotoformans / Pseudomonas lactis / Pseudomonas paralactis / Pseudomonas synxantha / Pseudomonas libanensis / Pseudomonas mucidolens / Pseudomonas fluorescens / Pseudomonas gessardii
Pseudomonas brenneri group	Pseudomonas brenneri / Pseudomonas fluorescens / Pseudomonas proteolytica

Pseudomonas canadensis group	Pseudomonas canadensis / Pseudomonas fluorescens / Pseudomonas salomonii / Pseudomonas corrugata
Pseudomonas chlororaphis group	Pseudomonas chlororaphis / Pseudomonas fluorescens / Pseudomonas glycinae / Pseudomonas kribbensis / Pseudomonas entomophila / Pseudomonas guariconensis / Pseudomonas mosselii / Pseudomonas sichuanensis / Pseudomonas soli
Pseudomonas corrugata group	Pseudomonas corrugata / Pseudomonas canadensis / Pseudomonas fluorescens / Pseudomonas salomonii
Pseudomonas deceptionensis group	Pseudomonas deceptionensis / Pseudomonas fragi / Pseudomonas lundensis / Pseudomonas psychrophila / Pseudomonas weihenstephanensis
Pseudomonas chloritidismutans group	Pseudomonas chloritidismutans / Pseudomonas knackmussii / Pseudomonas stutzeri / Pseudomonas zhaodongensis / Pseudomonas kunmingensis
Pseudomonas fluorescens group	Pseudomonas fluorescens / Pseudomonas glycinae / Pseudomonas kribbensis / Pseudomonas granadensis / Pseudomonas koreensis / Pseudomonas turukhanskensis
Pseudomonas grimontii group	Pseudomonas grimontii / Pseudomonas marginalis / Pseudomonas rhodesiae
Pseudomonas indoloxydans group	Pseudomonas indoloxydans / Pseudomonas oleovorans / Serratia plymuthica / Pseudomonas sediminis
Pseudomonas cannabina group	Pseudomonas cannabina / Pseudomonas syringae / Pseudomonas cerasi / Pseudomonas congelans / Pseudomonas ficuserectae
Pseudomonas meliae group	Pseudomonas meliae / Pseudomonas savastanoi / Pseudomonas tremae / Pseudomonas cerasi / Pseudomonas chlororaphis / Pseudomonas congelans / Pseudomonas ficuserectae / Pseudomonas protegens / Pseudomonas syringae / Pseudomonas lini / Pseudomonas caricapapayae
Pseudomonas veronii group	Pseudomonas veronii / Pseudomonas extremaustralis / Pseudomonas fluorescens / Pseudomonas marginalis / Pseudomonas antarctica / Pseudomonas extremorientalis / Pseudomonas kairouanensis / Pseudomonas kitaguniensis / Pseudomonas meridiana / Pseudomonas nabeulensis / Pseudomonas poae / Pseudomonas simiae / Pseudomonas trivialis
Pseudomonas vranovensis group	Pseudomonas vranovensis / Pseudomonas alkylphenolica / Pseudomonas asplenii / Pseudomonas fuscovaginae / Pseudomonas hutmensis
Rhodopseudomonas pentothenatexigens group	Rhodopseudomonas pentothenatexigens / Rhodopseudomonas thermotolerans / Rhodopseudomonas faecalis / Rhodopseudomonas palustris
Sphingomonas alpina group	Sphingomonas alpina / Sphingomonas echinoides / Sphingomonas oligophenolica / Sphingomonas asaccharolytica / Sphingomonas insulae / Sphingomonas kyungheensis / Sphingomonas mali / Sphingomonas mucosissima / Sphingomonas panacis / Sphingomonas populi / Sphingomonas pruni / Sphingomonas aquatilis / Sphingomonas dokdonensis / Sphingomonas jeddahensis / Sphingomonas melonis
Staphylococcus aureus group	Staphylococcus argenteus / Staphylococcus aureus / Staphylococcus schweitzeri / Staphylococcus simiae / Staphylococcus haemolyticus / Staphylococcus petrasii

Staphylococcus caeli group	Staphylococcus caeli / Staphylococcus pseudoxylosus / Staphylococcus saprophyticus / Staphylococcus edaphicus / Staphylococcus xylosus / Staphylococcus arlettae / Staphylococcus gallinarum
Staphylococcus capitis group	Staphylococcus capitis / Staphylococcus caprae / Staphylococcus epidermidis / Staphylococcus saccharolyticus / Staphylococcus cohnii / Staphylococcus haemolyticus / Staphylococcus hominis
Staphylococcus epidermidis group	Staphylococcus capitis / Staphylococcus caprae / Staphylococcus epidermidis / Staphylococcus saccharolyticus / Staphylococcus cohnii / Staphylococcus haemolyticus / Staphylococcus hominis
Staphylococcus haemolyticus group	Staphylococcus haemolyticus group / Staphylococcus petrasii / Staphylococcus hominis / Staphylococcus argenteus / Staphylococcus aureus / Staphylococcus devriesei / Staphylococcus lugdunensis / Staphylococcus schweitzeri / Staphylococcus simiae
Staphylococcus hominis group	Staphylococcus hominis / Staphylococcus haemolyticus / Staphylococcus lugdunensis / Staphylococcus petrasii / Staphylococcus capitis / Staphylococcus caprae / Staphylococcus epidermidis / Staphylococcus pasteuri
Staphylococcus hominis group	Staphylococcus hominis / Staphylococcus haemolyticus / Staphylococcus lugdunensis / Staphylococcus petrasii / Staphylococcus capitis / Staphylococcus caprae / Staphylococcus epidermidis / Staphylococcus pasteurii
Streptococcus mitis group	Streptococcus cristatus / Streptococcus gordonii / Streptococcus gwangjuense / Streptococcus infantis / Streptococcus mitis / Streptococcus oralis / Streptococcus periodonticum / Streptococcus pneumoniae / Streptococcus timonensis / Streptococcus pseudopneumoniae / Streptococcus sanguinis / Streptococcus chosunense
Streptococcus lactarius group	Streptococcus lactarius / peroris / parasanguinis
Streptococcus parasanguinis group	Streptococcus parasanguinis / Streptococcus australis / Streptococcus cristatus / Streptococcus rubneri
Streptococcus salivarius group	Streptococcus salivarius / Streptococcus vestibularis / Streptococcus thermophilus
Variovorax gossypii group	Variovorax gossypii / Variovorax guangxiensis / Variovorax paradoxus
Veillonella parvula group	Veillonella parvula / Veillonela dentocariosa / Veillonella tobetsuensis / Veillonella rodentium / Veillonella rogosae

B)

**Unknown bacteria #**	**Best match in Genbank BLAST search**
Unknown bacteria 1	100% match with uncultured *Vampirovibrio* sp. clone Z3AcetBAC91 16S ribosomal RNA gene, accession number KX350762.1
Unknown bacteria 2	100% match with uncultured Syntrophobacteraceae bacterium clone 327 16S ribosomal RNA gene, accession number KX366231.1
Unknown bacteria 3	99.8% match with Tuber borchii symbiont b-17BO 16S ribosomal RNA gene, accession number AF070444.1

Unknown bacteria 4	100% match with uncultured bacterium clone 3500 16S ribosomal RNA gene, accession number MF082879.1
Unknown bacteria 5	100% match with uncultured bacterium gene for 16S rRNA, clone: 11Aug11-129, accession number LC336118.1
Unknown bacteria 6	99,8% match with uncultured delta proteobacterium clone 4M1_F12 16S ribosomal RNA gene, accession number EU052019.1
Unknown bacteria 7	99,3% match with uncultured bacterium partial 16S rRNA gene, isolate BACT_OTU_235, accession number LT842698.1
Unknown bacteria 8	100% match with uncultured bacterium clone KTB502 16S ribosomal RNA gene, accession number MG388843.1
Unknown bacteria 9	98,6% match with uncultured prokaryote gene for 16S ribosomal RNA, OTU:NOR1708, accession number LC248638.1
Unknown bacteria 10	100% match with Neisseriaceae [G-1] [G-1] bacterium HMT 174, accession number FM873692.1
Unknown bacteria 11	100% match with uncultured bacterium RNA for 16S rRNA, partial sequence, clone: B0423R003_K06
Unknown bacteria 12	100% match with uncultured bacterium partial 16S rRNA gene, OTU05254, accession number LT009308.1
Unknown bacteria 13	97.8% match with uncultured bacterium clone 12-2B-102 16S ribosomal RNA gene, accession number KM221296.1
Unknown bacteria 14	98.8 % match with uncultured bacterium clone lp233 16S ribosomal RNA gene, accession number KC331451.1
Unknown bacteria 15	99.1% match with uncultured bacterium clone 2290 16S ribosomal RNA gene, accession number MF081669.1
Unknown bacteria 16	99.3% match with uncultured bacterium clone OTU424_L_1_A_2109484 16S ribosomal RNA gene, accession number MG858260.1
Unknown bacteria 17	100% match with Desulfatiglans anilini strain WB91 16S ribosomal RNA gene, accession number MH196469.1
Unknown bacteria 18	99.5% match with uncultured bacterium clone S5 16S ribosomal RNA gene, accession number JX133359.1
Unknown bacteria 19	100% match with uncultured bacterium clone OTU1014 16S ribosomal RNA gene, accession number MF689080.1
Unknown bacteria 20	100% match with Myxobacterium AT3-01 gene for 16S rRNA, accession number AB246772.1
Unknown bacteria 21	100% match with uncultured bacterium clone ncd1960f07c1 16S rRNA, accession number JF171142.1
Unknown bacteria 22	98.8% match with unidentified bacterium clone M2_Bulk_T7s_9 16S rRNA, accession number EF605681.1
Unknown bacteria 23	98.5% match with Uncultured bacterium clone Otu2063, accession number MW084188.1
Unknown bacteria 24	99,0% match with Bacterium Kaz2, accession number AB491166.1
Unknown bacteria 25	99.8% match with uncultured bacterium clone KD_68, accession number HQ911196.1
Unknown bacteria 26	100% match with uncultured bacterium partial 16S rRNA gene, clone FG34B-43, accession number FR846901.1
Unknown bacteria 27	98.8 % match with uncultured bacterium clone OTU1241_Y_10_A_1708905, accession number MG859032.1
Unknown bacteria 28	100% match with uncultured bacterium clone SupSIB047, accession number MW128096.1
Unknown bacteria 30	92,6% match with uncultured organism clone KBTEX_233 genomic sequence, accession number MN079308.1
Unknown bacteria 31	100% match with uncultured alpha proteobacterium partial 16S rRNA gene, isolate BACT_OTU_1497, accession number LT842726.1

Unknown bacteria 32	100% match with uncultured bacterium partial 16S rRNA gene, isolate BACT_OTU_2798, accession number LT844046.1
Unknown bacteria 33	99,8% match with uncultured bacterium clone OTU276, accession number MG928809.1
Unknown bacteria 34	94,6% match with uncultured Spirosoma sp. clone OTU627 16S ribosomal RNA gene, accession number MW144078.1
Unknown bacteria 35	100% match with uncultured Planctomyces sp. clone 507, accession number MF042869.1
Unknown bacteria 36	99,5% match with uncultured bacterium isolate DGGE gel band 03_M3 clone 05, accession number JX986140.1
Unknown bacteria 37	100% match with uncultured bacterium clone ncd2030b12c1, accession number JF175604.1
Unknown bacteria 38	99,3% match with uncultured Chitinophagaceae bacterium clone CNY_00868, accession number JQ400929.1
Unknown bacteria 39	99,8% match with uncultured bacterium clone KTTB22, accession number MG392744.1
Unknown bacteria 40	98,0% match with uncultured bacterium clone OTU7082, accession number KT790215.1
Unknown bacteria 41	93,9% match with uncultured bacterium clone 16S(V3+V4)-356 16S ribosomal RNA gene, accession number MH096412.1
Unknown bacteria 42	100% match with uncultured bacterium clone 7A_10-029, accession number KY190683.1
Unknown bacteria 43	100% match with Uncultured Gemmatimonas sp. clone CNY_00734, accession number JQ400828.1
Unknown bacteria 44	99,3% match with uncultured Kofleriaceae bacterium clone 414, accession number KX366318.1
Unknown bacteria 45	99,8% match with uncultured bacterium clone OTU8133, accession number KT791146.1
Unknown bacteria 46	98,8% match with uncultured bacterium clone OTU_6667, accession number MH530315.1
Unknown bacteria 47	100% match with uncultured bacterium clone OTU1315_Control_T1.3100, accession number MF950297.1
Unknown bacteria 48	100% match with uncultured bacterium clone KTTB699, accession number MG393421.1
Unknown bacteria 49	100% match with uncultured actinobacterium partial 16S rRNA gene, clone UMAB-cl-58, accession number FN811242.1
Unknown bacteria 50	100% match with uncultured bacterium clone 3-200, accession number KC554166.1
Unknown bacteria 51	99,8% match with uncultured bacterium clone KTB2725, accession number MG391066.1
Unknown bacteria 52	99,3% match with uncultured bacterium partial 16S rRNA gene, isolate BI-2-66, accession number HG970682.1
Unknown bacteria 53	98,6% match with uncultured Bacteroidetes bacterium partial 16S rRNA gene, clone 169-1, accession number AJ871244.1
Unknown bacteria 54	100% match with uncultured bacterium clone MS-9-5 16S ribosomal RNA gene, accession number KX284677.1
Unknown bacteria 55	100% match with uncultured bacterium clone OTU39 16S ribosomal RNA gene, accession number MW143612.1
Unknown bacteria 56	99,3% match with uncultured bacterium clone 1016 16S ribosomal RNA gene, accession number MG716571.1
Unknown bacteria 57	99,5% match with Candidatus Saccharibacteria bacterium isolate NC_groundwater_1927_Pr3_S-0.2um_48_6 chromosome, accession number CP066696.1
Unknown bacteria 58	99,3% match with uncultured bacterium clone ELA_111314_OTU_7272 16S ribosomal RNA gene, accession number KY522290.1

Unknown bacteria 59	100% match with uncultured bacterium clone OTU333_L_2_A_979036 16S ribosomal RNA gene, accession number MG858172.1
Unknown bacteria 60	100% match with uncultured bacterium clone denovo16199 16S ribosomal RNA gene, accession number MG910600.1
Unknown bacteria 61	98,8% match with uncultured bacterium partial 16S rRNA gene, isolate M1-261, accession number HE653888.1
Unknown bacteria 62	100% match with uncultured bacterium clone OTU7317 16S ribosomal RNA gene, accession number KT790439.1
Unknown bacteria 63	97,6 % match with uncultured bacterium clone ELA_111314_OTU_6424 16S ribosomal RNA gene, accession number KY521460.1
